# Exploration of refined humane endpoints for melioidosis in BALB/c mice

**DOI:** 10.1038/s41684-025-01667-5

**Published:** 2026-01-06

**Authors:** Michael P. Harris, Kay B. Barnes, Thomas R. Laws, Emily May, Michelle Nelson, Sarah V. Harding, Thomas C. Maishman

**Affiliations:** 1 Defence Science and Technology Laboratory, Porton Down, Salisbury, UK; 2 Respiratory Sciences, University of Leicester, Leicester, UK

**Keywords:** Lab life, Bacterial host response

## Abstract

The development of humane endpoints is critical for refining scientific studies involving animals. Body weight and clinical signs of disease data collected in four recent studies assessing medical countermeasures for utility against the disease melioidosis in mice were further analyzed. Here we used this information to ascertain whether a suitable alternative humane endpoint could be identified. A total of 66 possible alternative humane endpoints were explored, which varied the threshold values of the ‘percentage body weight loss post-challenge’ and ‘the clinical signs over time’ following cessation of treatment. The findings indicated a suitable alternative endpoint of a percentage weight loss threshold of 25%, and/or using an average total clinical signs score ≥5 over a 48-h period. This endpoint resulted in a sizeable reduction in median ‘sign-days’ (total clinical score multiplied by the number of days remaining in study) per mouse of 21 days (ranging from 8 to 56 between studies), while maintaining 100% sensitivity and 93% specificity (ranging from 79% to 97% between studies). In addition, the risk of altering the scientific outcome of each study remained low when utilizing this new endpoint. In conclusion, current humane endpoints in this setting can be refined without negatively impacting the key study findings.

## Abstract (web summary)

The authors analyzed four melioidosis studies in mice to identify refined humane endpoints (25% weight loss and/or clinical score ≥5) that improve animal welfare while maintaining study integrity.

## Main

Melioidosis is a disease caused by the bacterium *Burkholderia pseudomallei*, a Gram-negative organism found in tropical and subtropical regions of the world^1^. The global burden of human melioidosis is substantial, with an estimated 165,000 cases worldwide and 89,000 deaths per year^2^. *B. pseudomallei* is intrinsically resistant to many antimicrobials and has a large range of virulence factors allowing it to avoid the host immune response, therefore making it challenging to treat^3^. Currently, the recommended treatment consists of intravenous antibiotics for 10–14 days, followed by an oral eradication phase, with a total treatment duration of 20 weeks^4^. Despite successful completion of the antibiotic regimen, relapse of infection can occur in up to 23% of cases and is associated with a mortality similar to that of the initial infection^5^. It is therefore essential that alternative treatments or treatment strategies are investigated. For the use of new treatments in humans, regulatory authorities currently require that they be demonstrated to be safe and effective. Preclinical evaluation of these new treatments involves the use of animal models to demonstrate efficacy. To be able to evaluate new treatments effectively, animal infections that model human disease are necessary. Rodent models of melioidosis are well described^6–9^, and mouse models have been extensively used to characterize the pathogenesis of melioidosis. The presentation of disease depends not only on the route of infection but also on the strain of the mouse. BALB/c mice are more susceptible to infection with *B. pseudomallei* and represent an acute model of melioidosis, whereas C57BL/6 mice are more resistant and may represent a more chronic model of disease^6^. Despite the infection in the BALB/c mouse model being acute, it is considered an appropriate model for evaluating the efficacy of antibiotics^8^. Studies have demonstrated that BALB/c mice infected with *B. pseudomallei* can be effectively treated with antibiotics, with 100% survival at the end of the treatment period and no detection of bacteria within their organs^10,11^. However, relapse to infection is often observed following the cessation of antibiotic therapy^10,11^. Relapse is usually observed from 7 days after the cessation of therapy, with weight loss most commonly observed first, followed by the development of clinical signs of disease, which gradually increase until a humane endpoint is reached.

In four recent studies, named study 1^11^, study 2 (unpublished), study 3^12^ and study 4^13^, antibiotics were evaluated as monotherapies (finafloxacin, doxycycline or co-trimoxazole) and as combinations (finafloxacin in combination with doxycycline and finafloxacin in combination with a capsular conjugate vaccine). All four studies used the same primary outcome measure: time to lethal endpoint. In addition to protection being the primary parameter measured, additional data were collected on body weight and clinical signs of disease. Although these mice were euthanized by cervical dislocation once they reached their predefined humane endpoint (as required under the Animals Scientific Procedures Act^14^), some animals still succumbed to infection (Table 1). Alternative humane endpoints could therefore be explored to both reduce the likelihood of animals succumbing to infection before euthanasia and minimize the potential suffering of those exhibiting clinical signs. This refinement is an important component of the 3Rs (replacement, reduction and refinement) principles to “minimize the pain, suffering, distress or lasting harm that may be experienced by research animals, and which improve their welfare”^15^.

**Table 1:** Summary of data collected from each of the four studies

Parameter	Study 1^11^	Study 2^a^	Study 3^12^	Study 4^13^
Number of mice	105	110	135	206
Number (%) reaching predefined end of study	67 (63.8%)	92 (83.6%)	116 (85.9%)	123 (59.7%)
Number (%) reaching humane endpoint	34 (32.4%)	16 (14.5%)	19 (14.1%)	76 (36.9%)
Number (%) succumbing to infection before being euthanized	4 (3.8%)	2 (1.8%)	0 (0%)	7 (3.4%)
Study duration (post-challenge in days)	66	53	43	36
Number of treatment comparison groups	3	2	4	3
Challenge dose (mean retained dose in CFU)	142	62	100	106
Treatment duration (days)	14	14, 14^b^	14	7
Treatment start time (h)	24	24	24 or 36	36 or 48

^a^Manuscript in preparation; ^b^14 days followed by 14 days ‘pause’, followed by a further 14 days.

A preliminary analysis, in the form of a week-long hackathon involving individuals from a variety of disciplines, took place in January 2023. The aim of the hackathon was to try to identify which parameters were most closely associated with the animals (mice) that succumbed to infection by *B. pseudomallei*. A wide range of approaches was explored to address this question, including network analyses, decision trees, random forests, recurrent neural networks and a review of the existing literature. The findings indicated that two of the most prominent parameters were consecutive percentage body weight loss post-challenge and the total clinical signs score. These metrics are easy to calculate and are already used in current studies to determine humane endpoints. This preliminary analysis indicated that lowering the total clinical signs score from 6 to 5 (in the period following the cessation of treatment) could be used as a refined humane endpoint. However, the impact on the study findings was not explored at this event, as it was beyond the research scope. This is particularly important because overly conservative approaches that euthanize animals too soon could negatively impact the study findings and, in the extreme, prevent the study from properly answering the research question at hand, thus using animals needlessly^15^.

The aim of this work was to identify a suitable alternative humane endpoint using an optimization approach, that is, by minimizing a cost function derived from measures of potential suffering through the modeling of refined humane endpoints (involving percentage body weight loss and total clinical signs), while minimizing the probability of negatively impacting the key study findings.

## Results

### Exploratory analysis

A summary of the data collected during the four studies is presented in Table 1. Each study included over 100 mice. However, the study duration varied across the studies (from 36 to 66 days), challenge dose ranged from 62 to 142 colony-forming units (CFU) and the treatment regimen also varied across studies.

**Fig. 1:**
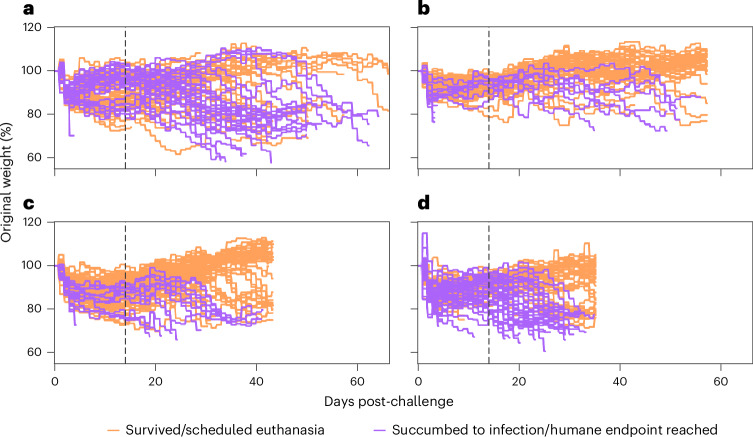
Weight change over time by study. **a**–**d**, Percentage of weight change over time per mouse, separated by survival outcome (survived/experimental euthanasia or succumbed to infection/humane euthanasia) for study 1 (**a**), study 2 (**b**), study 3 (**c**) and study 4 (**d**). The vertical dashed lines at 14 days post-challenge indicate when the analyses were initiated.

Exploratory analysis was conducted on the percentage change in body weight and total clinical signs to illustrate their potential impact on survival. The percentage of body weight change from each animal’s pre-challenge body weight was compared by study and survival outcome, and is illustrated in Fig. 1. Clearly evident from this plot is the difference in study duration, but also the high number of mice in study 1 losing weight compared with the other studies. From day 15 post-challenge, most of the mice in studies 2–4 seemed to maintain a stable weight. However, in all studies, a separation can be seen between the animals that survived compared with those that succumbed to infection, with the latter group showing a larger percentage weight loss.

The change in total clinical signs scores over time, by study and survival outcome, is shown in Supplementary Fig. 1. In this case, there is noticeable variability in the total signs during the treatment period (during the first 7–14 days), after which point they stabilize. Also evident is the impact of using the refined humane endpoint (animals with a consecutive percentage body weight loss ≥30% compared with their pre-challenge weight, or with total clinical signs score of ≥6, were euthanized) in studies 2–4. In study 1 (which did not utilize this refined humane endpoint), a higher proportion of mice with increased total signs is observed compared with the other three studies. In all studies, there also seems to be an emerging separation between the animals that survived compared with those that succumbed to infection, with the latter showing increased total clinical signs; however, this is not as clear a separation compared with that observed for percentage weight change in Fig. 1.

### Impact on study outcome

**Fig. 2:**
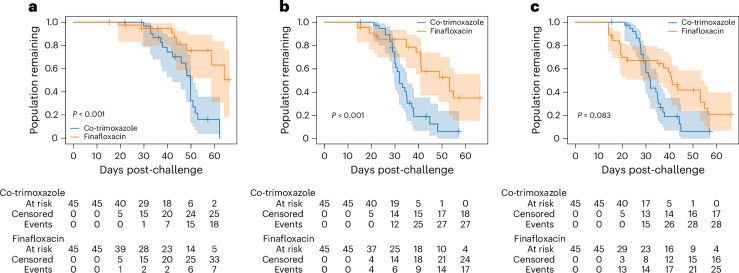
Illustration of weight threshold impact on treatment comparisons. **a**–**c**, A comparison of co-trimoxazole and finafloxacin treatment (study 1 data only) when there is no change in the weight threshold, that is, using the original study conditions in which no body weight threshold was in place (**a**); when using a percentage weight loss threshold of 25% (**b**); and when using a percentage weight loss threshold of 20% (**c**). Lines represent Kaplan–Meier survival estimates with 95% CIs (corresponding shaded areas). *P* values were generated using log-rank tests between groups: co-trimoxazole (*n* = 45) versus finafloxacin (*n* = 45) in each case.

The potential of changing a study outcome by implementing an alternative humane endpoint was first assessed by changing the consecutive percentage weight loss threshold and comparing the results with the original study results. For example, Fig. 2 shows the Kaplan–Meier plots for study 1, comparing co-trimoxazole and finafloxacin under the following scenarios: no change in the weight threshold, that is, using the original study conditions in which no body weight threshold was in place (Fig. 2a); using a percentage weight loss threshold of 25% (Fig. 2b); and using a percentage weight loss threshold of 20% (Fig. 2c). Under the original study conditions, a significant improvement in survival for finafloxacin compared with co-trimoxazole was found (log-rank test *P* < 0.001). Changing the percentage weight threshold to 25% seemed to primarily alter the finafloxacin treatment group, shifting the survival curve to the left, but did not alter the outcome (*P* < 0.001). However, when the percentage weight threshold was changed to 20%, this also primarily affected the finafloxacin treatment group, shifting the survival curve further to the left to the point where the difference between treatment groups is no longer significant (*P* = 0.083). This analysis demonstrates that using the 25% threshold does not significantly impact the study outcome, whereas the 20% threshold negatively impacts the study outcome. Therefore, the 25% weight loss threshold is preferable in this case.

*P* values for comparisons between treatment groups in all four studies were calculated for percentage weight loss thresholds at integer values from 20% to 30%, inclusive. Ratios of these new *P* values divided by the corresponding *P* values from the original study were calculated. Supplementary Fig. 2 presents these ratios (see also Supplementary Table 1), with each point being color-coded to indicate whether the new *P* values and the original *P* values were significant, in each case. There was little to no change (all ratios ~1) at a percentage weight loss threshold of 30%, which is to be expected as this was the default threshold for all studies except for study 1. However, a percentage weight loss threshold of 20% led to a *P* value for the co-trimoxazole and finafloxacin comparison that was almost 300 times higher than that of the original study (from *P* = 0.000617 to *P* = 0.184), which impacted the study to such a degree that the study findings would have been different had this threshold been in place. Therefore, this threshold is unsuitable as an alternative humane endpoint. There are also some observations that would have been different if the threshold was changed from 23% to 27%. In these situations (which compared doxycycline and finafloxacin), the original study comparison yielded a *P* value of 0.0375, compared with *P* = 0.0623 and *P* = 0.0949 for the weight threshold percentages of 23% and 27%, respectively. These *P* values remain similar to the original *P* value, which was close to the 0.05 cutoff.

The impacts on study outcome from applying threshold values of total clinical signs scores ≥4, ≥5 and ≥6 were also explored (with the percentage weight loss threshold of the original studies held constant). Supplementary Fig. 3 illustrates the ratio of *P* values obtained when using a threshold value of ≥4, ≥5 or ≥6 for ‘total signs’ and ‘average total signs’ (see also Supplementary Table 2). There were no changes in significance when using a threshold of ≥6 for either ‘total signs’ or ‘average total signs,’ which is expected, as these are the same as or similar to the existing signs threshold currently in place. Unlike the impact on study outcome observed when changing the weight thresholds, there were three instances where the previous result was not significant (finafloxacin and doxycycline compared with doxycycline as a monotherapy, *P* = 0.206), but following the change in threshold to ≥4 or ≥5 for ‘total signs’ or ≥4 for ‘average total signs’, the comparison yielded a significant *P* value (*P* = 0.00260, *P* = 0.0197 and *P* = 0.0266, respectively). No other changes in significance were found, but larger differences to the *P* values were observed for ‘total signs’ compared with ‘average total signs’, particularly for a signs threshold ≥5.

**Fig. 3:**
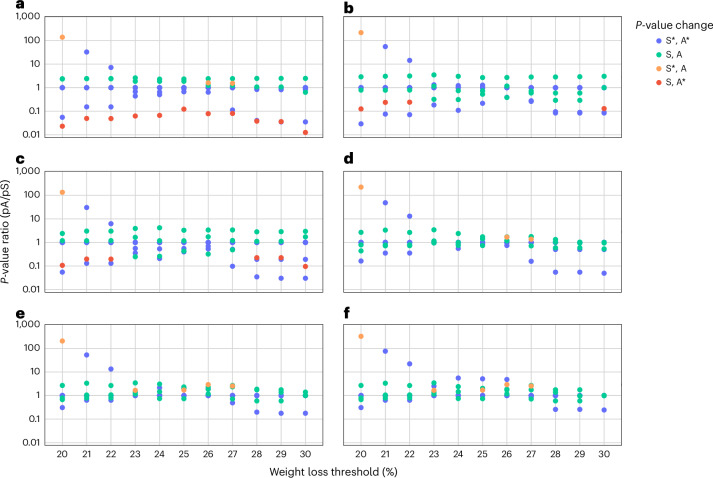
Impact of alternative humane endpoints on study outcomes. **a**–**f**, *P*-value ratios of treatment group comparisons for a combination of clinical sign threshold metrics and weight loss thresholds (data from all four studies). Plots **a**, **c** and **e** use the ‘total signs’ threshold metric. Plots **b**, **d** and **f** use the ‘average signs’ threshold metric. Clinical signs threshold values are illustrated for scores of at least 4 (**a**,**b**), at least 5 (**c**,**d**) and at least 6 (**e**,**f**). The blue points show instances where both the original study comparison (S) and the alternative comparison (A) were significant (S*, A*). The green points show instances where both the original study comparison and the alternative comparison were not significant (S, A). The orange points show instances where the original study comparison was significant but the alternative comparison was not significant (S*, A). The red points show instances where the original study comparison was not significant but the alternative comparison was significant (S, A*).

The next stage of the analysis was to assess the combination of percentage weight threshold and total clinical signs, that is, 66 combinations in total (Fig. 3 and Supplementary Table 3). *P*-value ratios that deviated substantially from 1 were observed at a 20% weight loss threshold value, irrespective of whether ‘total signs’ or ‘average total signs’ was used. The ratios converged toward 1 by the 23% threshold, before diverging again from the 28% threshold. However, only the two comparisons—doxycycline versus finafloxacin, and finafloxacin with doxycycline versus doxycycline monotherapy—showed a change in significance beyond this weight threshold. This change in significance occurred only for the signs thresholds of ≥4 or ≥5 (when ‘total signs’ was used) or ≥4 for ‘average total signs’.

The combination of percentage weight threshold for the ‘average total signs’ of ≥5 was separated by study (Supplementary Fig. 4 and Supplementary Table 3). It is clear that the outcomes that were impacted the most are for study 1 and study 3, which is probably due, at least in part, to the fact that a 30% consecutive weight loss threshold was not in place for study 1 and to the increased number of comparisons made in study 3. Similar to the results overall, the ratios also converged toward 1 by the 23% threshold, before diverging again from the 28% threshold across the four studies.

### Sensitivity and specificity

The next stage of the analysis was to assess the sensitivity and specificity of weight thresholds between 23% and 28%, and cutoff values of ≥4 and ≥5 for ‘total signs’ and ‘average total signs’.

Sensitivity was calculated as 100% for all humane endpoints evaluated, that is, all animals that succumbed to infection or that reached the humane endpoint were correctly identified. This is to be expected given that these humane endpoints are better refined than those used under the original study conditions. Specificity was above 80% for all endpoints assessed overall (Supplementary Fig. 5), that is, the majority of animals that did not succumb and reached the end of the study or scheduled euthanasia were correctly identified. Specificity was notably higher for less strict endpoints (higher weight and sign threshold values). In addition, specificity was above 90% for the ‘average total signs’ threshold of ≥5 endpoints assessed overall.

### Median days saved and median sign-days saved

**Fig. 4:**
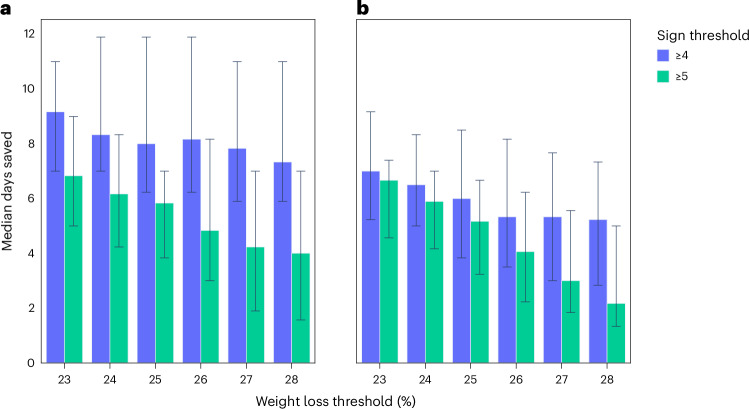
Impact of alternative humane endpoints on time in study. **a**,**b**, ‘Median days saved’ per mouse across humane endpoints derived according to weight loss threshold (*x* axes) and clinical sign threshold (blue/green color), using ‘total signs’ metric (**a**), or ‘average total signs’ metric (**b**) (data from all four studies used; *n* = 556 mice). The error bars represent the 95% CIs for the ‘median days saved’.

The final stage of the analysis was to assess the potential reduction in suffering (that is, time saved) for animals if a refined humane endpoint was used compared with the original study conditions. This was explored for the same humane endpoint considerations as assessed for sensitivity and specificity. Figure 4 shows the reduction in suffering in terms of the ‘median days saved’ per animal, which was positive for all refined endpoints and, unlike the observed specificity, was largest for stricter endpoints (lower weight and sign threshold values, and incorporating ‘total signs’). In addition, for a sign threshold of ≥5, there appeared to be a reduction in the ‘median days saved’ as the weight threshold increased, compared with a sign threshold of ≥4.

However, when assessing the ‘median sign-days saved’ (Supplementary Fig. 6), the rate of decline was more gradual, particularly when using a sign threshold of ≥4. From the 23% to 25% weight loss thresholds, a median of ~20 sign-days was saved per animal, irrespective of the sign threshold and total signs metric type. This is an important finding as it was not apparent when analyzing the ‘median days saved’.

**Fig. 5:**
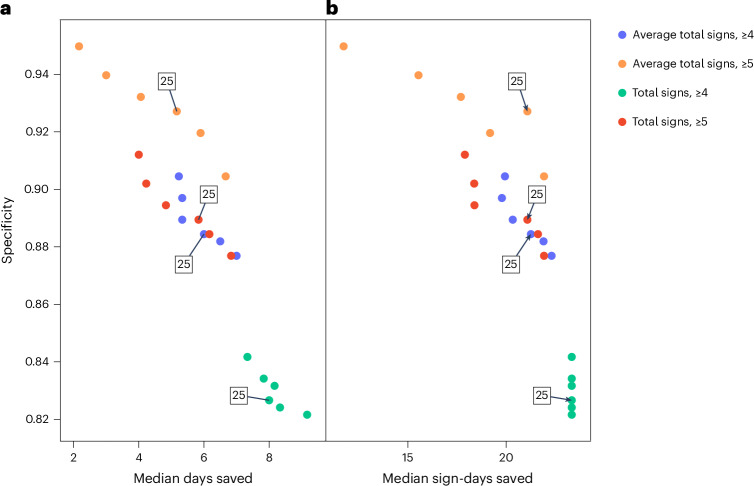
Optimization illustration of alternative humane endpoints on accuracy and time in study. **a**,**b**, Specificity plotted against ‘median days saved’ (**a**) and ‘median sign-days saved’ (**b**), across the humane endpoints evaluated and derived according to weight loss threshold values, grouped by total signs and average total signs threshold values (data from all four studies). Each point represents an alternative humane endpoint, color-coded by clinical threshold value and corresponding to weight thresholds ranging from 23% to 28%. Labeled points illustrate the 25% weight threshold values, which lie in the middle of each color-coded group.

Figure 5 illustrates the specificity against the ‘median days saved’ and ‘median sign-days saved’, to help identify an optimal refined endpoint. A clear set of outliers in this figure, for both ‘days saved’ metrics, is the ‘total signs’ threshold of ≥4 group, which provided lower specificity scores compared with the other sign thresholds. As shown in Supplementary Fig. 3, this refined humane endpoint also demonstrated a notable negative impact on the study findings.

This combination of outliers was therefore ruled out as a suitable alternative humane endpoint. Figure 5 suggests that all other refined humane endpoint combinations resulted in a median reduction of at least 14.5 sign-days per mouse (for example, showing signs of 5 for almost 3 days, or showing signs of 1 for nearly 15 days). However, there was a balance between specificity and the reduction in potential suffering as the humane endpoints became stricter. An approximately linear reduction in specificity as sign-days saved increased is observed from days 15 to 22 post-challenge, with specificity reducing from 95% to 90%. A similar trend is also observed for ‘median days saved’. Combining these results with the impact on the original study findings indicated that the ‘average total signs’ threshold of ≥5 provided the most suitable refined endpoint. Including a weight threshold of 25% provided a specificity of 92.7% and a ‘median sign-days saved’ of 21.1 days; this threshold is also within the center range of the filtered weight threshold range, which allows for a margin of error either side. This is particularly useful when considering weight thresholds below 23%, which were shown to have a marked negative impact on the study findings.

A more comprehensive set of results is presented in Supplementary Table 4. This table shows a summary of results across the humane endpoints evaluated and derived according to weight loss threshold values (between 23% and 28%), grouped by study, total signs and sign threshold values (≥4 or ≥5). A drop in specificity scores for study 1 is evident, reaching as low as 0.761 in several of the ‘total signs’ threshold rows. Although these rows exhibited the largest ‘median days saved’ and ‘median sign-days saved’, the corresponding number of mice that would have been euthanized prematurely was highest (also reflected by the drop in specificity), which limits their suitability as an alternative humane endpoint. The ‘average total signs’ threshold of ≥5 provided the highest specificity ranges for study 1, and although the ‘median days saved’ and ‘median sign-days saved’ were lower, these nonetheless presented a considerable potential reduction in suffering. Also noticeable from these results is that, in all cases for study 1, no changes in outcomes were observed.

Despite generally higher specificity and lower ‘median days saved’/‘median sign-days saved’ values, a similar pattern of results was also found for study 2. The ‘average total signs’ threshold of ≥5 provided the highest specificity ranges with a noticeable potential reduction in suffering (particularly for ‘median sign-days saved’) and without negatively impacting the study findings.

For study 3, despite a similar pattern of results emerging, a key difference in these results was the number of changes in outcomes that occurred, particularly for the rows corresponding to ‘total signs’ threshold ≥4, in which up to 2 (33.3%) of the 6 treatment comparisons changed in outcome.

Study 4 showed the lowest ‘median days saved’ and ‘median sign-days saved’ values among all the study results. However, this still presents with potential reduction in suffering. Again, the ‘average total signs’ threshold of ≥5 provided the highest specificity ranges, with up to 4.2 ‘median days saved’ (2.5 for the 25% weight threshold) and up to 10.9 ‘median sign-days saved’ (7.8 for the 25% weight threshold) and without negatively impacting the study findings.

## Discussion

The aim of this work was to determine whether the humane endpoint used in BALB/c mouse studies evaluating treatments for melioidosis could be further refined. In total, 66 possible alternative humane endpoints were investigated. These involved varying the percentage body weight loss compared with the pre-challenge threshold values in combination with clinical signs of disease over time, to determine the value by which an associated threshold could be established. Ultimately, a weight loss threshold of 25%, combined with a mean total clinical signs score of ≥5 over a 48-h period, was identified as the most suitable refined humane endpoint. This endpoint provided a compromise between maximizing the median sign days saved and having a high specificity, without negatively impacting the study findings.

BALB/c mice have been used in-house for several decades to assess treatments for melioidosis^8,10–13,16,17^. In keeping with regulatory guidelines, humane endpoints have been used and refined extensively over time. The humane endpoint for *B. pseudomallei* infection in BALB/c mice, predominantly used in the studies analyzed here, occurs when an animal reaches a weight loss of 30% of its baseline weight, and/or exhibits a pronounced reduction in activity, labored breathing, a total score of 6 or any indication of neurological signs—whichever occurs first. This endpoint has evolved, based on experience and physical observations of the animals over many years. However, the analysis in this study examined the humane endpoint for *B. pseudomallei* infection holistically using statistical methods. Although previous studies suggested that humane endpoints may be model-specific^18^, sophisticated approaches such as machine learning have been able to generalize alternative approaches across a range of different model types^19^. Indeed, trained machine learning approaches have identified refined humane endpoints for two distinct models, a sepsis and a stroke model in mice^19^. The analysis reported here focused on a defined infection caused by *B. pseudomallei* and a defined mouse model (BALB/c). Although the studies included in this work were restricted to a specific pathogen, the themes and methods could be used in other settings. However, it is important to note that a body of data would be required to validate the approach.

The study presents a number of potential limitations which should be addressed. First, it is important to note that data from four distinct *B. pseudomallei* treatment studies were used in this analysis, each with different treatment regimens, variations in challenge dose and different study durations. However, sizeable effort was made to ensure the studies were as comparable as possible and to mitigate any potential issues. These efforts include ensuring that specific parameters (treatment regimen or challenge dose) were not incorporated into the refined humane endpoint definitions and that over 100 mice were included in each study, thereby yielding a considerable amount of data, which somewhat accounts for the heterogeneity in treatment regimen, challenge dose and study duration. Despite the differences, the key biological readouts for all studies were that the challenge resulted in a lethal infection in untreated animals and that the infection was at least initially treatable with the antibiotic; that is, all four studies shared the same primary outcome measure of time to lethal endpoint. These similarities allowed for the identification of a generic humane endpoint for these studies. The results presented in Supplementary Table 4 also support this aim, by showing that the increase in ‘median sign-days saved’ without negatively impacting the study was consistent across the studies for the ‘average total signs’ threshold of ≥5 with 25% weight loss, despite a drop in specificity for study 1 (which also provided the largest potential reduction in suffering). While the heterogeneity of the data strengthens the conclusions drawn from these analyses, it is important to note that their applicability to other melioidosis studies in BALB/c mice should be limited to studies with comparable challenge doses and treatment regimens.

The humane endpoint parameters used in this analysis, clinical signs and body weight, are commonly used to identify humane endpoints for infectious diseases^20^. However, temperature readouts, which are frequently used in infectious disease studies^21–23^, were not included, representing another potential limitation of these studies. At the time these studies were conducted, early versions of the temperature microchips were not only large in size but also produced variable data readings. Consequently, these microchips were not used in these studies. Clinical signs are overtly linked with disease progression but can be subjective despite training and objective guidelines. Combining and averaging these scores as a total and/or average score could potentially reduce the impact of this subjectivity, particularly when assessing the score for a group of animals. It should also be noted that total signs are already used to define endpoints for *B. pseudomallei* infection studies^8,10–13^. The impact of subjectivity could be reduced further by averaging the mean clinical score over a 48-h time period.

Although body weight is an objective measure, it is important to note that it was recorded only once per day, whereas clinical signs for animals with worsening health were recorded as frequently as every 4 h. To assess the impact of both percentage weight loss and clinical signs, the weight data were analyzed as the last observation carried forward until the next weight recorded, that is, that weight was considered to be constant for the 24-h period leading up to the next weight measurement. The existing humane endpoints also rely on this assumption, and it is equally important to note that the refined humane endpoints downselected for consideration here are based on either a substantial reduction in percentage weight loss and/or a substantial increase in clinical sign scores (whichever occurs first). As a result, the refined humane endpoints proposed are more conservative than those currently in place. Utilizing a combination of weight loss and signs is also needed to more accurately differentiate between animals that survive and those that succumb to infection (as shown in Supplementary Fig. 1, which illustrates that total signs alone do not clearly distinguish between these outcomes).

Another potential limitation of this study is the use of a ratio of *P* values to illustrate the impact of the alternative humane endpoints on the study outcomes. This is an unconventional statistical approach, reflected by the lack of literature^24^. It should be noted that the application of this approach is primarily to aid in the visualization of the extent to which the outcomes could differ as variables change, for example, as the weight threshold values increased. This approach does, however, raise another potential limitation of the study, which is its exploratory nature, with a series of multiple tests carried out at the post-hoc analysis stage, without the incorporation of an adjustment for multiple testing. These studies were able to achieve their a priori study hypothesis, which is not in question under this study. However, this particular study has focused on exploring the extent to which the effect would have changed (or remain unchanged) under the alternative humane endpoints tested. The unadjusted *P* values are presented to show the full extent to which the original hypothesis could have changed or been affected.

‘Median sign-days saved’, defined as the reduction in the product of clinical signs and time, was identified as an important way to express the benefit of the refined humane endpoints. It is worth noting that clinical signs data are ordinal and not linear; therefore, they are not a perfect parameter. However, an animal showing clinical signs is likely to have more pronounced infection than a mouse with no signs for the same time period, which is not taken into account when ‘median days saved’ is used. Indeed, other researchers have used similar products, such as the product of body weight and temperature, to increase the accuracy of humane endpoints^18^.

The use of 80% specificity as a suggested cutoff is also a potential limitation of the study, as it may require larger sample sizes for studies compared with using a higher specificity (for example, 90%). However, this criterion was chosen alongside other metrics such as ‘median sign-days saved’, as this could potentially justify the slight increase in sample size to potentially reduce animal suffering without negatively impacting the study findings. In addition, it should equally be noted that the suggested alternative humane endpoint of ‘average total signs’ threshold of ≥5 with 25% weight loss provided a specificity of over 90% overall.

One metric that was not taken into account in this analysis was the presence of bacterial load in the tissues of survivors at the end of the study; bacterial load information could only be measured post-mortem, and it was therefore not possible to use this information as a measure to reduce potential suffering. For example, an animal may have a high bacterial load in tissues at the end of the study without exhibiting severe clinical signs or weight loss, and therefore it would not have reached a humane endpoint within the duration of the study. However, this animal, which would have had the potential to succumb in a short time frame, would give a false negative result. This case is unlikely to be identified through a noninvasive refined humane endpoint approach. This example highlights the balance struck between minimizing the experimental time in the study and maximizing the specificity. For example, the level of specificity observed may be considered unacceptable despite the increase in ‘median sign-days saved’ and the low probability of negatively impacting the findings of the four studies discussed in this work. Conversely, maximizing the ‘median sign-days saved’ may still be considered worthwhile despite the lower specificity of ~80% and increased probability of negatively impacting the study findings. The lower specificity of 80% suggests that 20% of the animals that survived until the end of the experiment would have been incorrectly removed from the study as they had reached this alternative humane endpoint. The bacterial load data for each animal were analyzed to help clarify the impact, in terms of their predictive accuracy, of these alternative humane endpoints accordingly. These data showed that every animal in the 20% specificity group was actually colonized with bacteria, and therefore would have eventually succumbed to infection if the study had run for longer. This observation suggests that, although the specificity is lower than would be ideal, this is less important as in each case the animal was colonized with bacteria, and the gain to animal welfare would justify the use of refined humane endpoints despite the lower specificity. This work aimed to provide a reasonable consensus for a humane endpoint, resulting in a substantial reduction in ‘median sign-days saved’ while maintaining high specificity and a margin of error for the weight threshold to ensure that the study findings were not adversely affected.

Refining the humane endpoint in an animal model without affecting the scientific data generated is important to reduce the amount of time an animal experiences suffering, which is central to the 3Rs. However, there is also an important scientific justification for refining the humane endpoint in BALB/c mouse models of *B. pseudomallei* infection. In humans, it is becoming more apparent that cases of re-infection, and not relapse of infection, are much higher than previously thought^25–27^. Low-level animal-to-animal transmission of *B. pseudomallei* has previously been demonstrated in the BALB/c mouse model^28^. Although the mouse-to-mouse transmission rate observed following an aerosol infection was low (4%), the potential of re-infection should be considered, particularly in studies of long duration. Potential re-infection could indicate false reporting of treatment failure. Mice are social animals and, for ethical reasons, should be housed in social groups. As melioidosis is associated with relapse, study durations can be lengthy (66 days or more) to account for potential relapse after treatment^28^. Once BALB/c mice start to relapse with *B. pseudomallei*, weight loss occurs, accompanied with an increase in clinical scores over time. In the first study, once an animal reached a clinical score of 6 or greater, *B. pseudomallei* could be detected in the urine of some animals^11^. Because the median lethal dose of *B. pseudomallei* strain K96243 in BALB/c mice by the aerosol route is 4 CFU^7^, re-aerosolization of bacteria from the urine could lead to re-infection. Refining the humane endpoint as proposed should remove animals that have relapsed with infection from the study before they reach the point where they are able to re-infect their cage mates. Animal models are useful for evaluating new treatments; however, the constraints of the model should be considered, and it is important to define the outcome of interest. In the BALB/c mouse model of melioidosis, time to relapse could be considered as being more appropriate than survival. This metric is also clinically relevant, as treatment in humans would be restarted at the point of relapse.

## Conclusion

These findings suggest that a percentage weight loss threshold of 25%, coupled with a mean total clinical signs score ≥5 over a 48-h period, is the most suitable refined humane endpoint to use for melioidosis studies in BALB/c mice with comparable challenge doses and treatment regimens. This endpoint provides a substantial reduction in terms of the median number of days in the study, as well as a substantial reduction in the median ‘sign days’ in the study. An added benefit of this endpoint is that it is able to correctly identify which animals will reach their humane endpoint and which will not. Finally, this humane endpoint is able to demonstrate refinement without the risk of altering the key study hypotheses.

## Methods

### Data from previous animal studies

Full details of three of the four studies (studies 1, 3 and 4), including bacterial preparation, animal exposures and treatment regimens, have been previously described^11–13^. Details of the second study (Animal Care and Use Review Office (ACURO) number CB-2016-13) are also provided below. All studies were reviewed and approved by the Defence Science and Technology Laboratory (DSTL) Animal Welfare and Ethical Review Body. All four studies utilized female BALB/c mice that were infected by the inhalational route with *B. pseudomallei* strain K96243.

In summary, in the first study, BALB/c mice were challenged with a mean retained dose of 142 CFU of *B. pseudomallei* by the inhalational route and administered finafloxacin (37.5 mg/kg) every 8 h or co-trimoxazole (78 mg/kg) every 12 h, both delivered by the oral route (Table 1). For all four studies, control groups of infected mice were administered a vehicle (consisting of Tris buffer, sodium hydroxide and hydrochloric acid, adjusted to pH 8) by the oral route every 8 h. Therapy in the first study was initiated at 24 h post-challenge and continued for 14 days^11^.

In the second study (manuscript in preparation), BALB/c mice were challenged with a mean retained dose of 62 CFU of *B. pseudomallei* by the inhalational route. Treatment was initiated at 24 h post-challenge and continued for 14 days; groups of ten mice were administered finafloxacin (23.1 mg/kg or 37.5 mg/kg) by the oral route every 8 h. The efficacy in these groups was compared with groups that were treated for 14 days, followed by a 14-day ‘rest’ period (with no treatment) and a second phase of 14 days of therapy. One group of animals was infected but left untreated.

In the third study, BALB/c mice were challenged with a mean retained dose of 100 CFU of *B. pseudomallei* by the inhalational route and treated from 24 h or 36 h post-challenge, for 14 days with finafloxacin (23.1 mg/kg), doxycycline (100 mg/kg) or both antibiotics, orally, every 8 h (ref. ^12^).

In the fourth study, BALB/c mice were vaccinated three times by the subcutaneous route (on days 0, 21 and 35) with a CPS-CRM197 capsule conjugate (0.25 µg), Hcp1 (0.5 µg), Alhydrogel (250 µg) and CpG (10 µg). Mice were challenged with a mean retained dose of 106 CFU and treated from 36 h or 48 h post-challenge, for 7 days with finafloxacin (23.1 mg/kg), orally, every 8 h (ref. ^13^).

In all four studies, mice were weighed daily and observed at least twice daily for clinical signs of disease, with an increased frequency of observations for any animals with deteriorating clinical signs. Clinical scores were based on the observed changes to the condition of the animals. Mice were euthanized by cervical dislocation once they had reached their humane endpoint.

In studies 1 and 2, a ‘2-score’ criterion was used to assess the severity of clinical signs (see Supplementary Table 5 for clinical scoring criteria). In studies 3 and 4, a ‘3-score’ criterion was used (see Supplementary Table 6 for clinical scoring criteria). The 2-score system was based on changes to six categories (piloerection, posture, eye problems, locomotion, mobility/activity and respiration), with a score of 1 increasing to 2 for worsening conditions in each of the categories. The 3-score system was based on the same six categories, with a score of 1 increasing to 2 and 3 for worsening conditions in each of the categories, or a score of 1 and automatic euthanasia for any severe neurological signs. For the purposes of these analyses, a mapping of the clinical signs from the 3-score criteria to the 2-score criteria was used to make the studies as comparable as possible, including the addition of neurological signs scores from study notes for the two studies that used the 2-score criteria (see Supplementary Table 6 for the mapping used).

In study 1, the humane endpoint was defined as the point at which animals were not expected to recover, specifically if an animal scored 2 in the ‘eyes’, ‘locomotion’ or ‘mobility/activity’ categories of the ‘2-score’ criteria (Supplementary Table 5). However, for studies 2–4, a refined humane endpoint was used at the cessation of antibiotic treatment in line with the 3Rs principles. Animals with a consecutive percentage body weight loss of 30% or more (compared with their pre-challenge weight) or with total clinical signs score of ≥6 were euthanized by cervical dislocation (Supplementary Tables 5 and 6).

All fours studies were reviewed and approved by the DSTL Animal Welfare and Ethical Review Body and were carried out in accordance with the UK Animals (Scientific Procedures) Act 1986, the codes of practice for the Housing and Care of Animals used in Scientific Procedures 1989, and an ACURO Appendix.

### Alternative endpoints investigated

All alternative humane endpoints were calculated from 14 days post-challenge following the treatment period to ensure that treatment was not confounding the weights and signs recorded. The alternative humane endpoints considered were: ‘consecutive percentage body weight loss’, derived as the percentage weight loss compared with their pre-challenge weight over a 48-h period; ‘total signs’ of ≥4, ≥5 or ≥6, calculated by summation of the clinical signs at any one time point; the ‘average total signs’ of ≥4, ≥5 or ≥6, calculated as the mean of the ‘total signs’ value over the preceding 48 h period; a combination of either ‘consecutive percentage weight loss’ or ‘total signs’ (whichever occurred first); or a combination of ‘consecutive percentage weight loss’ or ‘average total signs’ (whichever occurred first). The choice of clinical signs thresholds was decided on the basis of the findings from the hackathon, which identified a score threshold of ≥5 as a possible refined humane endpoint. The additional score thresholds of ≥4 and ≥6 were also assessed to provide a more comprehensive overview of the impact in changing the threshold.

Because the mice were weighed daily, averaging over 48 h allowed two weight readings to be recorded to determine the consecutive percentage weight loss. Similarly, averaging total clinical signs over the same period ensured a comparative measure could be used for total signs.

### Statistical analysis

Information was available for all animals treated with the different treatment regimens to the time to death (or time to humane endpoint euthanasia), denoted as overall survival for simplicity in this Article. Scheduled euthanasia (at predefined time points) were treated as right-censored data. Mice were grouped into key comparator treatment groups across the four studies. Kaplan–Meier survival estimates^29^ for these groups were compared using two-sided log rank tests^30^ (see Supplementary Table 7 for details of the relevant treatment group comparisons, including the number of mice in each group). These comparisons were calculated under existing study conditions, that is, using the humane endpoints currently in place, and for each of the alternative humane endpoints tested. All comparisons were carried out on data left-censored at 14 days post-challenge following the treatment period to ensure that treatment was not confounding the weight and signs recorded. *P* values for the comparison between Kaplan–Meier curves for different groups were calculated using log-rank tests (two-sided), to test for significant differences between treatment groups. The ratios between the *P* values of the corresponding comparisons were used as an indicator for each of the refined humane endpoints, to ascertain which would have the least impact on the study results had they been implemented instead of the existing humane endpoints. Identical *P* values in both cases corresponded to a ratio of 1 and indicated no effect on the study findings, whereas ratios that deviated appreciably from 1 suggested that the findings would have been affected. The extreme cases occurred when the study and modeled *P* values lay on opposite sides of the 0.05 significance threshold, indicating that the modeled findings differed from the study findings. Conversely, where the study *P* value was near 0.05, only a small change in the Kaplan–Meier curves would be required to change a study finding, and therefore a subsequent change in result would be less concerning. No adjustment was made for multiple testing as the aim of these analyses was not to measure the effect of any particular treatment, but instead to ascertain the extent to which the effect would have changed (or remain unchanged) under the alternative humane endpoints tested, for which the unadjusted *P* values are required.

Following the *P*-value ratio analyses, the number of acceptable refined humane endpoints was reduced to identify the most promising to explore further. Sensitivity and specificity were calculated for each of these (overall and by study) to help quantify any adverse effects on the study findings. Sensitivity was defined as the ability of the refined humane endpoint to correctly identify an animal that would succumb to infection or reach their humane endpoint, that is, number of true positives/(number of true positives + number of false negatives). Specificity was defined as the ability of the refined humane endpoint to correctly identify an animal that would not succumb to infection or not reach their humane endpoint, that is, number of true negatives/(number of true negatives + number of false positives). For perfect prediction, the sensitivity and specificity should both be 100%. For both sensitivity and specificity, the corresponding 95% confidence intervals (CIs) were reported.

To estimate the reduction in mice with observed signs of disease by retrospectively implementing refined humane endpoints, two metrics were considered: ‘median days saved’ and ‘median sign-days saved’, calculated using Kaplan–Meier estimates to account for censored data (calculated overall and by study). ‘Median days saved’ reflected the median time difference between predicted and actual survival times, whereas ‘median sign-days saved’ used the same approach but weighted time by the total clinical signs score displayed by the mice during that period—that is, by summing the daily total clinical signs for each day saved. Weighting time by signs provided the ability to differentiate between mice that showed signs of ill health and those that did not. ‘Median days saved’ treats the remaining time in the study the same for all mice irrespective of their health status. In both cases, maximizing the duration of the days saved is considered beneficial for the mice. For both ‘median days saved’ and ‘median sign-days saved’, the corresponding 95% CIs were reported.

The final stage of the analysis was to compare the specificity for the most promising refined humane endpoints against the ‘median days saved’ or ‘median sign-days saved’, to identify endpoints that maximized both these metrics.

All analyses were carried out in Python v3.10.13^31^.

### Reporting summary

Further information on research design is available in the Nature Portfolio Reporting Summary linked to this article.

## Online content

Any methods, additional references, Nature Portfolio reporting summaries, source data, extended data, supplementary information, acknowledgements, peer review information; details of author contributions and competing interests; and statements of data and code availability are available at 10.1038/s41684-025-01667-5.

## Supplementary information

Supplementary Information Supplementary Figs. 1–6 and Tables 1–7.

Reporting Summary

## Data Availability

The original contributions of three of the four studies have been previously published^11–13^. The second study was carried out under a Home Office Project Licence (number P1D46FB69) (manuscript in preparation). The data that support the findings of this study are available from the corresponding author upon request.
