# Supplementary material for: Exploration of refined humane endpoints for melioidosis in BALB/c mice
Source: Lab Anim (NY). 2026 Jan 6;55(2):40–7. doi: 10.1038/s41684-025-01667-5 (PMC12867759; doi:10.1038/s41684-025-01667-5)
Supplement: Supplementary file 1 — Supplementary Figs. 1–6 and Tables 1–7. [file 41684_2025_1667_MOESM1_ESM.pdf]

lab animal

Supplementary information

https://doi.org/10.1038/s41684-025-01667-5

Exploration of refined humane endpoints for melioidosis in BALB/c mice

In the format provided by the authors and unedited

Supplementary Information

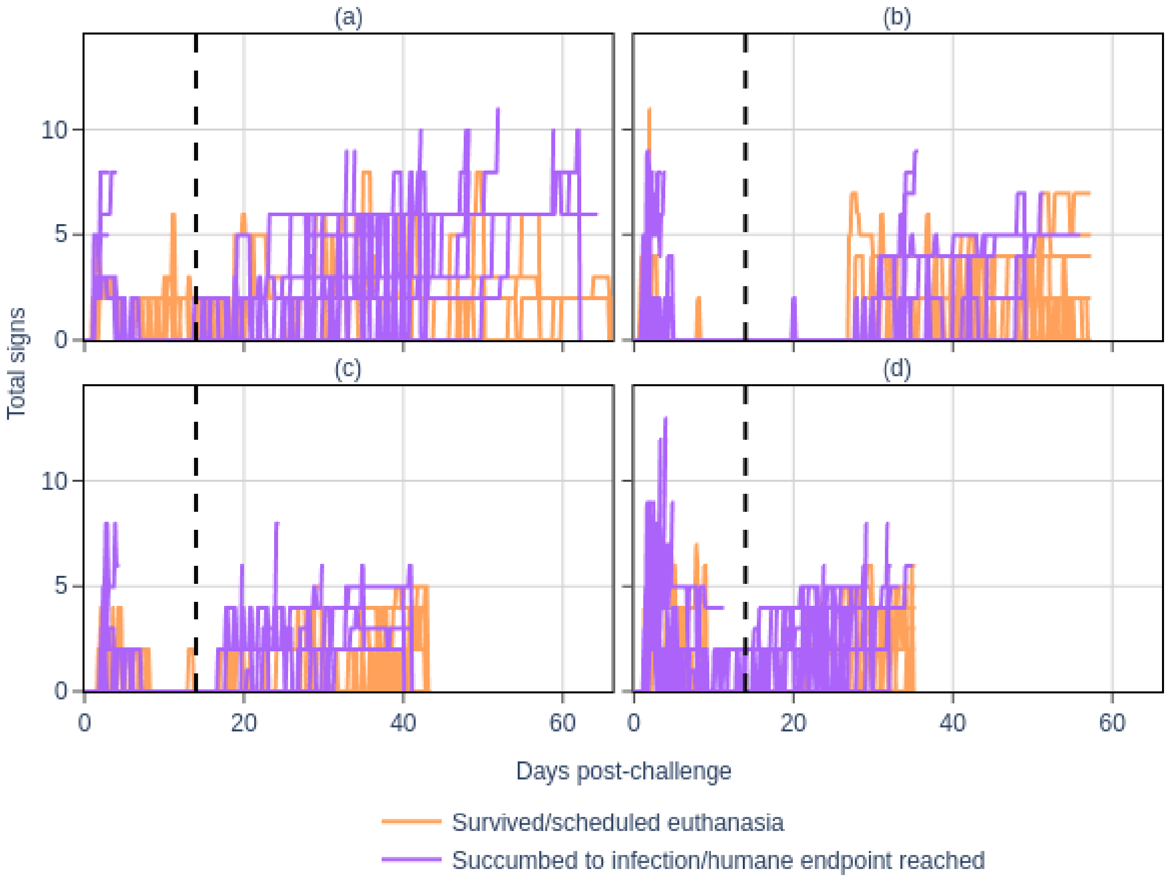

Supplementary Figure 1. Total clinical signs over time by study.
Total clinical signs scores over time per mouse, by survival outcome for (a) Study 1, (b) Study 2, (c) Study 3, and (d) Study 4. The vertical dashed lines are drawn at 14 days post-challenge to indicate when the analyses was initiated.

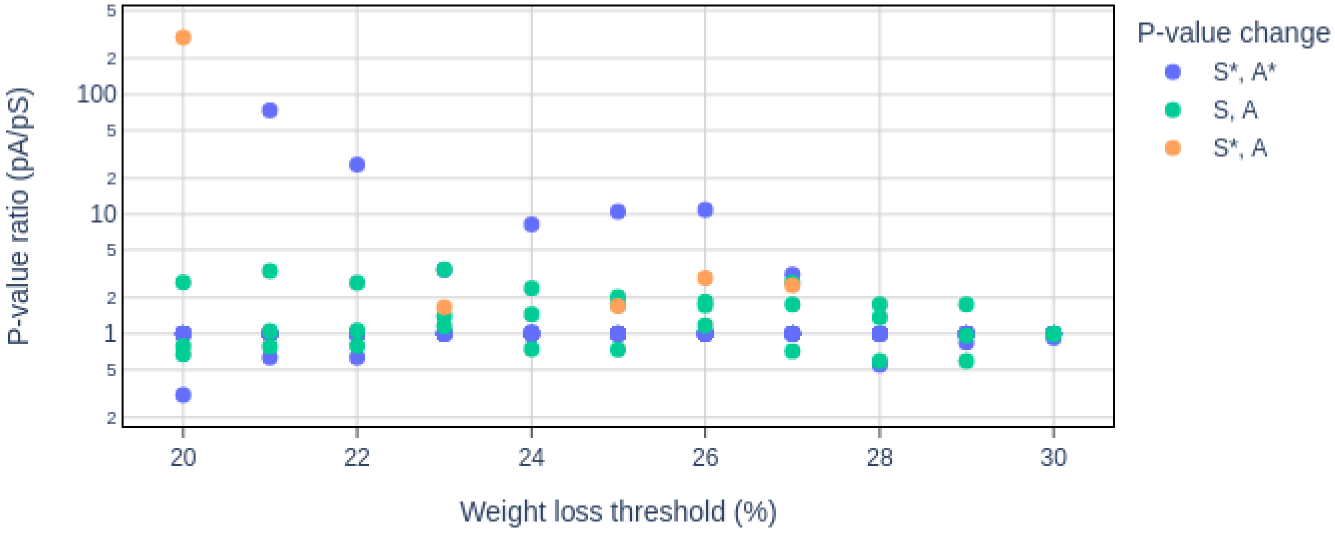

Supplementary Figure 2.
P-value ratios of treatment group comparisons for different weight loss thresholds (without signs-based thresholds applied). Data from all four studies used. The blue points show instances where both the original study comparison (S) and the alternative comparison (A) were significant (S*, A*). The green points show instances where both the original study comparison and the alternative comparison were not significant (S, A).The orange points show instances where the original study comparison was significant but the alternative comparison was not significant (S*, A).

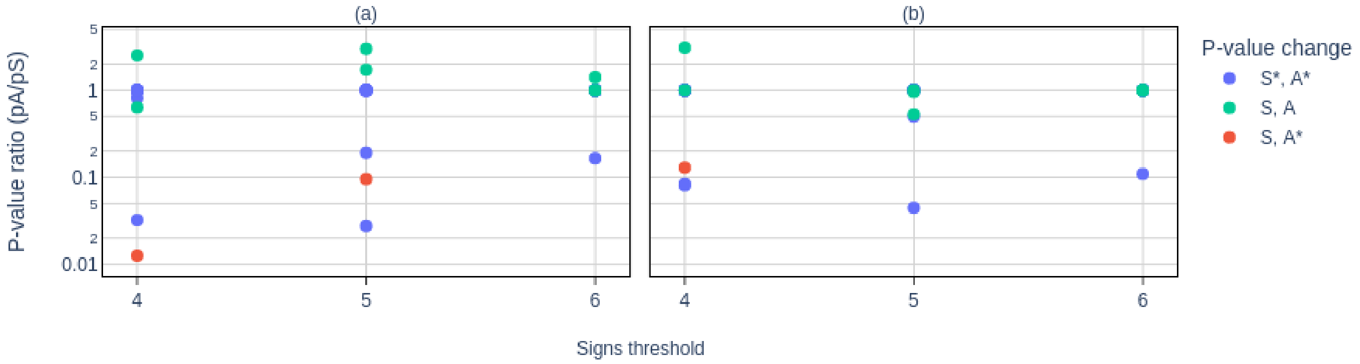

Supplementary Figure 3.
P-value ratios of treatment group comparisons for different sign-based thresholds (without weight loss thresholds applied), for (a) 'total signs' and (b) 'average total signs' (the mean of 'total signs' over the preceding 48h period). Data from all four studies used. The blue points show instances where both the original study comparison (S) and the alternative comparison (A) were significant (S*, A*). The green points show instances where both the original study comparison and the alternative comparison were not significant (S, A).The red points show instances where the original study comparison was not significant but the alternative comparison was significant (S, A*).

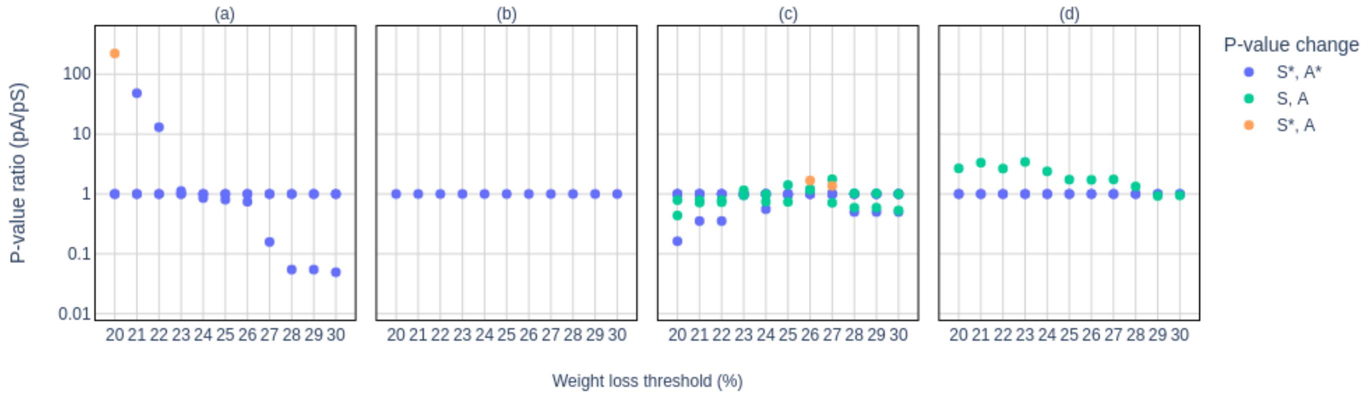

Supplementary Figure 4.
P-value ratios of treatment group comparisons for (a) Study 1, (b) Study 2, (c) Study 3, and (d) Study 4. An average total clinical signs threshold of ≥5 is combined with different weight thresholds. The blue points show instances where both the original study comparison (S) and the alternative comparison (A) were significant (S*, A*). The green points show instances where both the original study comparison and the alternative comparison were <u>not</u> significant (S, A). The orange points show instances where the original study comparison was significant but the alternative comparison was <u>not</u> significant (S*, A).

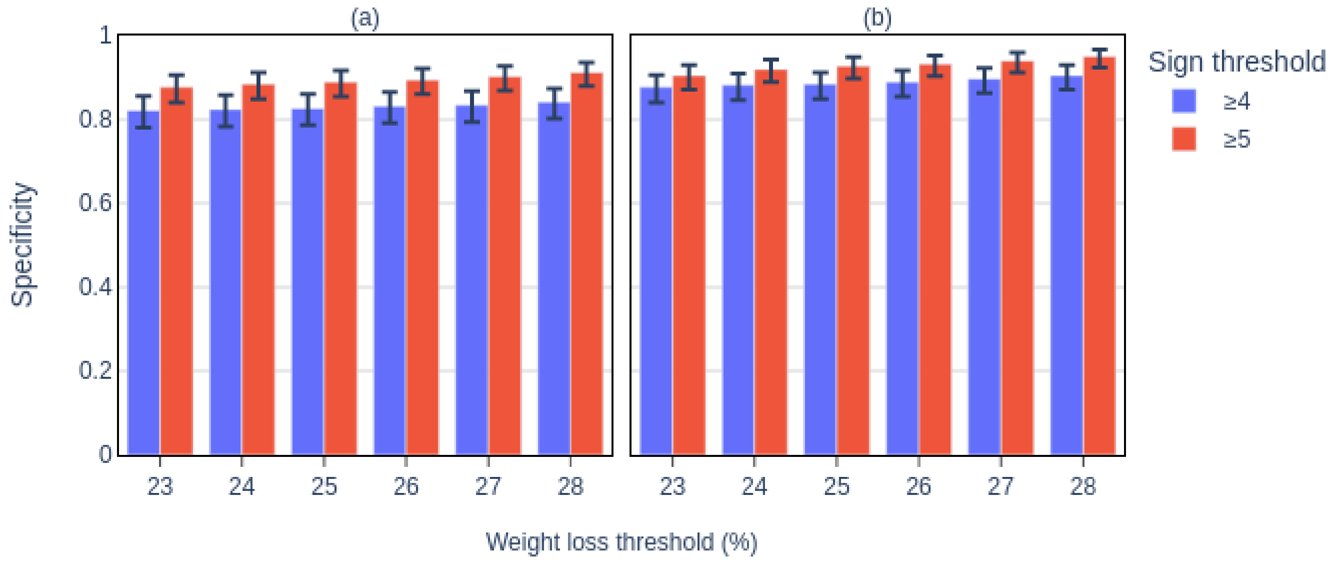

Supplementary Figure 5.
Specificity across refined humane endpoints derived according to weight loss threshold (x-axes), sign threshold (color), and (a) 'total signs' metric, and (b) 'average total signs' metric. Data from all four studies used. The error bars represent the 95% confidence interval for the specificity.

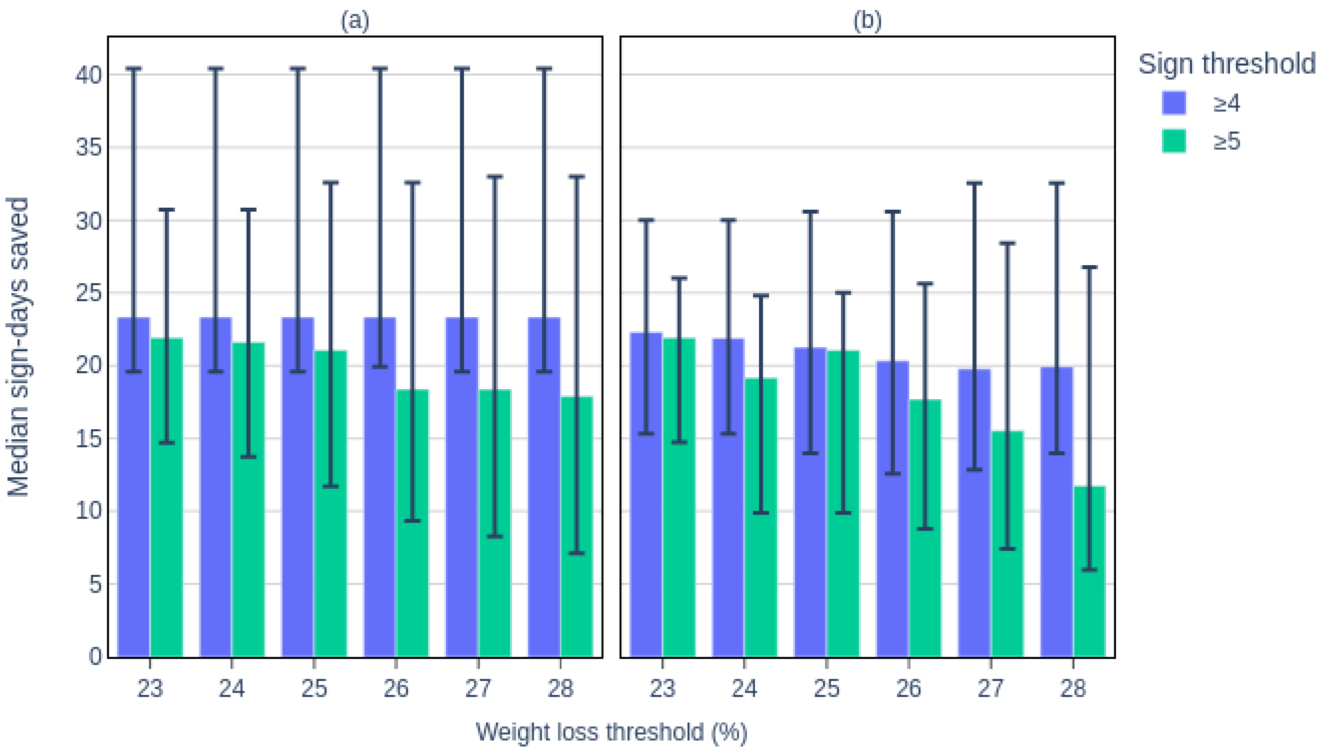

Supplementary Figure 6.
'Median sign-days saved' per mouse across refined humane endpoints derived according to weight loss threshold (x-axes), sign threshold (color), and (a) 'total signs' metric, and (b) 'average total signs' metric. Data from all four studies used. The error bars represent the 95% confidence interval for the 'median sign-days saved'.

Supplementary Table 1.
Logrank test results for the comparisons between treatment groups for each of the four studies, split by the weight threshold value (without signs-based thresholds applied). Information for the logrank test using the alternative endpoint comparison and orginal study comparison are presented, along with the p-value ratio and indicator of whether a change in significance resulted: (S*, A*) denotes instances where both the original study comparison (S) and the alternative comparison (A) were significant; (S, A) denotes instances where both the original study comparison and the alternative comparison were not significant; (S*, A) denotes instances where the original study comparison was significant but the alternative comparison was not significant; and (S, A*) denotes instances where the original study comparison was not significant but the alternative comparison was significant. These data were used for Figure A2.

Study	Weight Threshold	Comparison Group A		Comparison Group B		Alternative endpoint Comparison			Original study comparison			P-value ratio	P-value ratio change
		Treatment	n	Treatment	n	Test statistic	df	p-value	Test statistic	df	p-value		
1	20	Finafloxacin	45	Diluent Control	15	83.192904	1	7.442441E-20	83.192904	1	7.442441E-20	1.000000	S*, A*
1	21	Finafloxacin	45	Diluent Control	15	83.192904	1	7.442441E-20	83.192904	1	7.442441E-20	1.000000	S*, A*
1	22	Finafloxacin	45	Diluent Control	15	83.192904	1	7.442441E-20	83.192904	1	7.442441E-20	1.000000	S*, A*
1	23	Finafloxacin	45	Diluent Control	15	83.192904	1	7.442441E-20	83.192904	1	7.442441E-20	1.000000	S*, A*
1	24	Finafloxacin	45	Diluent Control	15	83.192904	1	7.442441E-20	83.192904	1	7.442441E-20	1.000000	S*, A*
1	25	Finafloxacin	45	Diluent Control	15	83.192904	1	7.442441E-20	83.192904	1	7.442441E-20	1.000000	S*, A*
1	26	Finafloxacin	45	Diluent Control	15	83.192904	1	7.442441E-20	83.192904	1	7.442441E-20	1.000000	S*, A*
1	27	Finafloxacin	45	Diluent Control	15	83.192904	1	7.442441E-20	83.192904	1	7.442441E-20	1.000000	S*, A*
1	28	Finafloxacin	45	Diluent Control	15	83.192904	1	7.442441E-20	83.192904	1	7.442441E-20	1.000000	S*, A*
1	29	Finafloxacin	45	Diluent Control	15	83.192904	1	7.442441E-20	83.192904	1	7.442441E-20	1.000000	S*, A*
1	30	Finafloxacin	45	Diluent Control	15	83.192904	1	7.442441E-20	83.192904	1	7.442441E-20	1.000000	S*, A*
1	20	Co-trimoxazole	45	Diluent Control	15	83.192904	1	7.442441E-20	83.192904	1	7.442441E-20	1.000000	S*, A*
1	21	Co-trimoxazole	45	Diluent Control	15	83.192904	1	7.442441E-20	83.192904	1	7.442441E-20	1.000000	S*, A*
1	22	Co-trimoxazole	45	Diluent Control	15	83.192904	1	7.442441E-20	83.192904	1	7.442441E-20	1.000000	S*, A*
1	23	Co-trimoxazole	45	Diluent Control	15	83.192904	1	7.442441E-20	83.192904	1	7.442441E-20	1.000000	S*, A*
1	24	Co-trimoxazole	45	Diluent Control	15	83.192904	1	7.442441E-20	83.192904	1	7.442441E-20	1.000000	S*, A*
1	25	Co-trimoxazole	45	Diluent Control	15	83.192904	1	7.442441E-20	83.192904	1	7.442441E-20	1.000000	S*, A*
1	26	Co-trimoxazole	45	Diluent Control	15	83.192904	1	7.442441E-20	83.192904	1	7.442441E-20	1.000000	S*, A*
1	27	Co-trimoxazole	45	Diluent Control	15	83.192904	1	7.442441E-20	83.192904	1	7.442441E-20	1.000000	S*, A*
1	28	Co-trimoxazole	45	Diluent Control	15	83.192904	1	7.442441E-20	83.192904	1	7.442441E-20	1.000000	S*, A*
1	29	Co-trimoxazole	45	Diluent Control	15	83.192904	1	7.442441E-20	83.192904	1	7.442441E-20	1.000000	S*, A*
1	30	Co-trimoxazole	45	Diluent Control	15	83.192904	1	7.442441E-20	83.192904	1	7.442441E-20	1.000000	S*, A*
1	20	Co-trimoxazole	45	Finafloxacin	45	1.764784	1	1.840289E-01	11.725428	1	6.165186E-04	298.496921	S*, A
1	21	Co-trimoxazole	45	Finafloxacin	45	4.005199	1	4.536014E-02	11.725428	1	6.165186E-04	73.574650	S*, A*
1	22	Co-trimoxazole	45	Finafloxacin	45	5.800621	1	1.602051E-02	11.725428	1	6.165186E-04	25.985450	S*, A*
1	23	Co-trimoxazole	45	Finafloxacin	45	9.455082	1	2.105649E-03	11.725428	1	6.165186E-04	3.415386	S*, A*
1	24	Co-trimoxazole	45	Finafloxacin	45	7.851441	1	5.078021E-03	11.725428	1	6.165186E-04	8.236607	S*, A*
1	25	Co-trimoxazole	45	Finafloxacin	45	7.409271	1	6.488862E-03	11.725428	1	6.165186E-04	10.525005	S*, A*
1	26	Co-trimoxazole	45	Finafloxacin	45	7.347731	1	6.714744E-03	11.725428	1	6.165186E-04	10.891388	S*, A*

Study	Weight Threshold	Comparison Group A		Comparison Group B		Alternative endpoint Comparison			Original study comparison			P-value ratio	P-value ratio change
		Treatment	n	Treatment	n	Test statistic	df	p-value	Test statistic	df	p-value		
1	27	Co-trimoxazole	45	Finafloxacin	45	9.624806	1	1.919667E-03	11.725428	1	6.165186E-04	3.113721	S*, A*
1	28	Co-trimoxazole	45	Finafloxacin	45	12.837690	1	3.397067E-04	11.725428	1	6.165186E-04	0.551008	S*, A*
1	29	Co-trimoxazole	45	Finafloxacin	45	12.038629	1	5.210929E-04	11.725428	1	6.165186E-04	0.845218	S*, A*
1	30	Co-trimoxazole	45	Finafloxacin	45	11.873783	1	5.692966E-04	11.725428	1	6.165186E-04	0.923405	S*, A*
2	20	Finafloxacin	90	Diluent Control	20	136.184833	1	1.817952E-31	136.184833	1	1.817952E-31	1.000000	S*, A*
2	21	Finafloxacin	90	Diluent Control	20	136.184833	1	1.817952E-31	136.184833	1	1.817952E-31	1.000000	S*, A*
2	22	Finafloxacin	90	Diluent Control	20	136.184833	1	1.817952E-31	136.184833	1	1.817952E-31	1.000000	S*, A*
2	23	Finafloxacin	90	Diluent Control	20	136.184833	1	1.817952E-31	136.184833	1	1.817952E-31	1.000000	S*, A*
2	24	Finafloxacin	90	Diluent Control	20	136.184833	1	1.817952E-31	136.184833	1	1.817952E-31	1.000000	S*, A*
2	25	Finafloxacin	90	Diluent Control	20	136.184833	1	1.817952E-31	136.184833	1	1.817952E-31	1.000000	S*, A*
2	26	Finafloxacin	90	Diluent Control	20	136.184833	1	1.817952E-31	136.184833	1	1.817952E-31	1.000000	S*, A*
2	27	Finafloxacin	90	Diluent Control	20	136.184833	1	1.817952E-31	136.184833	1	1.817952E-31	1.000000	S*, A*
2	28	Finafloxacin	90	Diluent Control	20	136.184833	1	1.817952E-31	136.184833	1	1.817952E-31	1.000000	S*, A*
2	29	Finafloxacin	90	Diluent Control	20	136.184833	1	1.817952E-31	136.184833	1	1.817952E-31	1.000000	S*, A*
2	30	Finafloxacin	90	Diluent Control	20	136.184833	1	1.817952E-31	136.184833	1	1.817952E-31	1.000000	S*, A*
3	20	Finafloxacin	40	Diluent Control	15	65.229390	1	6.666781E-16	65.229390	1	6.666781E-16	1.000000	S*, A*
3	21	Finafloxacin	40	Diluent Control	15	65.229390	1	6.666781E-16	65.229390	1	6.666781E-16	1.000000	S*, A*
3	22	Finafloxacin	40	Diluent Control	15	65.229390	1	6.666781E-16	65.229390	1	6.666781E-16	1.000000	S*, A*
3	23	Finafloxacin	40	Diluent Control	15	65.229390	1	6.666781E-16	65.229390	1	6.666781E-16	1.000000	S*, A*
3	24	Finafloxacin	40	Diluent Control	15	65.229390	1	6.666781E-16	65.229390	1	6.666781E-16	1.000000	S*, A*
3	25	Finafloxacin	40	Diluent Control	15	65.229390	1	6.666781E-16	65.229390	1	6.666781E-16	1.000000	S*, A*
3	26	Finafloxacin	40	Diluent Control	15	65.229390	1	6.666781E-16	65.229390	1	6.666781E-16	1.000000	S*, A*
3	27	Finafloxacin	40	Diluent Control	15	65.229390	1	6.666781E-16	65.229390	1	6.666781E-16	1.000000	S*, A*
3	28	Finafloxacin	40	Diluent Control	15	65.229390	1	6.666781E-16	65.229390	1	6.666781E-16	1.000000	S*, A*
3	29	Finafloxacin	40	Diluent Control	15	65.229390	1	6.666781E-16	65.229390	1	6.666781E-16	1.000000	S*, A*
3	30	Finafloxacin	40	Diluent Control	15	65.229390	1	6.666781E-16	65.229390	1	6.666781E-16	1.000000	S*, A*
3	20	Doxycycline	40	Diluent Control	15	65.229390	1	6.666781E-16	65.229390	1	6.666781E-16	1.000000	S*, A*
3	21	Doxycycline	40	Diluent Control	15	65.229390	1	6.666781E-16	65.229390	1	6.666781E-16	1.000000	S*, A*
3	22	Doxycycline	40	Diluent Control	15	65.229390	1	6.666781E-16	65.229390	1	6.666781E-16	1.000000	S*, A*
3	23	Doxycycline	40	Diluent Control	15	65.229390	1	6.666781E-16	65.229390	1	6.666781E-16	1.000000	S*, A*
3	24	Doxycycline	40	Diluent Control	15	65.229390	1	6.666781E-16	65.229390	1	6.666781E-16	1.000000	S*, A*
3	25	Doxycycline	40	Diluent Control	15	65.229390	1	6.666781E-16	65.229390	1	6.666781E-16	1.000000	S*, A*
3	26	Doxycycline	40	Diluent Control	15	65.229390	1	6.666781E-16	65.229390	1	6.666781E-16	1.000000	S*, A*
3	27	Doxycycline	40	Diluent Control	15	65.229390	1	6.666781E-16	65.229390	1	6.666781E-16	1.000000	S*, A*
3	28	Doxycycline	40	Diluent Control	15	65.229390	1	6.666781E-16	65.229390	1	6.666781E-16	1.000000	S*, A*
3	29	Doxycycline	40	Diluent Control	15	65.229390	1	6.666781E-16	65.229390	1	6.666781E-16	1.000000	S*, A*
3	30	Doxycycline	40	Diluent Control	15	65.229390	1	6.666781E-16	65.229390	1	6.666781E-16	1.000000	S*, A*
3	20	Doxycycline	40	Finafloxacin	40	6.377765	1	1.155589E-02	4.326465	1	3.752411E-02	0.307959	S*, A*
3	21	Doxycycline	40	Finafloxacin	40	5.112743	1	2.375075E-02	4.326465	1	3.752411E-02	0.632946	S*, A*

Study	Weight Threshold	Comparison Group A		Comparison Group B		Alternative endpoint Comparison			Original study comparison			P-value ratio	P-value ratio change
		Treatment	n	Treatment	n	Test statistic	df	p-value	Test statistic	df	p-value		
3	22	Doxycycline	40	Finafloxacin	40	5.111622	1	2.376609E-02	4.326465	1	3.752411E-02	0.633355	S*, A*
3	23	Doxycycline	40	Finafloxacin	40	3.475977	1	6.226596E-02	4.326465	1	3.752411E-02	1.659359	S*, A
3	24	Doxycycline	40	Finafloxacin	40	4.276748	1	3.863721E-02	4.326465	1	3.752411E-02	1.029664	S*, A*
3	25	Doxycycline	40	Finafloxacin	40	3.435375	1	6.381411E-02	4.326465	1	3.752411E-02	1.700616	S*, A
3	26	Doxycycline	40	Finafloxacin	40	2.565054	1	1.092488E-01	4.326465	1	3.752411E-02	2.911430	S*, A
3	27	Doxycycline	40	Finafloxacin	40	2.788552	1	9.493996E-02	4.326465	1	3.752411E-02	2.530106	S*, A
3	28	Doxycycline	40	Finafloxacin	40	4.326465	1	3.752411E-02	4.326465	1	3.752411E-02	1.000000	S*, A*
3	29	Doxycycline	40	Finafloxacin	40	4.326465	1	3.752411E-02	4.326465	1	3.752411E-02	1.000000	S*, A*
3	30	Doxycycline	40	Finafloxacin	40	4.326465	1	3.752411E-02	4.326465	1	3.752411E-02	1.000000	S*, A*
3	20	Finafloxacin & Doxycycline	40	Diluent Control	15	46.872846	1	7.574496E-12	46.872846	1	7.574496E-12	1.000000	S*, A*
3	21	Finafloxacin & Doxycycline	40	Diluent Control	15	46.872846	1	7.574496E-12	46.872846	1	7.574496E-12	1.000000	S*, A*
3	22	Finafloxacin & Doxycycline	40	Diluent Control	15	46.872846	1	7.574496E-12	46.872846	1	7.574496E-12	1.000000	S*, A*
3	23	Finafloxacin & Doxycycline	40	Diluent Control	15	46.872846	1	7.574496E-12	46.872846	1	7.574496E-12	1.000000	S*, A*
3	24	Finafloxacin & Doxycycline	40	Diluent Control	15	46.872846	1	7.574496E-12	46.872846	1	7.574496E-12	1.000000	S*, A*
3	25	Finafloxacin & Doxycycline	40	Diluent Control	15	46.872846	1	7.574496E-12	46.872846	1	7.574496E-12	1.000000	S*, A*
3	26	Finafloxacin & Doxycycline	40	Diluent Control	15	46.872846	1	7.574496E-12	46.872846	1	7.574496E-12	1.000000	S*, A*
3	27	Finafloxacin & Doxycycline	40	Diluent Control	15	46.872846	1	7.574496E-12	46.872846	1	7.574496E-12	1.000000	S*, A*
3	28	Finafloxacin & Doxycycline	40	Diluent Control	15	46.872846	1	7.574496E-12	46.872846	1	7.574496E-12	1.000000	S*, A*
3	29	Finafloxacin & Doxycycline	40	Diluent Control	15	46.872846	1	7.574496E-12	46.872846	1	7.574496E-12	1.000000	S*, A*
3	30	Finafloxacin & Doxycycline	40	Diluent Control	15	46.872846	1	7.574496E-12	46.872846	1	7.574496E-12	1.000000	S*, A*
3	20	Finafloxacin & Doxycycline	40	Finafloxacin	40	1.010082	1	3.148832E-01	0.709539	1	3.995968E-01	0.788002	S, A
3	21	Finafloxacin & Doxycycline	40	Finafloxacin	40	1.022745	1	3.118688E-01	0.709539	1	3.995968E-01	0.780459	S, A
3	22	Finafloxacin & Doxycycline	40	Finafloxacin	40	1.004097	1	3.163213E-01	0.709539	1	3.995968E-01	0.791601	S, A
3	23	Finafloxacin & Doxycycline	40	Finafloxacin	40	0.543344	1	4.610503E-01	0.709539	1	3.995968E-01	1.153789	S, A
3	24	Finafloxacin & Doxycycline	40	Finafloxacin	40	1.087494	1	2.970271E-01	0.709539	1	3.995968E-01	0.743317	S, A
3	25	Finafloxacin & Doxycycline	40	Finafloxacin	40	1.101193	1	2.940044E-01	0.709539	1	3.995968E-01	0.735753	S, A
3	26	Finafloxacin & Doxycycline	40	Finafloxacin	40	0.527210	1	4.677824E-01	0.709539	1	3.995968E-01	1.170636	S, A
3	27	Finafloxacin & Doxycycline	40	Finafloxacin	40	1.142105	1	2.852081E-01	0.709539	1	3.995968E-01	0.713740	S, A
3	28	Finafloxacin & Doxycycline	40	Finafloxacin	40	1.403252	1	2.361798E-01	0.709539	1	3.995968E-01	0.591045	S, A

Study	Weight Threshold	Comparison Group A		Comparison Group B		Alternative endpoint Comparison			Original study comparison			P-value ratio	P-value ratio change
		Treatment	n	Treatment	n	Test statistic	df	p-value	Test statistic	df	p-value		
3	29	Finafloxacin & Doxycycline	40	Finafloxacin	40	1.403252	1	2.361798E-01	0.709539	1	3.995968E-01	0.591045	S, A
3	30	Finafloxacin & Doxycycline	40	Finafloxacin	40	0.709539	1	3.995968E-01	0.709539	1	3.995968E-01	1.000000	S, A
3	20	Finafloxacin & Doxycycline	40	Doxycycline	40	2.194010	1	1.385482E-01	1.597411	1	2.062705E-01	0.671682	S, A
3	21	Finafloxacin & Doxycycline	40	Doxycycline	40	1.524480	1	2.169427E-01	1.597411	1	2.062705E-01	1.051739	S, A
3	22	Finafloxacin & Doxycycline	40	Doxycycline	40	1.507345	1	2.195447E-01	1.597411	1	2.062705E-01	1.064353	S, A
3	23	Finafloxacin & Doxycycline	40	Doxycycline	40	1.123972	1	2.890648E-01	1.597411	1	2.062705E-01	1.401387	S, A
3	24	Finafloxacin & Doxycycline	40	Doxycycline	40	1.077672	1	2.992189E-01	1.597411	1	2.062705E-01	1.450614	S, A
3	25	Finafloxacin & Doxycycline	40	Doxycycline	40	0.660460	1	4.163976E-01	1.597411	1	2.062705E-01	2.018697	S, A
3	26	Finafloxacin & Doxycycline	40	Doxycycline	40	0.768297	1	3.807445E-01	1.597411	1	2.062705E-01	1.845851	S, A
3	27	Finafloxacin & Doxycycline	40	Doxycycline	40	0.354552	1	5.515474E-01	1.597411	1	2.062705E-01	2.673904	S, A
3	28	Finafloxacin & Doxycycline	40	Doxycycline	40	0.832075	1	3.616731E-01	1.597411	1	2.062705E-01	1.753392	S, A
3	29	Finafloxacin & Doxycycline	40	Doxycycline	40	0.832075	1	3.616731E-01	1.597411	1	2.062705E-01	1.753392	S, A
3	30	Finafloxacin & Doxycycline	40	Doxycycline	40	1.597411	1	2.062705E-01	1.597411	1	2.062705E-01	1.000000	S, A
4	20	Finafloxacin 36 h	45	Finafloxacin 48 h	44	0.445966	1	5.042569E-01	1.730962	1	1.882881E-01	2.678114	S, A
4	21	Finafloxacin 36 h	45	Finafloxacin 48 h	44	0.235782	1	6.272695E-01	1.730962	1	1.882881E-01	3.331435	S, A
4	22	Finafloxacin 36 h	45	Finafloxacin 48 h	44	0.454321	1	5.002902E-01	1.730962	1	1.882881E-01	2.657047	S, A
4	23	Finafloxacin 36 h	45	Finafloxacin 48 h	44	0.211535	1	6.455672E-01	1.730962	1	1.882881E-01	3.428615	S, A
4	24	Finafloxacin 36 h	45	Finafloxacin 48 h	44	0.570395	1	4.501019E-01	1.730962	1	1.882881E-01	2.390496	S, A
4	25	Finafloxacin 36 h	45	Finafloxacin 48 h	44	0.960632	1	3.270278E-01	1.730962	1	1.882881E-01	1.736848	S, A
4	26	Finafloxacin 36 h	45	Finafloxacin 48 h	44	0.971426	1	3.243248E-01	1.730962	1	1.882881E-01	1.722493	S, A
4	27	Finafloxacin 36 h	45	Finafloxacin 48 h	44	0.949191	1	3.299252E-01	1.730962	1	1.882881E-01	1.752236	S, A
4	28	Finafloxacin 36 h	45	Finafloxacin 48 h	44	1.281959	1	2.575351E-01	1.730962	1	1.882881E-01	1.367772	S, A
4	29	Finafloxacin 36 h	45	Finafloxacin 48 h	44	1.785758	1	1.814439E-01	1.730962	1	1.882881E-01	0.963650	S, A
4	30	Finafloxacin 36 h	45	Finafloxacin 48 h	44	1.749705	1	1.859138E-01	1.730962	1	1.882881E-01	0.987390	S, A
4	20	Finafloxacin 36 h	45	Diluent Control	88	94.766888	1	2.141784E-22	94.766888	1	2.141784E-22	1.000000	S*, A*
4	21	Finafloxacin 36 h	45	Diluent Control	88	94.766888	1	2.141784E-22	94.766888	1	2.141784E-22	1.000000	S*, A*
4	22	Finafloxacin 36 h	45	Diluent Control	88	94.766888	1	2.141784E-22	94.766888	1	2.141784E-22	1.000000	S*, A*
4	23	Finafloxacin 36 h	45	Diluent Control	88	94.766888	1	2.141784E-22	94.766888	1	2.141784E-22	1.000000	S*, A*
4	24	Finafloxacin 36 h	45	Diluent Control	88	94.766888	1	2.141784E-22	94.766888	1	2.141784E-22	1.000000	S*, A*
4	25	Finafloxacin 36 h	45	Diluent Control	88	94.766888	1	2.141784E-22	94.766888	1	2.141784E-22	1.000000	S*, A*
4	26	Finafloxacin 36 h	45	Diluent Control	88	94.766888	1	2.141784E-22	94.766888	1	2.141784E-22	1.000000	S*, A*
4	27	Finafloxacin 36 h	45	Diluent Control	88	94.766888	1	2.141784E-22	94.766888	1	2.141784E-22	1.000000	S*, A*

Study	Weight Threshold	Comparison Group A		Comparison Group B		Alternative endpoint Comparison			Original study comparison			P-value ratio	P-value ratio change
		Treatment	n	Treatment	n	Test statistic	df	p-value	Test statistic	df	p-value		
4	28	Finafloxacin 36 h	45	Diluent Control	88	94.766888	1	2.141784E-22	94.766888	1	2.141784E-22	1.000000	S*, A*
4	29	Finafloxacin 36 h	45	Diluent Control	88	94.766888	1	2.141784E-22	94.766888	1	2.141784E-22	1.000000	S*, A*
4	30	Finafloxacin 36 h	45	Diluent Control	88	94.766888	1	2.141784E-22	94.766888	1	2.141784E-22	1.000000	S*, A*
4	20	Finafloxacin 48 h	44	Diluent Control	88	72.966845	1	1.318462E-17	72.966845	1	1.318462E-17	1.000000	S*, A*
4	21	Finafloxacin 48 h	44	Diluent Control	88	72.966845	1	1.318462E-17	72.966845	1	1.318462E-17	1.000000	S*, A*
4	22	Finafloxacin 48 h	44	Diluent Control	88	72.966845	1	1.318462E-17	72.966845	1	1.318462E-17	1.000000	S*, A*
4	23	Finafloxacin 48 h	44	Diluent Control	88	72.966845	1	1.318462E-17	72.966845	1	1.318462E-17	1.000000	S*, A*
4	24	Finafloxacin 48 h	44	Diluent Control	88	72.966845	1	1.318462E-17	72.966845	1	1.318462E-17	1.000000	S*, A*
4	25	Finafloxacin 48 h	44	Diluent Control	88	72.966845	1	1.318462E-17	72.966845	1	1.318462E-17	1.000000	S*, A*
4	26	Finafloxacin 48 h	44	Diluent Control	88	72.966845	1	1.318462E-17	72.966845	1	1.318462E-17	1.000000	S*, A*
4	27	Finafloxacin 48 h	44	Diluent Control	88	72.966845	1	1.318462E-17	72.966845	1	1.318462E-17	1.000000	S*, A*
4	28	Finafloxacin 48 h	44	Diluent Control	88	72.966845	1	1.318462E-17	72.966845	1	1.318462E-17	1.000000	S*, A*
4	29	Finafloxacin 48 h	44	Diluent Control	88	72.966845	1	1.318462E-17	72.966845	1	1.318462E-17	1.000000	S*, A*
4	30	Finafloxacin 48 h	44	Diluent Control	88	72.966845	1	1.318462E-17	72.966845	1	1.318462E-17	1.000000	S*, A*

n = number of mice, df = degrees of freedom, S* = Significant difference in original study comparison, A* = Significant difference in alternative comparison, S = No significant difference in original study comparison, A = No significant difference in alternative comparison.

Supplementary Table 2.

Logrank test results for the comparisons between treatment groups for each of the four studies, split by the signs threshold metric type, and signs threshold value (without weight loss thresholds applied). Information for the logrank test using the alternative endpoint comparison and orginal study comparison are presented, along with the p-value ratio and indicator of whether a change in significance resulted: (S*, A*) denotes instances where both the original study comparison (S) and the alternative comparison (A) were significant; (S, A) denotes instances where both the original study comparison and the alternative comparison were <u>not</u> significant; (S*, A) denotes instances where the original study comparison was significant but the alternative comparison was <u>not</u> significant; and (S, A*) denotes instances where the original study comparison was not significant but the alternative comparison was significant. These data were used for Figure A3.

Study	Threshold Metric	Signs threshold	Comparison Group A		Comparison Group B		Alternative endpoint Comparison			Original study comparison			P-value ratio	P-value ratio change
			Treatment	n	Treatment	n	Test statistic	df	p-value	Test statistic	df	p-value		
1	Total signs	≥4	Finafloxacin	45	Diluent Control	15	83.192904	1	7.44244E-20	83.192904	1	7.44244E-20	1.000000	S*, A*
1	Average signs	≥4	Finafloxacin	45	Diluent Control	15	83.192904	1	7.44244E-20	83.192904	1	7.44244E-20	1.000000	S*, A*
1	Total signs	≥4	Co-trimoxazole	45	Diluent Control	15	83.192904	1	7.44244E-20	83.192904	1	7.44244E-20	1.000000	S*, A*
1	Average signs	≥4	Co-trimoxazole	45	Diluent Control	15	83.192904	1	7.44244E-20	83.192904	1	7.44244E-20	1.000000	S*, A*
1	Total signs	≥4	Co-trimoxazole	45	Finafloxacin	45	18.200148	1	1.98863E-05	11.725428	1	6.16519E-04	0.032256	S*, A*
1	Average signs	≥4	Co-trimoxazole	45	Finafloxacin	45	16.452245	1	4.98911E-05	11.725428	1	6.16519E-04	0.080924	S*, A*
2	Total signs	≥4	Finafloxacin	90	Diluent Control	20	136.184833	1	1.81795E-31	136.184833	1	1.81795E-31	1.000000	S*, A*

Study	Threshold Metric	Signs threshold	Comparison Group A		Comparison Group B		Alternative endpoint Comparison			Original study comparison			P-value ratio	P-value ratio change
			Treatment	n	Treatment	n	Test statistic	df	p-value	Test statistic	df	p-value		
2	Average signs	≥4	Finafloxacin	90	Diluent Control	20	136.184833	1	1.81795E-31	136.184833	1	1.81795E-31	1.000000	S*, A*
3	Total signs	≥4	Finafloxacin	40	Diluent Control	15	65.229390	1	6.66678E-16	65.229390	1	6.66678E-16	1.000000	S*, A*
3	Average signs	≥4	Finafloxacin	40	Diluent Control	15	65.229390	1	6.66678E-16	65.229390	1	6.66678E-16	1.000000	S*, A*
3	Total signs	≥4	Doxycycline	40	Diluent Control	15	65.229390	1	6.66678E-16	65.229390	1	6.66678E-16	1.000000	S*, A*
3	Average signs	≥4	Doxycycline	40	Diluent Control	15	65.229390	1	6.66678E-16	65.229390	1	6.66678E-16	1.000000	S*, A*
3	Total signs	≥4	Doxycycline	40	Finafloxacin	40	4.668787	1	3.07156E-02	4.326465	1	3.75241E-02	0.818557	S*, A*
3	Average signs	≥4	Doxycycline	40	Finafloxacin	40	8.717642	1	3.15145E-03	4.326465	1	3.75241E-02	0.083985	S*, A*
3	Total signs	≥4	Finafloxacin & Doxycycline	40	Diluent Control	15	46.872846	1	7.57450E-12	46.872846	1	7.57450E-12	1.000000	S*, A*
3	Average signs	≥4	Finafloxacin & Doxycycline	40	Diluent Control	15	46.872846	1	7.57450E-12	46.872846	1	7.57450E-12	1.000000	S*, A*
3	Total signs	≥4	Finafloxacin & Doxycycline	40	Finafloxacin	40	1.299280	1	2.54345E-01	0.709539	1	3.99597E-01	0.636504	S, A
3	Average signs	≥4	Finafloxacin & Doxycycline	40	Finafloxacin	40	0.709539	1	3.99597E-01	0.709539	1	3.99597E-01	1.000000	S, A
3	Total signs	≥4	Finafloxacin & Doxycycline	40	Doxycycline	40	9.071851	1	2.59574E-03	1.597411	1	2.06270E-01	0.012584	S, A*
3	Average signs	≥4	Finafloxacin & Doxycycline	40	Doxycycline	40	4.914739	1	2.66285E-02	1.597411	1	2.06270E-01	0.129095	S, A*
4	Total signs	≥4	Finafloxacin 36 h	45	Finafloxacin 48 h	44	0.515316	1	4.72847E-01	1.730962	1	1.88288E-01	2.511293	S, A
4	Average signs	≥4	Finafloxacin 36 h	45	Finafloxacin 48 h	44	0.307025	1	5.79512E-01	1.730962	1	1.88288E-01	3.077794	S, A
4	Total signs	≥4	Finafloxacin 36 h	45	Diluent Control	88	94.766888	1	2.14178E-22	94.766888	1	2.14178E-22	1.000000	S*, A*
4	Average signs	≥4	Finafloxacin 36 h	45	Diluent Control	88	94.766888	1	2.14178E-22	94.766888	1	2.14178E-22	1.000000	S*, A*
4	Total signs	≥4	Finafloxacin 48 h	44	Diluent Control	88	72.966845	1	1.31846E-17	72.966845	1	1.31846E-17	1.000000	S*, A*
4	Average signs	≥4	Finafloxacin 48 h	44	Diluent Control	88	72.966845	1	1.31846E-17	72.966845	1	1.31846E-17	1.000000	S*, A*
1	Total signs	≥5	Finafloxacin	45	Diluent Control	15	83.192904	1	7.44244E-20	83.192904	1	7.44244E-20	1.000000	S*, A*
1	Average signs	≥5	Finafloxacin	45	Diluent Control	15	83.192904	1	7.44244E-20	83.192904	1	7.44244E-20	1.000000	S*, A*
1	Total signs	≥5	Co-trimoxazole	45	Diluent Control	15	83.192904	1	7.44244E-20	83.192904	1	7.44244E-20	1.000000	S*, A*
1	Average signs	≥5	Co-trimoxazole	45	Diluent Control	15	83.192904	1	7.44244E-20	83.192904	1	7.44244E-20	1.000000	S*, A*
1	Total signs	≥5	Co-trimoxazole	45	Finafloxacin	45	18.495978	1	1.70263E-05	11.725428	1	6.16519E-04	0.027617	S*, A*
1	Average signs	≥5	Co-trimoxazole	45	Finafloxacin	45	17.583146	1	2.75015E-05	11.725428	1	6.16519E-04	0.044608	S*, A*
2	Total signs	≥5	Finafloxacin	90	Diluent Control	20	136.184833	1	1.81795E-31	136.184833	1	1.81795E-31	1.000000	S*, A*
2	Average signs	≥5	Finafloxacin	90	Diluent Control	20	136.184833	1	1.81795E-31	136.184833	1	1.81795E-31	1.000000	S*, A*
3	Total signs	≥5	Finafloxacin	40	Diluent Control	15	65.229390	1	6.66678E-16	65.229390	1	6.66678E-16	1.000000	S*, A*
3	Average signs	≥5	Finafloxacin	40	Diluent Control	15	65.229390	1	6.66678E-16	65.229390	1	6.66678E-16	1.000000	S*, A*
3	Total signs	≥5	Doxycycline	40	Diluent Control	15	65.229390	1	6.66678E-16	65.229390	1	6.66678E-16	1.000000	S*, A*
3	Average signs	≥5	Doxycycline	40	Diluent Control	15	65.229390	1	6.66678E-16	65.229390	1	6.66678E-16	1.000000	S*, A*
3	Total signs	≥5	Doxycycline	40	Finafloxacin	40	7.230527	1	7.16742E-03	4.326465	1	3.75241E-02	0.191008	S*, A*
3	Average signs	≥5	Doxycycline	40	Finafloxacin	40	5.521333	1	1.87859E-02	4.326465	1	3.75241E-02	0.500637	S*, A*
3	Total signs	≥5	Finafloxacin & Doxycycline	40	Diluent Control	15	46.872846	1	7.57450E-12	46.872846	1	7.57450E-12	1.000000	S*, A*
3	Average signs	≥5	Finafloxacin & Doxycycline	40	Diluent Control	15	46.872846	1	7.57450E-12	46.872846	1	7.57450E-12	1.000000	S*, A*

Study	Threshold Metric	Signs threshold	Comparison Group A		Comparison Group B		Alternative endpoint Comparison			Original study comparison			P-value ratio	P-value ratio change
			Treatment	n	Treatment	n	Test statistic	df	p-value	Test statistic	df	p-value		
3	Total signs	≥5	Finafloxacin & Doxycycline	40	Finafloxacin	40	0.161476	1	6.87801E-01	0.709539	1	3.99597E-01	1.721238	S, A
3	Average signs	≥5	Finafloxacin & Doxycycline	40	Finafloxacin	40	0.709539	1	3.99597E-01	0.709539	1	3.99597E-01	1.000000	S, A
3	Total signs	≥5	Finafloxacin & Doxycycline	40	Doxycycline	40	5.440163	1	1.96788E-02	1.597411	1	2.06270E-01	0.095403	S, A*
3	Average signs	≥5	Finafloxacin & Doxycycline	40	Doxycycline	40	2.562879	1	1.09399E-01	1.597411	1	2.06270E-01	0.530368	S, A
4	Total signs	≥5	Finafloxacin 36 h	45	Finafloxacin 48 h	44	0.335219	1	5.62602E-01	1.730962	1	1.88288E-01	2.987986	S, A
4	Average signs	≥5	Finafloxacin 36 h	45	Finafloxacin 48 h	44	1.792966	1	1.80565E-01	1.730962	1	1.88288E-01	0.958984	S, A
4	Total signs	≥5	Finafloxacin 36 h	45	Diluent Control	88	94.766888	1	2.14178E-22	94.766888	1	2.14178E-22	1.000000	S*, A*
4	Average signs	≥5	Finafloxacin 36 h	45	Diluent Control	88	94.766888	1	2.14178E-22	94.766888	1	2.14178E-22	1.000000	S*, A*
4	Total signs	≥5	Finafloxacin 48 h	44	Diluent Control	88	72.966845	1	1.31846E-17	72.966845	1	1.31846E-17	1.000000	S*, A*
4	Average signs	≥5	Finafloxacin 48 h	44	Diluent Control	88	72.966845	1	1.31846E-17	72.966845	1	1.31846E-17	1.000000	S*, A*
1	Total signs	≥6	Finafloxacin	45	Diluent Control	15	83.192904	1	7.44244E-20	83.192904	1	7.44244E-20	1.000000	S*, A*
1	Average signs	≥6	Finafloxacin	45	Diluent Control	15	83.192904	1	7.44244E-20	83.192904	1	7.44244E-20	1.000000	S*, A*
1	Total signs	≥6	Co-trimoxazole	45	Diluent Control	15	83.192904	1	7.44244E-20	83.192904	1	7.44244E-20	1.000000	S*, A*
1	Average signs	≥6	Co-trimoxazole	45	Diluent Control	15	83.192904	1	7.44244E-20	83.192904	1	7.44244E-20	1.000000	S*, A*
1	Total signs	≥6	Co-trimoxazole	45	Finafloxacin	45	15.099274	1	1.02003E-04	11.725428	1	6.16519E-04	0.165449	S*, A*
1	Average signs	≥6	Co-trimoxazole	45	Finafloxacin	45	15.885905	1	6.72779E-05	11.725428	1	6.16519E-04	0.109125	S*, A*
2	Total signs	≥6	Finafloxacin	90	Diluent Control	20	136.184833	1	1.81795E-31	136.184833	1	1.81795E-31	1.000000	S*, A*
2	Average signs	≥6	Finafloxacin	90	Diluent Control	20	136.184833	1	1.81795E-31	136.184833	1	1.81795E-31	1.000000	S*, A*
3	Total signs	≥6	Finafloxacin	40	Diluent Control	15	65.229390	1	6.66678E-16	65.229390	1	6.66678E-16	1.000000	S*, A*
3	Average signs	≥6	Finafloxacin	40	Diluent Control	15	65.229390	1	6.66678E-16	65.229390	1	6.66678E-16	1.000000	S*, A*
3	Total signs	≥6	Doxycycline	40	Diluent Control	15	65.229390	1	6.66678E-16	65.229390	1	6.66678E-16	1.000000	S*, A*
3	Average signs	≥6	Doxycycline	40	Diluent Control	15	65.229390	1	6.66678E-16	65.229390	1	6.66678E-16	1.000000	S*, A*
3	Total signs	≥6	Doxycycline	40	Finafloxacin	40	4.326465	1	3.75241E-02	4.326465	1	3.75241E-02	1.000000	S*, A*
3	Average signs	≥6	Doxycycline	40	Finafloxacin	40	4.326465	1	3.75241E-02	4.326465	1	3.75241E-02	1.000000	S*, A*
3	Total signs	≥6	Finafloxacin & Doxycycline	40	Diluent Control	15	46.872846	1	7.57450E-12	46.872846	1	7.57450E-12	1.000000	S*, A*
3	Average signs	≥6	Finafloxacin & Doxycycline	40	Diluent Control	15	46.872846	1	7.57450E-12	46.872846	1	7.57450E-12	1.000000	S*, A*
3	Total signs	≥6	Finafloxacin & Doxycycline	40	Finafloxacin	40	0.709539	1	3.99597E-01	0.709539	1	3.99597E-01	1.000000	S, A
3	Average signs	≥6	Finafloxacin & Doxycycline	40	Finafloxacin	40	0.709539	1	3.99597E-01	0.709539	1	3.99597E-01	1.000000	S, A
3	Total signs	≥6	Finafloxacin & Doxycycline	40	Doxycycline	40	1.597411	1	2.06270E-01	1.597411	1	2.06270E-01	1.000000	S, A
3	Average signs	≥6	Finafloxacin & Doxycycline	40	Doxycycline	40	1.597411	1	2.06270E-01	1.597411	1	2.06270E-01	1.000000	S, A
4	Total signs	≥6	Finafloxacin 36 h	45	Finafloxacin 48 h	44	1.237847	1	2.65886E-01	1.730962	1	1.88288E-01	1.412126	S, A
4	Average signs	≥6	Finafloxacin 36 h	45	Finafloxacin 48 h	44	1.730962	1	1.88288E-01	1.730962	1	1.88288E-01	1.000000	S, A
4	Total signs	≥6	Finafloxacin 36 h	45	Diluent Control	88	94.766888	1	2.14178E-22	94.766888	1	2.14178E-22	1.000000	S*, A*
4	Average signs	≥6	Finafloxacin 36 h	45	Diluent Control	88	94.766888	1	2.14178E-22	94.766888	1	2.14178E-22	1.000000	S*, A*

Study	Threshold Metric	Signs threshold	Comparison Group A		Comparison Group B		Alternative endpoint Comparison			Original study comparison			P-value ratio	P-value ratio change
			Treatment	n	Treatment	n	Test statistic	df	p-value	Test statistic	df	p-value		
4	Total signs	≥6	Finafloxacin 48 h	44	Diluent Control	88	72.966845	1	1.31846E-17	72.966845	1	1.31846E-17	1.000000	S*, A*
4	Average signs	≥6	Finafloxacin 48 h	44	Diluent Control	88	72.966845	1	1.31846E-17	72.966845	1	1.31846E-17	1.000000	S*, A*

n = number of mice, df = degrees of freedom, S* = Significant difference in original study comparison, A* = Significant difference in alternative comparison, S = No significant difference in original study comparison, A = No significant difference in alternative comparison.

Supplementary Table 3.

Logrank test results for the comparisons between treatment groups for each of the four studies, split by the signs threshold metric type, signs threshold value and weight threshold value. Information for the logrank test using the alternative endpoint comparison and orginal study comparison are presented, along with the p-value ratio and indicator of whether a change in significance resulted: (S*, A*) denotes instances where both the original study comparison (S) and the alternative comparison (A) were significant; (S, A) denotes instances where both the original study comparison and the alternative comparison were not significant; (S*, A) denotes instances where the original study comparison was significant but the alternative comparison was not significant; and (S, A*) denotes instances where the original study comparison was not significant but the alternative comparison was significant. These data were used for Figure 3 and Figure A4.

Study	Threshold Metric	Signs threshold	Weight threshold	Comparison Group A		Comparison Group B		Alternative endpoint Comparison			Original study comparison			P-value ratio	P-value ratio change
				Treatment	n	Treatment	n	Test statistic	df	p-value	Test statistic	df	p-value		
1	Total signs	≥4	20	Finafloxacin	45	Diluent Control	15	83.192904	1	7.442441E-20	83.192904	1	7.442441E-20	1.000000	S*, A*
1	Average signs	≥4	20	Finafloxacin	45	Diluent Control	15	83.192904	1	7.442441E-20	83.192904	1	7.442441E-20	1.000000	S*, A*
1	Total signs	≥4	21	Finafloxacin	45	Diluent Control	15	83.192904	1	7.442441E-20	83.192904	1	7.442441E-20	1.000000	S*, A*
1	Average signs	≥4	21	Finafloxacin	45	Diluent Control	15	83.192904	1	7.442441E-20	83.192904	1	7.442441E-20	1.000000	S*, A*
1	Total signs	≥4	22	Finafloxacin	45	Diluent Control	15	83.192904	1	7.442441E-20	83.192904	1	7.442441E-20	1.000000	S*, A*
1	Average signs	≥4	22	Finafloxacin	45	Diluent Control	15	83.192904	1	7.442441E-20	83.192904	1	7.442441E-20	1.000000	S*, A*
1	Total signs	≥4	23	Finafloxacin	45	Diluent Control	15	83.192904	1	7.442441E-20	83.192904	1	7.442441E-20	1.000000	S*, A*
1	Average signs	≥4	23	Finafloxacin	45	Diluent Control	15	83.192904	1	7.442441E-20	83.192904	1	7.442441E-20	1.000000	S*, A*
1	Total signs	≥4	24	Finafloxacin	45	Diluent Control	15	83.192904	1	7.442441E-20	83.192904	1	7.442441E-20	1.000000	S*, A*
1	Average signs	≥4	24	Finafloxacin	45	Diluent Control	15	83.192904	1	7.442441E-20	83.192904	1	7.442441E-20	1.000000	S*, A*
1	Total signs	≥4	25	Finafloxacin	45	Diluent Control	15	83.192904	1	7.442441E-20	83.192904	1	7.442441E-20	1.000000	S*, A*
1	Average signs	≥4	25	Finafloxacin	45	Diluent Control	15	83.192904	1	7.442441E-20	83.192904	1	7.442441E-20	1.000000	S*, A*
1	Total signs	≥4	26	Finafloxacin	45	Diluent Control	15	83.192904	1	7.442441E-20	83.192904	1	7.442441E-20	1.000000	S*, A*
1	Average signs	≥4	26	Finafloxacin	45	Diluent Control	15	83.192904	1	7.442441E-20	83.192904	1	7.442441E-20	1.000000	S*, A*
1	Total signs	≥4	27	Finafloxacin	45	Diluent Control	15	83.192904	1	7.442441E-20	83.192904	1	7.442441E-20	1.000000	S*, A*
1	Average signs	≥4	27	Finafloxacin	45	Diluent Control	15	83.192904	1	7.442441E-20	83.192904	1	7.442441E-20	1.000000	S*, A*
1	Total signs	≥4	28	Finafloxacin	45	Diluent Control	15	83.192904	1	7.442441E-20	83.192904	1	7.442441E-20	1.000000	S*, A*
1	Average signs	≥4	28	Finafloxacin	45	Diluent Control	15	83.192904	1	7.442441E-20	83.192904	1	7.442441E-20	1.000000	S*, A*
1	Total signs	≥4	29	Finafloxacin	45	Diluent Control	15	83.192904	1	7.442441E-20	83.192904	1	7.442441E-20	1.000000	S*, A*
1	Average signs	≥4	29	Finafloxacin	45	Diluent Control	15	83.192904	1	7.442441E-20	83.192904	1	7.442441E-20	1.000000	S*, A*
1	Total signs	≥4	30	Finafloxacin	45	Diluent Control	15	83.192904	1	7.442441E-20	83.192904	1	7.442441E-20	1.000000	S*, A*

Study	Threshold Metric	Signs threshold	Weight threshold	Comparison Group A		Comparison Group B		Alternative endpoint Comparison			Original study comparison			P-value ratio	P-value ratio change
				Treatment	n	Treatment	n	Test statistic	df	p-value	Test statistic	df	p-value		
1	Average signs	≥4	30	Finafloxacin	45	Diluent Control	15	83.192904	1	7.442441E-20	83.192904	1	7.442441E-20	1.000000	S*, A*
1	Total signs	≥4	20	Co-trimoxazole	45	Diluent Control	15	83.192904	1	7.442441E-20	83.192904	1	7.442441E-20	1.000000	S*, A*
1	Average signs	≥4	20	Co-trimoxazole	45	Diluent Control	15	83.192904	1	7.442441E-20	83.192904	1	7.442441E-20	1.000000	S*, A*
1	Total signs	≥4	21	Co-trimoxazole	45	Diluent Control	15	83.192904	1	7.442441E-20	83.192904	1	7.442441E-20	1.000000	S*, A*
1	Average signs	≥4	21	Co-trimoxazole	45	Diluent Control	15	83.192904	1	7.442441E-20	83.192904	1	7.442441E-20	1.000000	S*, A*
1	Total signs	≥4	22	Co-trimoxazole	45	Diluent Control	15	83.192904	1	7.442441E-20	83.192904	1	7.442441E-20	1.000000	S*, A*
1	Average signs	≥4	22	Co-trimoxazole	45	Diluent Control	15	83.192904	1	7.442441E-20	83.192904	1	7.442441E-20	1.000000	S*, A*
1	Total signs	≥4	23	Co-trimoxazole	45	Diluent Control	15	83.192904	1	7.442441E-20	83.192904	1	7.442441E-20	1.000000	S*, A*
1	Average signs	≥4	23	Co-trimoxazole	45	Diluent Control	15	83.192904	1	7.442441E-20	83.192904	1	7.442441E-20	1.000000	S*, A*
1	Total signs	≥4	24	Co-trimoxazole	45	Diluent Control	15	83.192904	1	7.442441E-20	83.192904	1	7.442441E-20	1.000000	S*, A*
1	Average signs	≥4	24	Co-trimoxazole	45	Diluent Control	15	83.192904	1	7.442441E-20	83.192904	1	7.442441E-20	1.000000	S*, A*
1	Total signs	≥4	25	Co-trimoxazole	45	Diluent Control	15	83.192904	1	7.442441E-20	83.192904	1	7.442441E-20	1.000000	S*, A*
1	Average signs	≥4	25	Co-trimoxazole	45	Diluent Control	15	83.192904	1	7.442441E-20	83.192904	1	7.442441E-20	1.000000	S*, A*
1	Total signs	≥4	26	Co-trimoxazole	45	Diluent Control	15	83.192904	1	7.442441E-20	83.192904	1	7.442441E-20	1.000000	S*, A*
1	Average signs	≥4	26	Co-trimoxazole	45	Diluent Control	15	83.192904	1	7.442441E-20	83.192904	1	7.442441E-20	1.000000	S*, A*
1	Total signs	≥4	27	Co-trimoxazole	45	Diluent Control	15	83.192904	1	7.442441E-20	83.192904	1	7.442441E-20	1.000000	S*, A*
1	Average signs	≥4	27	Co-trimoxazole	45	Diluent Control	15	83.192904	1	7.442441E-20	83.192904	1	7.442441E-20	1.000000	S*, A*
1	Total signs	≥4	28	Co-trimoxazole	45	Diluent Control	15	83.192904	1	7.442441E-20	83.192904	1	7.442441E-20	1.000000	S*, A*
1	Average signs	≥4	28	Co-trimoxazole	45	Diluent Control	15	83.192904	1	7.442441E-20	83.192904	1	7.442441E-20	1.000000	S*, A*
1	Total signs	≥4	29	Co-trimoxazole	45	Diluent Control	15	83.192904	1	7.442441E-20	83.192904	1	7.442441E-20	1.000000	S*, A*
1	Average signs	≥4	29	Co-trimoxazole	45	Diluent Control	15	83.192904	1	7.442441E-20	83.192904	1	7.442441E-20	1.000000	S*, A*
1	Total signs	≥4	30	Co-trimoxazole	45	Diluent Control	15	83.192904	1	7.442441E-20	83.192904	1	7.442441E-20	1.000000	S*, A*
1	Average signs	≥4	30	Co-trimoxazole	45	Diluent Control	15	83.192904	1	7.442441E-20	83.192904	1	7.442441E-20	1.000000	S*, A*
1	Total signs	≥4	20	Co-trimoxazole	45	Finafloxacin	45	2.961731	1	8.525661E-02	11.725428	1	6.165186E-04	138.287152	S*, A
1	Average signs	≥4	20	Co-trimoxazole	45	Finafloxacin	45	2.251945	1	1.334466E-01	11.725428	1	6.165186E-04	216.451820	S*, A
1	Total signs	≥4	21	Co-trimoxazole	45	Finafloxacin	45	5.401591	1	2.011841E-02	11.725428	1	6.165186E-04	32.632279	S*, A*
1	Average signs	≥4	21	Co-trimoxazole	45	Finafloxacin	45	4.494211	1	3.400980E-02	11.725428	1	6.165186E-04	55.164267	S*, A*
1	Total signs	≥4	22	Co-trimoxazole	45	Finafloxacin	45	8.095190	1	4.438288E-03	11.725428	1	6.165186E-04	7.198952	S*, A*
1	Average signs	≥4	22	Co-trimoxazole	45	Finafloxacin	45	6.851295	1	8.857705E-03	11.725428	1	6.165186E-04	14.367295	S*, A*
1	Total signs	≥4	23	Co-trimoxazole	45	Finafloxacin	45	12.456111	1	4.166264E-04	11.725428	1	6.165186E-04	0.675773	S*, A*
1	Average signs	≥4	23	Co-trimoxazole	45	Finafloxacin	45	11.208859	1	8.140774E-04	11.725428	1	6.165186E-04	1.320443	S*, A*
1	Total signs	≥4	24	Co-trimoxazole	45	Finafloxacin	45	12.534257	1	3.995584E-04	11.725428	1	6.165186E-04	0.648088	S*, A*
1	Average signs	≥4	24	Co-trimoxazole	45	Finafloxacin	45	11.352490	1	7.534688E-04	11.725428	1	6.165186E-04	1.222135	S*, A*
1	Total signs	≥4	25	Co-trimoxazole	45	Finafloxacin	45	12.480063	1	4.113183E-04	11.725428	1	6.165186E-04	0.667163	S*, A*
1	Average signs	≥4	25	Co-trimoxazole	45	Finafloxacin	45	11.270750	1	7.873791E-04	11.725428	1	6.165186E-04	1.277138	S*, A*
1	Total signs	≥4	26	Co-trimoxazole	45	Finafloxacin	45	12.551639	1	3.958589E-04	11.725428	1	6.165186E-04	0.642087	S*, A*
1	Average signs	≥4	26	Co-trimoxazole	45	Finafloxacin	45	11.447003	1	7.160940E-04	11.725428	1	6.165186E-04	1.161512	S*, A*
1	Total signs	≥4	27	Co-trimoxazole	45	Finafloxacin	45	15.826109	1	6.943772E-05	11.725428	1	6.165186E-04	0.112629	S*, A*

Study	Threshold Metric	Signs threshold	Weight threshold	Comparison Group A		Comparison Group B		Alternative endpoint Comparison			Original study comparison			P-value ratio	P-value ratio change
				Treatment	n	Treatment	n	Test statistic	df	p-value	Test statistic	df	p-value		
1	Average signs	≥4	27	Co-trimoxazole	45	Finafloxacin	45	14.125323	1	1.710258E-04	11.725428	1	6.165186E-04	0.277406	S*, A*
1	Total signs	≥4	28	Co-trimoxazole	45	Finafloxacin	45	17.766153	1	2.497877E-05	11.725428	1	6.165186E-04	0.040516	S*, A*
1	Average signs	≥4	28	Co-trimoxazole	45	Finafloxacin	45	16.144333	1	5.869390E-05	11.725428	1	6.165186E-04	0.095202	S*, A*
1	Total signs	≥4	29	Co-trimoxazole	45	Finafloxacin	45	18.032521	1	2.171633E-05	11.725428	1	6.165186E-04	0.035224	S*, A*
1	Average signs	≥4	29	Co-trimoxazole	45	Finafloxacin	45	16.265772	1	5.504945E-05	11.725428	1	6.165186E-04	0.089291	S*, A*
1	Total signs	≥4	30	Co-trimoxazole	45	Finafloxacin	45	18.032521	1	2.171633E-05	11.725428	1	6.165186E-04	0.035224	S*, A*
1	Average signs	≥4	30	Co-trimoxazole	45	Finafloxacin	45	16.309031	1	5.380689E-05	11.725428	1	6.165186E-04	0.087275	S*, A*
2	Total signs	≥4	20	Finafloxacin	90	Diluent Control	20	136.184833	1	1.817952E-31	136.184833	1	1.817952E-31	1.000000	S*, A*
2	Average signs	≥4	20	Finafloxacin	90	Diluent Control	20	136.184833	1	1.817952E-31	136.184833	1	1.817952E-31	1.000000	S*, A*
2	Total signs	≥4	21	Finafloxacin	90	Diluent Control	20	136.184833	1	1.817952E-31	136.184833	1	1.817952E-31	1.000000	S*, A*
2	Average signs	≥4	21	Finafloxacin	90	Diluent Control	20	136.184833	1	1.817952E-31	136.184833	1	1.817952E-31	1.000000	S*, A*
2	Total signs	≥4	22	Finafloxacin	90	Diluent Control	20	136.184833	1	1.817952E-31	136.184833	1	1.817952E-31	1.000000	S*, A*
2	Average signs	≥4	22	Finafloxacin	90	Diluent Control	20	136.184833	1	1.817952E-31	136.184833	1	1.817952E-31	1.000000	S*, A*
2	Total signs	≥4	23	Finafloxacin	90	Diluent Control	20	136.184833	1	1.817952E-31	136.184833	1	1.817952E-31	1.000000	S*, A*
2	Average signs	≥4	23	Finafloxacin	90	Diluent Control	20	136.184833	1	1.817952E-31	136.184833	1	1.817952E-31	1.000000	S*, A*
2	Total signs	≥4	24	Finafloxacin	90	Diluent Control	20	136.184833	1	1.817952E-31	136.184833	1	1.817952E-31	1.000000	S*, A*
2	Average signs	≥4	24	Finafloxacin	90	Diluent Control	20	136.184833	1	1.817952E-31	136.184833	1	1.817952E-31	1.000000	S*, A*
2	Total signs	≥4	25	Finafloxacin	90	Diluent Control	20	136.184833	1	1.817952E-31	136.184833	1	1.817952E-31	1.000000	S*, A*
2	Average signs	≥4	25	Finafloxacin	90	Diluent Control	20	136.184833	1	1.817952E-31	136.184833	1	1.817952E-31	1.000000	S*, A*
2	Total signs	≥4	26	Finafloxacin	90	Diluent Control	20	136.184833	1	1.817952E-31	136.184833	1	1.817952E-31	1.000000	S*, A*
2	Average signs	≥4	26	Finafloxacin	90	Diluent Control	20	136.184833	1	1.817952E-31	136.184833	1	1.817952E-31	1.000000	S*, A*
2	Total signs	≥4	27	Finafloxacin	90	Diluent Control	20	136.184833	1	1.817952E-31	136.184833	1	1.817952E-31	1.000000	S*, A*
2	Average signs	≥4	27	Finafloxacin	90	Diluent Control	20	136.184833	1	1.817952E-31	136.184833	1	1.817952E-31	1.000000	S*, A*
2	Total signs	≥4	28	Finafloxacin	90	Diluent Control	20	136.184833	1	1.817952E-31	136.184833	1	1.817952E-31	1.000000	S*, A*
2	Average signs	≥4	28	Finafloxacin	90	Diluent Control	20	136.184833	1	1.817952E-31	136.184833	1	1.817952E-31	1.000000	S*, A*
2	Total signs	≥4	29	Finafloxacin	90	Diluent Control	20	136.184833	1	1.817952E-31	136.184833	1	1.817952E-31	1.000000	S*, A*
2	Average signs	≥4	29	Finafloxacin	90	Diluent Control	20	136.184833	1	1.817952E-31	136.184833	1	1.817952E-31	1.000000	S*, A*
2	Total signs	≥4	30	Finafloxacin	90	Diluent Control	20	136.184833	1	1.817952E-31	136.184833	1	1.817952E-31	1.000000	S*, A*
2	Average signs	≥4	30	Finafloxacin	90	Diluent Control	20	136.184833	1	1.817952E-31	136.184833	1	1.817952E-31	1.000000	S*, A*
3	Total signs	≥4	20	Finafloxacin	40	Diluent Control	15	65.229390	1	6.666781E-16	65.229390	1	6.666781E-16	1.000000	S*, A*
3	Average signs	≥4	20	Finafloxacin	40	Diluent Control	15	65.229390	1	6.666781E-16	65.229390	1	6.666781E-16	1.000000	S*, A*
3	Total signs	≥4	21	Finafloxacin	40	Diluent Control	15	65.229390	1	6.666781E-16	65.229390	1	6.666781E-16	1.000000	S*, A*
3	Average signs	≥4	21	Finafloxacin	40	Diluent Control	15	65.229390	1	6.666781E-16	65.229390	1	6.666781E-16	1.000000	S*, A*
3	Total signs	≥4	22	Finafloxacin	40	Diluent Control	15	65.229390	1	6.666781E-16	65.229390	1	6.666781E-16	1.000000	S*, A*
3	Average signs	≥4	22	Finafloxacin	40	Diluent Control	15	65.229390	1	6.666781E-16	65.229390	1	6.666781E-16	1.000000	S*, A*
3	Total signs	≥4	23	Finafloxacin	40	Diluent Control	15	65.229390	1	6.666781E-16	65.229390	1	6.666781E-16	1.000000	S*, A*
3	Average signs	≥4	23	Finafloxacin	40	Diluent Control	15	65.229390	1	6.666781E-16	65.229390	1	6.666781E-16	1.000000	S*, A*
3	Total signs	≥4	24	Finafloxacin	40	Diluent Control	15	65.229390	1	6.666781E-16	65.229390	1	6.666781E-16	1.000000	S*, A*

Study	Threshold Metric	Signs threshold	Weight threshold	Comparison Group A		Comparison Group B		Alternative endpoint Comparison			Original study comparison			P-value ratio	P-value ratio change
				Treatment	n	Treatment	n	Test statistic	df	p-value	Test statistic	df	p-value		
3	Average signs	≥4	24	Finafloxacin	40	Diluent Control	15	65.229390	1	6.666781E-16	65.229390	1	6.666781E-16	1.000000	S*, A*
3	Total signs	≥4	25	Finafloxacin	40	Diluent Control	15	65.229390	1	6.666781E-16	65.229390	1	6.666781E-16	1.000000	S*, A*
3	Average signs	≥4	25	Finafloxacin	40	Diluent Control	15	65.229390	1	6.666781E-16	65.229390	1	6.666781E-16	1.000000	S*, A*
3	Total signs	≥4	26	Finafloxacin	40	Diluent Control	15	65.229390	1	6.666781E-16	65.229390	1	6.666781E-16	1.000000	S*, A*
3	Average signs	≥4	26	Finafloxacin	40	Diluent Control	15	65.229390	1	6.666781E-16	65.229390	1	6.666781E-16	1.000000	S*, A*
3	Total signs	≥4	27	Finafloxacin	40	Diluent Control	15	65.229390	1	6.666781E-16	65.229390	1	6.666781E-16	1.000000	S*, A*
3	Average signs	≥4	27	Finafloxacin	40	Diluent Control	15	65.229390	1	6.666781E-16	65.229390	1	6.666781E-16	1.000000	S*, A*
3	Total signs	≥4	28	Finafloxacin	40	Diluent Control	15	65.229390	1	6.666781E-16	65.229390	1	6.666781E-16	1.000000	S*, A*
3	Average signs	≥4	28	Finafloxacin	40	Diluent Control	15	65.229390	1	6.666781E-16	65.229390	1	6.666781E-16	1.000000	S*, A*
3	Total signs	≥4	29	Finafloxacin	40	Diluent Control	15	65.229390	1	6.666781E-16	65.229390	1	6.666781E-16	1.000000	S*, A*
3	Average signs	≥4	29	Finafloxacin	40	Diluent Control	15	65.229390	1	6.666781E-16	65.229390	1	6.666781E-16	1.000000	S*, A*
3	Total signs	≥4	30	Finafloxacin	40	Diluent Control	15	65.229390	1	6.666781E-16	65.229390	1	6.666781E-16	1.000000	S*, A*
3	Average signs	≥4	30	Finafloxacin	40	Diluent Control	15	65.229390	1	6.666781E-16	65.229390	1	6.666781E-16	1.000000	S*, A*
3	Total signs	≥4	20	Doxycycline	40	Diluent Control	15	65.229390	1	6.666781E-16	65.229390	1	6.666781E-16	1.000000	S*, A*
3	Average signs	≥4	20	Doxycycline	40	Diluent Control	15	65.229390	1	6.666781E-16	65.229390	1	6.666781E-16	1.000000	S*, A*
3	Total signs	≥4	21	Doxycycline	40	Diluent Control	15	65.229390	1	6.666781E-16	65.229390	1	6.666781E-16	1.000000	S*, A*
3	Average signs	≥4	21	Doxycycline	40	Diluent Control	15	65.229390	1	6.666781E-16	65.229390	1	6.666781E-16	1.000000	S*, A*
3	Total signs	≥4	22	Doxycycline	40	Diluent Control	15	65.229390	1	6.666781E-16	65.229390	1	6.666781E-16	1.000000	S*, A*
3	Average signs	≥4	22	Doxycycline	40	Diluent Control	15	65.229390	1	6.666781E-16	65.229390	1	6.666781E-16	1.000000	S*, A*
3	Total signs	≥4	23	Doxycycline	40	Diluent Control	15	65.229390	1	6.666781E-16	65.229390	1	6.666781E-16	1.000000	S*, A*
3	Average signs	≥4	23	Doxycycline	40	Diluent Control	15	65.229390	1	6.666781E-16	65.229390	1	6.666781E-16	1.000000	S*, A*
3	Total signs	≥4	24	Doxycycline	40	Diluent Control	15	65.229390	1	6.666781E-16	65.229390	1	6.666781E-16	1.000000	S*, A*
3	Average signs	≥4	24	Doxycycline	40	Diluent Control	15	65.229390	1	6.666781E-16	65.229390	1	6.666781E-16	1.000000	S*, A*
3	Total signs	≥4	25	Doxycycline	40	Diluent Control	15	65.229390	1	6.666781E-16	65.229390	1	6.666781E-16	1.000000	S*, A*
3	Average signs	≥4	25	Doxycycline	40	Diluent Control	15	65.229390	1	6.666781E-16	65.229390	1	6.666781E-16	1.000000	S*, A*
3	Total signs	≥4	26	Doxycycline	40	Diluent Control	15	65.229390	1	6.666781E-16	65.229390	1	6.666781E-16	1.000000	S*, A*
3	Average signs	≥4	26	Doxycycline	40	Diluent Control	15	65.229390	1	6.666781E-16	65.229390	1	6.666781E-16	1.000000	S*, A*
3	Total signs	≥4	27	Doxycycline	40	Diluent Control	15	65.229390	1	6.666781E-16	65.229390	1	6.666781E-16	1.000000	S*, A*
3	Average signs	≥4	27	Doxycycline	40	Diluent Control	15	65.229390	1	6.666781E-16	65.229390	1	6.666781E-16	1.000000	S*, A*
3	Total signs	≥4	28	Doxycycline	40	Diluent Control	15	65.229390	1	6.666781E-16	65.229390	1	6.666781E-16	1.000000	S*, A*
3	Average signs	≥4	28	Doxycycline	40	Diluent Control	15	65.229390	1	6.666781E-16	65.229390	1	6.666781E-16	1.000000	S*, A*
3	Total signs	≥4	29	Doxycycline	40	Diluent Control	15	65.229390	1	6.666781E-16	65.229390	1	6.666781E-16	1.000000	S*, A*
3	Average signs	≥4	29	Doxycycline	40	Diluent Control	15	65.229390	1	6.666781E-16	65.229390	1	6.666781E-16	1.000000	S*, A*
3	Total signs	≥4	30	Doxycycline	40	Diluent Control	15	65.229390	1	6.666781E-16	65.229390	1	6.666781E-16	1.000000	S*, A*
3	Average signs	≥4	30	Doxycycline	40	Diluent Control	15	65.229390	1	6.666781E-16	65.229390	1	6.666781E-16	1.000000	S*, A*
3	Total signs	≥4	20	Doxycycline	40	Finafloxacin	40	9.483353	1	2.073447E-03	4.326465	1	3.752411E-02	0.055256	S*, A*
3	Average signs	≥4	20	Doxycycline	40	Finafloxacin	40	10.660097	1	1.094716E-03	4.326465	1	3.752411E-02	0.029174	S*, A*
3	Total signs	≥4	21	Doxycycline	40	Finafloxacin	40	7.632681	1	5.732003E-03	4.326465	1	3.752411E-02	0.152755	S*, A*
3	Average signs	≥4	21	Doxycycline	40	Finafloxacin	40	8.907966	1	2.839293E-03	4.326465	1	3.752411E-02	0.075666	S*, A*

Study	Threshold Metric	Signs threshold	Weight threshold	Comparison Group A		Comparison Group B		Alternative endpoint Comparison			Original study comparison			P-value ratio	P-value ratio change
				Treatment	n	Treatment	n	Test statistic	df	p-value	Test statistic	df	p-value		
3	Total signs	≥4	22	Doxycycline	40	Finafloxacin	40	7.631570	1	5.735536E-03	4.326465	1	3.752411E-02	0.152849	S*, A*
3	Average signs	≥4	22	Doxycycline	40	Finafloxacin	40	9.015947	1	2.676342E-03	4.326465	1	3.752411E-02	0.071323	S*, A*
3	Total signs	≥4	23	Doxycycline	40	Finafloxacin	40	5.745242	1	1.653337E-02	4.326465	1	3.752411E-02	0.440607	S*, A*
3	Average signs	≥4	23	Doxycycline	40	Finafloxacin	40	7.293627	1	6.919962E-03	4.326465	1	3.752411E-02	0.184414	S*, A*
3	Total signs	≥4	24	Doxycycline	40	Finafloxacin	40	5.508883	1	1.892012E-02	4.326465	1	3.752411E-02	0.504213	S*, A*
3	Average signs	≥4	24	Doxycycline	40	Finafloxacin	40	8.242051	1	4.093085E-03	4.326465	1	3.752411E-02	0.109079	S*, A*
3	Total signs	≥4	25	Doxycycline	40	Finafloxacin	40	4.498816	1	3.391833E-02	4.326465	1	3.752411E-02	0.903908	S*, A*
3	Average signs	≥4	25	Doxycycline	40	Finafloxacin	40	7.004438	1	8.130788E-03	4.326465	1	3.752411E-02	0.216682	S*, A*
3	Total signs	≥4	26	Doxycycline	40	Finafloxacin	40	3.531676	1	6.020692E-02	4.326465	1	3.752411E-02	1.604486	S*, A
3	Average signs	≥4	26	Doxycycline	40	Finafloxacin	40	5.960815	1	1.462727E-02	4.326465	1	3.752411E-02	0.389810	S*, A*
3	Total signs	≥4	27	Doxycycline	40	Finafloxacin	40	3.607203	1	5.752980E-02	4.326465	1	3.752411E-02	1.533142	S*, A
3	Average signs	≥4	27	Doxycycline	40	Finafloxacin	40	6.670349	1	9.803001E-03	4.326465	1	3.752411E-02	0.261245	S*, A*
3	Total signs	≥4	28	Doxycycline	40	Finafloxacin	40	4.636214	1	3.130398E-02	4.326465	1	3.752411E-02	0.834237	S*, A*
3	Average signs	≥4	28	Doxycycline	40	Finafloxacin	40	8.717642	1	3.151454E-03	4.326465	1	3.752411E-02	0.083985	S*, A*
3	Total signs	≥4	29	Doxycycline	40	Finafloxacin	40	4.668787	1	3.071561E-02	4.326465	1	3.752411E-02	0.818557	S*, A*
3	Average signs	≥4	29	Doxycycline	40	Finafloxacin	40	8.717642	1	3.151454E-03	4.326465	1	3.752411E-02	0.083985	S*, A*
3	Total signs	≥4	30	Doxycycline	40	Finafloxacin	40	4.668787	1	3.071561E-02	4.326465	1	3.752411E-02	0.818557	S*, A*
3	Average signs	≥4	30	Doxycycline	40	Finafloxacin	40	8.717642	1	3.151454E-03	4.326465	1	3.752411E-02	0.083985	S*, A*
3	Total signs	≥4	20	Finafloxacin & Doxycycline	40	Diluent Control	15	46.872846	1	7.574496E-12	46.872846	1	7.574496E-12	1.000000	S*, A*
3	Average signs	≥4	20	Finafloxacin & Doxycycline	40	Diluent Control	15	46.872846	1	7.574496E-12	46.872846	1	7.574496E-12	1.000000	S*, A*
3	Total signs	≥4	21	Finafloxacin & Doxycycline	40	Diluent Control	15	46.872846	1	7.574496E-12	46.872846	1	7.574496E-12	1.000000	S*, A*
3	Average signs	≥4	21	Finafloxacin & Doxycycline	40	Diluent Control	15	46.872846	1	7.574496E-12	46.872846	1	7.574496E-12	1.000000	S*, A*
3	Total signs	≥4	22	Finafloxacin & Doxycycline	40	Diluent Control	15	46.872846	1	7.574496E-12	46.872846	1	7.574496E-12	1.000000	S*, A*
3	Average signs	≥4	22	Finafloxacin & Doxycycline	40	Diluent Control	15	46.872846	1	7.574496E-12	46.872846	1	7.574496E-12	1.000000	S*, A*
3	Total signs	≥4	23	Finafloxacin & Doxycycline	40	Diluent Control	15	46.872846	1	7.574496E-12	46.872846	1	7.574496E-12	1.000000	S*, A*
3	Average signs	≥4	23	Finafloxacin & Doxycycline	40	Diluent Control	15	46.872846	1	7.574496E-12	46.872846	1	7.574496E-12	1.000000	S*, A*
3	Total signs	≥4	24	Finafloxacin & Doxycycline	40	Diluent Control	15	46.872846	1	7.574496E-12	46.872846	1	7.574496E-12	1.000000	S*, A*
3	Average signs	≥4	24	Finafloxacin & Doxycycline	40	Diluent Control	15	46.872846	1	7.574496E-12	46.872846	1	7.574496E-12	1.000000	S*, A*
3	Total signs	≥4	25	Finafloxacin & Doxycycline	40	Diluent Control	15	46.872846	1	7.574496E-12	46.872846	1	7.574496E-12	1.000000	S*, A*
3	Average signs	≥4	25	Finafloxacin & Doxycycline	40	Diluent Control	15	46.872846	1	7.574496E-12	46.872846	1	7.574496E-12	1.000000	S*, A*
3	Total signs	≥4	26	Finafloxacin & Doxycycline	40	Diluent Control	15	46.872846	1	7.574496E-12	46.872846	1	7.574496E-12	1.000000	S*, A*
3	Average signs	≥4	26	Finafloxacin & Doxycycline	40	Diluent Control	15	46.872846	1	7.574496E-12	46.872846	1	7.574496E-12	1.000000	S*, A*
3	Total signs	≥4	27	Finafloxacin & Doxycycline	40	Diluent Control	15	46.872846	1	7.574496E-12	46.872846	1	7.574496E-12	1.000000	S*, A*

Study	Threshold Metric	Signs threshold	Weight threshold	Comparison Group A		Comparison Group B		Alternative endpoint Comparison			Original study comparison			P-value ratio	P-value ratio change
				Treatment	n	Treatment	n	Test statistic	df	p-value	Test statistic	df	p-value		
3	Average signs	≥4	27	Finafloxacin & Doxycycline	40	Diluent Control	15	46.872846	1	7.574496E-12	46.872846	1	7.574496E-12	1.000000	S*, A*
3	Total signs	≥4	28	Finafloxacin & Doxycycline	40	Diluent Control	15	46.872846	1	7.574496E-12	46.872846	1	7.574496E-12	1.000000	S*, A*
3	Average signs	≥4	28	Finafloxacin & Doxycycline	40	Diluent Control	15	46.872846	1	7.574496E-12	46.872846	1	7.574496E-12	1.000000	S*, A*
3	Total signs	≥4	29	Finafloxacin & Doxycycline	40	Diluent Control	15	46.872846	1	7.574496E-12	46.872846	1	7.574496E-12	1.000000	S*, A*
3	Average signs	≥4	29	Finafloxacin & Doxycycline	40	Diluent Control	15	46.872846	1	7.574496E-12	46.872846	1	7.574496E-12	1.000000	S*, A*
3	Total signs	≥4	30	Finafloxacin & Doxycycline	40	Diluent Control	15	46.872846	1	7.574496E-12	46.872846	1	7.574496E-12	1.000000	S*, A*
3	Average signs	≥4	30	Finafloxacin & Doxycycline	40	Diluent Control	15	46.872846	1	7.574496E-12	46.872846	1	7.574496E-12	1.000000	S*, A*
3	Total signs	≥4	20	Finafloxacin & Doxycycline	40	Finafloxacin	40	0.003300	1	9.541903E-01	0.709539	1	3.995968E-01	2.387883	S, A
3	Average signs	≥4	20	Finafloxacin & Doxycycline	40	Finafloxacin	40	1.010082	1	3.148832E-01	0.709539	1	3.995968E-01	0.788002	S, A
3	Total signs	≥4	21	Finafloxacin & Doxycycline	40	Finafloxacin	40	0.002704	1	9.585305E-01	0.709539	1	3.995968E-01	2.398744	S, A
3	Average signs	≥4	21	Finafloxacin & Doxycycline	40	Finafloxacin	40	1.022745	1	3.118688E-01	0.709539	1	3.995968E-01	0.780459	S, A
3	Total signs	≥4	22	Finafloxacin & Doxycycline	40	Finafloxacin	40	0.003391	1	9.535607E-01	0.709539	1	3.995968E-01	2.386307	S, A
3	Average signs	≥4	22	Finafloxacin & Doxycycline	40	Finafloxacin	40	1.004097	1	3.163213E-01	0.709539	1	3.995968E-01	0.791601	S, A
3	Total signs	≥4	23	Finafloxacin & Doxycycline	40	Finafloxacin	40	0.108691	1	7.416395E-01	0.709539	1	3.995968E-01	1.855970	S, A
3	Average signs	≥4	23	Finafloxacin & Doxycycline	40	Finafloxacin	40	0.543344	1	4.610503E-01	0.709539	1	3.995968E-01	1.153789	S, A
3	Total signs	≥4	24	Finafloxacin & Doxycycline	40	Finafloxacin	40	0.108691	1	7.416395E-01	0.709539	1	3.995968E-01	1.855970	S, A
3	Average signs	≥4	24	Finafloxacin & Doxycycline	40	Finafloxacin	40	1.087494	1	2.970271E-01	0.709539	1	3.995968E-01	0.743317	S, A
3	Total signs	≥4	25	Finafloxacin & Doxycycline	40	Finafloxacin	40	0.105175	1	7.457058E-01	0.709539	1	3.995968E-01	1.866146	S, A
3	Average signs	≥4	25	Finafloxacin & Doxycycline	40	Finafloxacin	40	1.101193	1	2.940044E-01	0.709539	1	3.995968E-01	0.735753	S, A
3	Total signs	≥4	26	Finafloxacin & Doxycycline	40	Finafloxacin	40	0.462663	1	4.963822E-01	0.709539	1	3.995968E-01	1.242208	S, A
3	Average signs	≥4	26	Finafloxacin & Doxycycline	40	Finafloxacin	40	0.527210	1	4.677824E-01	0.709539	1	3.995968E-01	1.170636	S, A
3	Total signs	≥4	27	Finafloxacin & Doxycycline	40	Finafloxacin	40	0.460369	1	4.974516E-01	0.709539	1	3.995968E-01	1.244884	S, A
3	Average signs	≥4	27	Finafloxacin & Doxycycline	40	Finafloxacin	40	1.142105	1	2.852081E-01	0.709539	1	3.995968E-01	0.713740	S, A
3	Total signs	≥4	28	Finafloxacin & Doxycycline	40	Finafloxacin	40	0.629157	1	4.276646E-01	0.709539	1	3.995968E-01	1.070240	S, A
3	Average signs	≥4	28	Finafloxacin & Doxycycline	40	Finafloxacin	40	1.403252	1	2.361798E-01	0.709539	1	3.995968E-01	0.591045	S, A
3	Total signs	≥4	29	Finafloxacin & Doxycycline	40	Finafloxacin	40	0.629157	1	4.276646E-01	0.709539	1	3.995968E-01	1.070240	S, A

Study	Threshold Metric	Signs threshold	Weight threshold	Comparison Group A		Comparison Group B		Alternative endpoint Comparison			Original study comparison			P-value ratio	P-value ratio change
				Treatment	n	Treatment	n	Test statistic	df	p-value	Test statistic	df	p-value		
3	Average signs	≥4	29	Finafloxacin & Doxycycline	40	Finafloxacin	40	1.403252	1	2.361798E-01	0.709539	1	3.995968E-01	0.591045	S, A
3	Total signs	≥4	30	Finafloxacin & Doxycycline	40	Finafloxacin	40	1.299280	1	2.543448E-01	0.709539	1	3.995968E-01	0.636504	S, A
3	Average signs	≥4	30	Finafloxacin & Doxycycline	40	Finafloxacin	40	0.709539	1	3.995968E-01	0.709539	1	3.995968E-01	1.000000	S, A
3	Total signs	≥4	20	Finafloxacin & Doxycycline	40	Doxycycline	40	7.964539	1	4.770265E-03	1.597411	1	2.062705E-01	0.023126	S, A*
3	Average signs	≥4	20	Finafloxacin & Doxycycline	40	Doxycycline	40	4.967326	1	2.583055E-02	1.597411	1	2.062705E-01	0.125227	S, A*
3	Total signs	≥4	21	Finafloxacin & Doxycycline	40	Doxycycline	40	6.601857	1	1.018725E-02	1.597411	1	2.062705E-01	0.049388	S, A*
3	Average signs	≥4	21	Finafloxacin & Doxycycline	40	Doxycycline	40	3.875748	1	4.898847E-02	1.597411	1	2.062705E-01	0.237496	S, A*
3	Total signs	≥4	22	Finafloxacin & Doxycycline	40	Doxycycline	40	6.626810	1	1.004550E-02	1.597411	1	2.062705E-01	0.048701	S, A*
3	Average signs	≥4	22	Finafloxacin & Doxycycline	40	Doxycycline	40	3.846422	1	4.985222E-02	1.597411	1	2.062705E-01	0.241684	S, A*
3	Total signs	≥4	23	Finafloxacin & Doxycycline	40	Doxycycline	40	6.182532	1	1.290176E-02	1.597411	1	2.062705E-01	0.062548	S, A*
3	Average signs	≥4	23	Finafloxacin & Doxycycline	40	Doxycycline	40	3.380340	1	6.597843E-02	1.597411	1	2.062705E-01	0.319864	S, A
3	Total signs	≥4	24	Finafloxacin & Doxycycline	40	Doxycycline	40	6.070729	1	1.374402E-02	1.597411	1	2.062705E-01	0.066631	S, A*
3	Average signs	≥4	24	Finafloxacin & Doxycycline	40	Doxycycline	40	3.422159	1	6.432685E-02	1.597411	1	2.062705E-01	0.311857	S, A
3	Total signs	≥4	25	Finafloxacin & Doxycycline	40	Doxycycline	40	5.002186	1	2.531533E-02	1.597411	1	2.062705E-01	0.122729	S, A*
3	Average signs	≥4	25	Finafloxacin & Doxycycline	40	Doxycycline	40	2.573119	1	1.086932E-01	1.597411	1	2.062705E-01	0.526945	S, A
3	Total signs	≥4	26	Finafloxacin & Doxycycline	40	Doxycycline	40	5.779964	1	1.620987E-02	1.597411	1	2.062705E-01	0.078586	S, A*
3	Average signs	≥4	26	Finafloxacin & Doxycycline	40	Doxycycline	40	3.083789	1	7.907611E-02	1.597411	1	2.062705E-01	0.383361	S, A
3	Total signs	≥4	27	Finafloxacin & Doxycycline	40	Doxycycline	40	5.733638	1	1.664297E-02	1.597411	1	2.062705E-01	0.080685	S, A*
3	Average signs	≥4	27	Finafloxacin & Doxycycline	40	Doxycycline	40	2.390110	1	1.221051E-01	1.597411	1	2.062705E-01	0.591966	S, A
3	Total signs	≥4	28	Finafloxacin & Doxycycline	40	Doxycycline	40	7.072762	1	7.826453E-03	1.597411	1	2.062705E-01	0.037943	S, A*
3	Average signs	≥4	28	Finafloxacin & Doxycycline	40	Doxycycline	40	3.537868	1	5.998252E-02	1.597411	1	2.062705E-01	0.290795	S, A
3	Total signs	≥4	29	Finafloxacin & Doxycycline	40	Doxycycline	40	7.156020	1	7.471280E-03	1.597411	1	2.062705E-01	0.036221	S, A*
3	Average signs	≥4	29	Finafloxacin & Doxycycline	40	Doxycycline	40	3.537868	1	5.998252E-02	1.597411	1	2.062705E-01	0.290795	S, A
3	Total signs	≥4	30	Finafloxacin & Doxycycline	40	Doxycycline	40	9.071851	1	2.595741E-03	1.597411	1	2.062705E-01	0.012584	S, A*
3	Average signs	≥4	30	Finafloxacin & Doxycycline	40	Doxycycline	40	4.914739	1	2.662849E-02	1.597411	1	2.062705E-01	0.129095	S, A*
4	Total signs	≥4	20	Finafloxacin 36 h	45	Finafloxacin 48 h	44	0.603397	1	4.372850E-01	1.730962	1	1.882881E-01	2.322426	S, A

Study	Threshold Metric	Signs threshold	Weight threshold	Comparison Group A		Comparison Group B		Alternative endpoint Comparison			Original study comparison			P-value ratio	P-value ratio change
				Treatment	n	Treatment	n	Test statistic	df	p-value	Test statistic	df	p-value		
4	Average signs	≥4	20	Finafloxacin 36 h	45	Finafloxacin 48 h	44	0.368793	1	5.436628E-01	1.730962	1	1.882881E-01	2.887399	S, A
4	Total signs	≥4	21	Finafloxacin 36 h	45	Finafloxacin 48 h	44	0.555921	1	4.559084E-01	1.730962	1	1.882881E-01	2.421335	S, A
4	Average signs	≥4	21	Finafloxacin 36 h	45	Finafloxacin 48 h	44	0.315356	1	5.744122E-01	1.730962	1	1.882881E-01	3.050709	S, A
4	Total signs	≥4	22	Finafloxacin 36 h	45	Finafloxacin 48 h	44	0.548547	1	4.589122E-01	1.730962	1	1.882881E-01	2.437288	S, A
4	Average signs	≥4	22	Finafloxacin 36 h	45	Finafloxacin 48 h	44	0.293546	1	5.879570E-01	1.730962	1	1.882881E-01	3.122646	S, A
4	Total signs	≥4	23	Finafloxacin 36 h	45	Finafloxacin 48 h	44	0.477207	1	4.896902E-01	1.730962	1	1.882881E-01	2.600750	S, A
4	Average signs	≥4	23	Finafloxacin 36 h	45	Finafloxacin 48 h	44	0.200502	1	6.543157E-01	1.730962	1	1.882881E-01	3.475078	S, A
4	Total signs	≥4	24	Finafloxacin 36 h	45	Finafloxacin 48 h	44	0.613668	1	4.334100E-01	1.730962	1	1.882881E-01	2.301845	S, A
4	Average signs	≥4	24	Finafloxacin 36 h	45	Finafloxacin 48 h	44	0.321153	1	5.709154E-01	1.730962	1	1.882881E-01	3.032138	S, A
4	Total signs	≥4	25	Finafloxacin 36 h	45	Finafloxacin 48 h	44	0.597833	1	4.394058E-01	1.730962	1	1.882881E-01	2.333689	S, A
4	Average signs	≥4	25	Finafloxacin 36 h	45	Finafloxacin 48 h	44	0.443322	1	5.055235E-01	1.730962	1	1.882881E-01	2.684841	S, A
4	Total signs	≥4	26	Finafloxacin 36 h	45	Finafloxacin 48 h	44	0.563199	1	4.529741E-01	1.730962	1	1.882881E-01	2.405750	S, A
4	Average signs	≥4	26	Finafloxacin 36 h	45	Finafloxacin 48 h	44	0.436839	1	5.086519E-01	1.730962	1	1.882881E-01	2.701456	S, A
4	Total signs	≥4	27	Finafloxacin 36 h	45	Finafloxacin 48 h	44	0.557852	1	4.551272E-01	1.730962	1	1.882881E-01	2.417185	S, A
4	Average signs	≥4	27	Finafloxacin 36 h	45	Finafloxacin 48 h	44	0.390001	1	5.322990E-01	1.730962	1	1.882881E-01	2.827046	S, A
4	Total signs	≥4	28	Finafloxacin 36 h	45	Finafloxacin 48 h	44	0.536830	1	4.637495E-01	1.730962	1	1.882881E-01	2.462979	S, A
4	Average signs	≥4	28	Finafloxacin 36 h	45	Finafloxacin 48 h	44	0.384296	1	5.353132E-01	1.730962	1	1.882881E-01	2.843054	S, A
4	Total signs	≥4	29	Finafloxacin 36 h	45	Finafloxacin 48 h	44	0.531705	1	4.658908E-01	1.730962	1	1.882881E-01	2.474351	S, A
4	Average signs	≥4	29	Finafloxacin 36 h	45	Finafloxacin 48 h	44	0.359861	1	5.485835E-01	1.730962	1	1.882881E-01	2.913533	S, A
4	Total signs	≥4	30	Finafloxacin 36 h	45	Finafloxacin 48 h	44	0.531705	1	4.658908E-01	1.730962	1	1.882881E-01	2.474351	S, A
4	Average signs	≥4	30	Finafloxacin 36 h	45	Finafloxacin 48 h	44	0.317577	1	5.730673E-01	1.730962	1	1.882881E-01	3.043567	S, A
4	Total signs	≥4	20	Finafloxacin 36 h	45	Diluent Control	88	94.766888	1	2.141784E-22	94.766888	1	2.141784E-22	1.000000	S*, A*
4	Average signs	≥4	20	Finafloxacin 36 h	45	Diluent Control	88	94.766888	1	2.141784E-22	94.766888	1	2.141784E-22	1.000000	S*, A*
4	Total signs	≥4	21	Finafloxacin 36 h	45	Diluent Control	88	94.766888	1	2.141784E-22	94.766888	1	2.141784E-22	1.000000	S*, A*
4	Average signs	≥4	21	Finafloxacin 36 h	45	Diluent Control	88	94.766888	1	2.141784E-22	94.766888	1	2.141784E-22	1.000000	S*, A*
4	Total signs	≥4	22	Finafloxacin 36 h	45	Diluent Control	88	94.766888	1	2.141784E-22	94.766888	1	2.141784E-22	1.000000	S*, A*
4	Average signs	≥4	22	Finafloxacin 36 h	45	Diluent Control	88	94.766888	1	2.141784E-22	94.766888	1	2.141784E-22	1.000000	S*, A*
4	Total signs	≥4	23	Finafloxacin 36 h	45	Diluent Control	88	94.766888	1	2.141784E-22	94.766888	1	2.141784E-22	1.000000	S*, A*
4	Average signs	≥4	23	Finafloxacin 36 h	45	Diluent Control	88	94.766888	1	2.141784E-22	94.766888	1	2.141784E-22	1.000000	S*, A*
4	Total signs	≥4	24	Finafloxacin 36 h	45	Diluent Control	88	94.766888	1	2.141784E-22	94.766888	1	2.141784E-22	1.000000	S*, A*
4	Average signs	≥4	24	Finafloxacin 36 h	45	Diluent Control	88	94.766888	1	2.141784E-22	94.766888	1	2.141784E-22	1.000000	S*, A*
4	Total signs	≥4	25	Finafloxacin 36 h	45	Diluent Control	88	94.766888	1	2.141784E-22	94.766888	1	2.141784E-22	1.000000	S*, A*
4	Average signs	≥4	25	Finafloxacin 36 h	45	Diluent Control	88	94.766888	1	2.141784E-22	94.766888	1	2.141784E-22	1.000000	S*, A*
4	Total signs	≥4	26	Finafloxacin 36 h	45	Diluent Control	88	94.766888	1	2.141784E-22	94.766888	1	2.141784E-22	1.000000	S*, A*
4	Average signs	≥4	26	Finafloxacin 36 h	45	Diluent Control	88	94.766888	1	2.141784E-22	94.766888	1	2.141784E-22	1.000000	S*, A*
4	Total signs	≥4	27	Finafloxacin 36 h	45	Diluent Control	88	94.766888	1	2.141784E-22	94.766888	1	2.141784E-22	1.000000	S*, A*
4	Average signs	≥4	27	Finafloxacin 36 h	45	Diluent Control	88	94.766888	1	2.141784E-22	94.766888	1	2.141784E-22	1.000000	S*, A*
4	Total signs	≥4	28	Finafloxacin 36 h	45	Diluent Control	88	94.766888	1	2.141784E-22	94.766888	1	2.141784E-22	1.000000	S*, A*

Study	Threshold Metric	Signs threshold	Weight threshold	Comparison Group A		Comparison Group B		Alternative endpoint Comparison			Original study comparison			P-value ratio	P-value ratio change
				Treatment	n	Treatment	n	Test statistic	df	p-value	Test statistic	df	p-value		
4	Average signs	≥4	28	Finafloxacin 36 h	45	Diluent Control	88	94.766888	1	2.141784E-22	94.766888	1	2.141784E-22	1.000000	S*, A*
4	Total signs	≥4	29	Finafloxacin 36 h	45	Diluent Control	88	94.766888	1	2.141784E-22	94.766888	1	2.141784E-22	1.000000	S*, A*
4	Average signs	≥4	29	Finafloxacin 36 h	45	Diluent Control	88	94.766888	1	2.141784E-22	94.766888	1	2.141784E-22	1.000000	S*, A*
4	Total signs	≥4	30	Finafloxacin 36 h	45	Diluent Control	88	94.766888	1	2.141784E-22	94.766888	1	2.141784E-22	1.000000	S*, A*
4	Average signs	≥4	30	Finafloxacin 36 h	45	Diluent Control	88	94.766888	1	2.141784E-22	94.766888	1	2.141784E-22	1.000000	S*, A*
4	Total signs	≥4	20	Finafloxacin 48 h	44	Diluent Control	88	72.966845	1	1.318462E-17	72.966845	1	1.318462E-17	1.000000	S*, A*
4	Average signs	≥4	20	Finafloxacin 48 h	44	Diluent Control	88	72.966845	1	1.318462E-17	72.966845	1	1.318462E-17	1.000000	S*, A*
4	Total signs	≥4	21	Finafloxacin 48 h	44	Diluent Control	88	72.966845	1	1.318462E-17	72.966845	1	1.318462E-17	1.000000	S*, A*
4	Average signs	≥4	21	Finafloxacin 48 h	44	Diluent Control	88	72.966845	1	1.318462E-17	72.966845	1	1.318462E-17	1.000000	S*, A*
4	Total signs	≥4	22	Finafloxacin 48 h	44	Diluent Control	88	72.966845	1	1.318462E-17	72.966845	1	1.318462E-17	1.000000	S*, A*
4	Average signs	≥4	22	Finafloxacin 48 h	44	Diluent Control	88	72.966845	1	1.318462E-17	72.966845	1	1.318462E-17	1.000000	S*, A*
4	Total signs	≥4	23	Finafloxacin 48 h	44	Diluent Control	88	72.966845	1	1.318462E-17	72.966845	1	1.318462E-17	1.000000	S*, A*
4	Average signs	≥4	23	Finafloxacin 48 h	44	Diluent Control	88	72.966845	1	1.318462E-17	72.966845	1	1.318462E-17	1.000000	S*, A*
4	Total signs	≥4	24	Finafloxacin 48 h	44	Diluent Control	88	72.966845	1	1.318462E-17	72.966845	1	1.318462E-17	1.000000	S*, A*
4	Average signs	≥4	24	Finafloxacin 48 h	44	Diluent Control	88	72.966845	1	1.318462E-17	72.966845	1	1.318462E-17	1.000000	S*, A*
4	Total signs	≥4	25	Finafloxacin 48 h	44	Diluent Control	88	72.966845	1	1.318462E-17	72.966845	1	1.318462E-17	1.000000	S*, A*
4	Average signs	≥4	25	Finafloxacin 48 h	44	Diluent Control	88	72.966845	1	1.318462E-17	72.966845	1	1.318462E-17	1.000000	S*, A*
4	Total signs	≥4	26	Finafloxacin 48 h	44	Diluent Control	88	72.966845	1	1.318462E-17	72.966845	1	1.318462E-17	1.000000	S*, A*
4	Average signs	≥4	26	Finafloxacin 48 h	44	Diluent Control	88	72.966845	1	1.318462E-17	72.966845	1	1.318462E-17	1.000000	S*, A*
4	Total signs	≥4	27	Finafloxacin 48 h	44	Diluent Control	88	72.966845	1	1.318462E-17	72.966845	1	1.318462E-17	1.000000	S*, A*
4	Average signs	≥4	27	Finafloxacin 48 h	44	Diluent Control	88	72.966845	1	1.318462E-17	72.966845	1	1.318462E-17	1.000000	S*, A*
4	Total signs	≥4	28	Finafloxacin 48 h	44	Diluent Control	88	72.966845	1	1.318462E-17	72.966845	1	1.318462E-17	1.000000	S*, A*
4	Average signs	≥4	28	Finafloxacin 48 h	44	Diluent Control	88	72.966845	1	1.318462E-17	72.966845	1	1.318462E-17	1.000000	S*, A*
4	Total signs	≥4	29	Finafloxacin 48 h	44	Diluent Control	88	72.966845	1	1.318462E-17	72.966845	1	1.318462E-17	1.000000	S*, A*
4	Average signs	≥4	29	Finafloxacin 48 h	44	Diluent Control	88	72.966845	1	1.318462E-17	72.966845	1	1.318462E-17	1.000000	S*, A*
4	Total signs	≥4	30	Finafloxacin 48 h	44	Diluent Control	88	72.966845	1	1.318462E-17	72.966845	1	1.318462E-17	1.000000	S*, A*
4	Average signs	≥4	30	Finafloxacin 48 h	44	Diluent Control	88	72.966845	1	1.318462E-17	72.966845	1	1.318462E-17	1.000000	S*, A*
1	Total signs	≥5	20	Finafloxacin	45	Diluent Control	15	83.192904	1	7.442441E-20	83.192904	1	7.442441E-20	1.000000	S*, A*
1	Average signs	≥5	20	Finafloxacin	45	Diluent Control	15	83.192904	1	7.442441E-20	83.192904	1	7.442441E-20	1.000000	S*, A*
1	Total signs	≥5	21	Finafloxacin	45	Diluent Control	15	83.192904	1	7.442441E-20	83.192904	1	7.442441E-20	1.000000	S*, A*
1	Average signs	≥5	21	Finafloxacin	45	Diluent Control	15	83.192904	1	7.442441E-20	83.192904	1	7.442441E-20	1.000000	S*, A*
1	Total signs	≥5	22	Finafloxacin	45	Diluent Control	15	83.192904	1	7.442441E-20	83.192904	1	7.442441E-20	1.000000	S*, A*
1	Average signs	≥5	22	Finafloxacin	45	Diluent Control	15	83.192904	1	7.442441E-20	83.192904	1	7.442441E-20	1.000000	S*, A*
1	Total signs	≥5	23	Finafloxacin	45	Diluent Control	15	83.192904	1	7.442441E-20	83.192904	1	7.442441E-20	1.000000	S*, A*
1	Average signs	≥5	23	Finafloxacin	45	Diluent Control	15	83.192904	1	7.442441E-20	83.192904	1	7.442441E-20	1.000000	S*, A*
1	Total signs	≥5	24	Finafloxacin	45	Diluent Control	15	83.192904	1	7.442441E-20	83.192904	1	7.442441E-20	1.000000	S*, A*
1	Average signs	≥5	24	Finafloxacin	45	Diluent Control	15	83.192904	1	7.442441E-20	83.192904	1	7.442441E-20	1.000000	S*, A*
1	Total signs	≥5	25	Finafloxacin	45	Diluent Control	15	83.192904	1	7.442441E-20	83.192904	1	7.442441E-20	1.000000	S*, A*

Study	Threshold Metric	Signs threshold	Weight threshold	Comparison Group A		Comparison Group B		Alternative endpoint Comparison			Original study comparison			P-value ratio	P-value ratio change
				Treatment	n	Treatment	n	Test statistic	df	p-value	Test statistic	df	p-value		
1	Average signs	≥5	25	Finafloxacin	45	Diluent Control	15	83.192904	1	7.442441E-20	83.192904	1	7.442441E-20	1.000000	S*, A*
1	Total signs	≥5	26	Finafloxacin	45	Diluent Control	15	83.192904	1	7.442441E-20	83.192904	1	7.442441E-20	1.000000	S*, A*
1	Average signs	≥5	26	Finafloxacin	45	Diluent Control	15	83.192904	1	7.442441E-20	83.192904	1	7.442441E-20	1.000000	S*, A*
1	Total signs	≥5	27	Finafloxacin	45	Diluent Control	15	83.192904	1	7.442441E-20	83.192904	1	7.442441E-20	1.000000	S*, A*
1	Average signs	≥5	27	Finafloxacin	45	Diluent Control	15	83.192904	1	7.442441E-20	83.192904	1	7.442441E-20	1.000000	S*, A*
1	Total signs	≥5	28	Finafloxacin	45	Diluent Control	15	83.192904	1	7.442441E-20	83.192904	1	7.442441E-20	1.000000	S*, A*
1	Average signs	≥5	28	Finafloxacin	45	Diluent Control	15	83.192904	1	7.442441E-20	83.192904	1	7.442441E-20	1.000000	S*, A*
1	Total signs	≥5	29	Finafloxacin	45	Diluent Control	15	83.192904	1	7.442441E-20	83.192904	1	7.442441E-20	1.000000	S*, A*
1	Average signs	≥5	29	Finafloxacin	45	Diluent Control	15	83.192904	1	7.442441E-20	83.192904	1	7.442441E-20	1.000000	S*, A*
1	Total signs	≥5	30	Finafloxacin	45	Diluent Control	15	83.192904	1	7.442441E-20	83.192904	1	7.442441E-20	1.000000	S*, A*
1	Average signs	≥5	30	Finafloxacin	45	Diluent Control	15	83.192904	1	7.442441E-20	83.192904	1	7.442441E-20	1.000000	S*, A*
1	Total signs	≥5	20	Co-trimoxazole	45	Diluent Control	15	83.192904	1	7.442441E-20	83.192904	1	7.442441E-20	1.000000	S*, A*
1	Average signs	≥5	20	Co-trimoxazole	45	Diluent Control	15	83.192904	1	7.442441E-20	83.192904	1	7.442441E-20	1.000000	S*, A*
1	Total signs	≥5	21	Co-trimoxazole	45	Diluent Control	15	83.192904	1	7.442441E-20	83.192904	1	7.442441E-20	1.000000	S*, A*
1	Average signs	≥5	21	Co-trimoxazole	45	Diluent Control	15	83.192904	1	7.442441E-20	83.192904	1	7.442441E-20	1.000000	S*, A*
1	Total signs	≥5	22	Co-trimoxazole	45	Diluent Control	15	83.192904	1	7.442441E-20	83.192904	1	7.442441E-20	1.000000	S*, A*
1	Average signs	≥5	22	Co-trimoxazole	45	Diluent Control	15	83.192904	1	7.442441E-20	83.192904	1	7.442441E-20	1.000000	S*, A*
1	Total signs	≥5	23	Co-trimoxazole	45	Diluent Control	15	83.192904	1	7.442441E-20	83.192904	1	7.442441E-20	1.000000	S*, A*
1	Average signs	≥5	23	Co-trimoxazole	45	Diluent Control	15	83.192904	1	7.442441E-20	83.192904	1	7.442441E-20	1.000000	S*, A*
1	Total signs	≥5	24	Co-trimoxazole	45	Diluent Control	15	83.192904	1	7.442441E-20	83.192904	1	7.442441E-20	1.000000	S*, A*
1	Average signs	≥5	24	Co-trimoxazole	45	Diluent Control	15	83.192904	1	7.442441E-20	83.192904	1	7.442441E-20	1.000000	S*, A*
1	Total signs	≥5	25	Co-trimoxazole	45	Diluent Control	15	83.192904	1	7.442441E-20	83.192904	1	7.442441E-20	1.000000	S*, A*
1	Average signs	≥5	25	Co-trimoxazole	45	Diluent Control	15	83.192904	1	7.442441E-20	83.192904	1	7.442441E-20	1.000000	S*, A*
1	Total signs	≥5	26	Co-trimoxazole	45	Diluent Control	15	83.192904	1	7.442441E-20	83.192904	1	7.442441E-20	1.000000	S*, A*
1	Average signs	≥5	26	Co-trimoxazole	45	Diluent Control	15	83.192904	1	7.442441E-20	83.192904	1	7.442441E-20	1.000000	S*, A*
1	Total signs	≥5	27	Co-trimoxazole	45	Diluent Control	15	83.192904	1	7.442441E-20	83.192904	1	7.442441E-20	1.000000	S*, A*
1	Average signs	≥5	27	Co-trimoxazole	45	Diluent Control	15	83.192904	1	7.442441E-20	83.192904	1	7.442441E-20	1.000000	S*, A*
1	Total signs	≥5	28	Co-trimoxazole	45	Diluent Control	15	83.192904	1	7.442441E-20	83.192904	1	7.442441E-20	1.000000	S*, A*
1	Average signs	≥5	28	Co-trimoxazole	45	Diluent Control	15	83.192904	1	7.442441E-20	83.192904	1	7.442441E-20	1.000000	S*, A*
1	Total signs	≥5	29	Co-trimoxazole	45	Diluent Control	15	83.192904	1	7.442441E-20	83.192904	1	7.442441E-20	1.000000	S*, A*
1	Average signs	≥5	29	Co-trimoxazole	45	Diluent Control	15	83.192904	1	7.442441E-20	83.192904	1	7.442441E-20	1.000000	S*, A*
1	Total signs	≥5	30	Co-trimoxazole	45	Diluent Control	15	83.192904	1	7.442441E-20	83.192904	1	7.442441E-20	1.000000	S*, A*
1	Average signs	≥5	30	Co-trimoxazole	45	Diluent Control	15	83.192904	1	7.442441E-20	83.192904	1	7.442441E-20	1.000000	S*, A*
1	Total signs	≥5	20	Co-trimoxazole	45	Finafloxacin	45	3.008698	1	8.281878E-02	11.725428	1	6.165186E-04	134.332979	S*, A
1	Average signs	≥5	20	Co-trimoxazole	45	Finafloxacin	45	2.210295	1	1.370924E-01	11.725428	1	6.165186E-04	222.365395	S*, A
1	Total signs	≥5	21	Co-trimoxazole	45	Finafloxacin	45	5.530084	1	1.869221E-02	11.725428	1	6.165186E-04	30.318971	S*, A*
1	Average signs	≥5	21	Co-trimoxazole	45	Finafloxacin	45	4.724232	1	2.974046E-02	11.725428	1	6.165186E-04	48.239358	S*, A*
1	Total signs	≥5	22	Co-trimoxazole	45	Finafloxacin	45	8.359797	1	3.836133E-03	11.725428	1	6.165186E-04	6.222250	S*, A*

Study	Threshold Metric	Signs threshold	Weight threshold	Comparison Group A		Comparison Group B		Alternative endpoint Comparison			Original study comparison			P-value ratio	P-value ratio change
				Treatment	n	Treatment	n	Test statistic	df	p-value	Test statistic	df	p-value		
1	Average signs	≥5	22	Co-trimoxazole	45	Finafloxacin	45	7.025823	1	8.034256E-03	11.725428	1	6.165186E-04	13.031651	S*, A*
1	Total signs	≥5	23	Co-trimoxazole	45	Finafloxacin	45	12.815275	1	3.438008E-04	11.725428	1	6.165186E-04	0.557649	S*, A*
1	Average signs	≥5	23	Co-trimoxazole	45	Finafloxacin	45	11.515654	1	6.901255E-04	11.725428	1	6.165186E-04	1.119391	S*, A*
1	Total signs	≥5	24	Co-trimoxazole	45	Finafloxacin	45	12.894554	1	3.295396E-04	11.725428	1	6.165186E-04	0.534517	S*, A*
1	Average signs	≥5	24	Co-trimoxazole	45	Finafloxacin	45	12.017587	1	5.270089E-04	11.725428	1	6.165186E-04	0.854814	S*, A*
1	Total signs	≥5	25	Co-trimoxazole	45	Finafloxacin	45	12.839425	1	3.393919E-04	11.725428	1	6.165186E-04	0.550497	S*, A*
1	Average signs	≥5	25	Co-trimoxazole	45	Finafloxacin	45	12.130275	1	4.960982E-04	11.725428	1	6.165186E-04	0.804677	S*, A*
1	Total signs	≥5	26	Co-trimoxazole	45	Finafloxacin	45	12.912319	1	3.264266E-04	11.725428	1	6.165186E-04	0.529468	S*, A*
1	Average signs	≥5	26	Co-trimoxazole	45	Finafloxacin	45	12.278893	1	4.581099E-04	11.725428	1	6.165186E-04	0.743059	S*, A*
1	Total signs	≥5	27	Co-trimoxazole	45	Finafloxacin	45	16.098652	1	6.012680E-05	11.725428	1	6.165186E-04	0.097526	S*, A*
1	Average signs	≥5	27	Co-trimoxazole	45	Finafloxacin	45	15.184937	1	9.747787E-05	11.725428	1	6.165186E-04	0.158110	S*, A*
1	Total signs	≥5	28	Co-trimoxazole	45	Finafloxacin	45	18.064275	1	2.135713E-05	11.725428	1	6.165186E-04	0.034642	S*, A*
1	Average signs	≥5	28	Co-trimoxazole	45	Finafloxacin	45	17.199327	1	3.365555E-05	11.725428	1	6.165186E-04	0.054590	S*, A*
1	Total signs	≥5	29	Co-trimoxazole	45	Finafloxacin	45	18.326159	1	1.861337E-05	11.725428	1	6.165186E-04	0.030191	S*, A*
1	Average signs	≥5	29	Co-trimoxazole	45	Finafloxacin	45	17.199327	1	3.365555E-05	11.725428	1	6.165186E-04	0.054590	S*, A*
1	Total signs	≥5	30	Co-trimoxazole	45	Finafloxacin	45	18.326159	1	1.861337E-05	11.725428	1	6.165186E-04	0.030191	S*, A*
1	Average signs	≥5	30	Co-trimoxazole	45	Finafloxacin	45	17.393597	1	3.038478E-05	11.725428	1	6.165186E-04	0.049284	S*, A*
2	Total signs	≥5	20	Finafloxacin	90	Diluent Control	20	136.184833	1	1.817952E-31	136.184833	1	1.817952E-31	1.000000	S*, A*
2	Average signs	≥5	20	Finafloxacin	90	Diluent Control	20	136.184833	1	1.817952E-31	136.184833	1	1.817952E-31	1.000000	S*, A*
2	Total signs	≥5	21	Finafloxacin	90	Diluent Control	20	136.184833	1	1.817952E-31	136.184833	1	1.817952E-31	1.000000	S*, A*
2	Average signs	≥5	21	Finafloxacin	90	Diluent Control	20	136.184833	1	1.817952E-31	136.184833	1	1.817952E-31	1.000000	S*, A*
2	Total signs	≥5	22	Finafloxacin	90	Diluent Control	20	136.184833	1	1.817952E-31	136.184833	1	1.817952E-31	1.000000	S*, A*
2	Average signs	≥5	22	Finafloxacin	90	Diluent Control	20	136.184833	1	1.817952E-31	136.184833	1	1.817952E-31	1.000000	S*, A*
2	Total signs	≥5	23	Finafloxacin	90	Diluent Control	20	136.184833	1	1.817952E-31	136.184833	1	1.817952E-31	1.000000	S*, A*
2	Average signs	≥5	23	Finafloxacin	90	Diluent Control	20	136.184833	1	1.817952E-31	136.184833	1	1.817952E-31	1.000000	S*, A*
2	Total signs	≥5	24	Finafloxacin	90	Diluent Control	20	136.184833	1	1.817952E-31	136.184833	1	1.817952E-31	1.000000	S*, A*
2	Average signs	≥5	24	Finafloxacin	90	Diluent Control	20	136.184833	1	1.817952E-31	136.184833	1	1.817952E-31	1.000000	S*, A*
2	Total signs	≥5	25	Finafloxacin	90	Diluent Control	20	136.184833	1	1.817952E-31	136.184833	1	1.817952E-31	1.000000	S*, A*
2	Average signs	≥5	25	Finafloxacin	90	Diluent Control	20	136.184833	1	1.817952E-31	136.184833	1	1.817952E-31	1.000000	S*, A*
2	Total signs	≥5	26	Finafloxacin	90	Diluent Control	20	136.184833	1	1.817952E-31	136.184833	1	1.817952E-31	1.000000	S*, A*
2	Average signs	≥5	26	Finafloxacin	90	Diluent Control	20	136.184833	1	1.817952E-31	136.184833	1	1.817952E-31	1.000000	S*, A*
2	Total signs	≥5	27	Finafloxacin	90	Diluent Control	20	136.184833	1	1.817952E-31	136.184833	1	1.817952E-31	1.000000	S*, A*
2	Average signs	≥5	27	Finafloxacin	90	Diluent Control	20	136.184833	1	1.817952E-31	136.184833	1	1.817952E-31	1.000000	S*, A*
2	Total signs	≥5	28	Finafloxacin	90	Diluent Control	20	136.184833	1	1.817952E-31	136.184833	1	1.817952E-31	1.000000	S*, A*
2	Average signs	≥5	28	Finafloxacin	90	Diluent Control	20	136.184833	1	1.817952E-31	136.184833	1	1.817952E-31	1.000000	S*, A*
2	Total signs	≥5	29	Finafloxacin	90	Diluent Control	20	136.184833	1	1.817952E-31	136.184833	1	1.817952E-31	1.000000	S*, A*
2	Average signs	≥5	29	Finafloxacin	90	Diluent Control	20	136.184833	1	1.817952E-31	136.184833	1	1.817952E-31	1.000000	S*, A*
2	Total signs	≥5	30	Finafloxacin	90	Diluent Control	20	136.184833	1	1.817952E-31	136.184833	1	1.817952E-31	1.000000	S*, A*

Study	Threshold Metric	Signs threshold	Weight threshold	Comparison Group A		Comparison Group B		Alternative endpoint Comparison			Original study comparison			P-value ratio	P-value ratio change
				Treatment	n	Treatment	n	Test statistic	df	p-value	Test statistic	df	p-value		
2	Average signs	≥5	30	Finafloxacin	90	Diluent Control	20	136.184833	1	1.817952E-31	136.184833	1	1.817952E-31	1.000000	S*, A*
3	Total signs	≥5	20	Finafloxacin	40	Diluent Control	15	65.229390	1	6.666781E-16	65.229390	1	6.666781E-16	1.000000	S*, A*
3	Average signs	≥5	20	Finafloxacin	40	Diluent Control	15	65.229390	1	6.666781E-16	65.229390	1	6.666781E-16	1.000000	S*, A*
3	Total signs	≥5	21	Finafloxacin	40	Diluent Control	15	65.229390	1	6.666781E-16	65.229390	1	6.666781E-16	1.000000	S*, A*
3	Average signs	≥5	21	Finafloxacin	40	Diluent Control	15	65.229390	1	6.666781E-16	65.229390	1	6.666781E-16	1.000000	S*, A*
3	Total signs	≥5	22	Finafloxacin	40	Diluent Control	15	65.229390	1	6.666781E-16	65.229390	1	6.666781E-16	1.000000	S*, A*
3	Average signs	≥5	22	Finafloxacin	40	Diluent Control	15	65.229390	1	6.666781E-16	65.229390	1	6.666781E-16	1.000000	S*, A*
3	Total signs	≥5	23	Finafloxacin	40	Diluent Control	15	65.229390	1	6.666781E-16	65.229390	1	6.666781E-16	1.000000	S*, A*
3	Average signs	≥5	23	Finafloxacin	40	Diluent Control	15	65.229390	1	6.666781E-16	65.229390	1	6.666781E-16	1.000000	S*, A*
3	Total signs	≥5	24	Finafloxacin	40	Diluent Control	15	65.229390	1	6.666781E-16	65.229390	1	6.666781E-16	1.000000	S*, A*
3	Average signs	≥5	24	Finafloxacin	40	Diluent Control	15	65.229390	1	6.666781E-16	65.229390	1	6.666781E-16	1.000000	S*, A*
3	Total signs	≥5	25	Finafloxacin	40	Diluent Control	15	65.229390	1	6.666781E-16	65.229390	1	6.666781E-16	1.000000	S*, A*
3	Average signs	≥5	25	Finafloxacin	40	Diluent Control	15	65.229390	1	6.666781E-16	65.229390	1	6.666781E-16	1.000000	S*, A*
3	Total signs	≥5	26	Finafloxacin	40	Diluent Control	15	65.229390	1	6.666781E-16	65.229390	1	6.666781E-16	1.000000	S*, A*
3	Average signs	≥5	26	Finafloxacin	40	Diluent Control	15	65.229390	1	6.666781E-16	65.229390	1	6.666781E-16	1.000000	S*, A*
3	Total signs	≥5	27	Finafloxacin	40	Diluent Control	15	65.229390	1	6.666781E-16	65.229390	1	6.666781E-16	1.000000	S*, A*
3	Average signs	≥5	27	Finafloxacin	40	Diluent Control	15	65.229390	1	6.666781E-16	65.229390	1	6.666781E-16	1.000000	S*, A*
3	Total signs	≥5	28	Finafloxacin	40	Diluent Control	15	65.229390	1	6.666781E-16	65.229390	1	6.666781E-16	1.000000	S*, A*
3	Average signs	≥5	28	Finafloxacin	40	Diluent Control	15	65.229390	1	6.666781E-16	65.229390	1	6.666781E-16	1.000000	S*, A*
3	Total signs	≥5	29	Finafloxacin	40	Diluent Control	15	65.229390	1	6.666781E-16	65.229390	1	6.666781E-16	1.000000	S*, A*
3	Average signs	≥5	29	Finafloxacin	40	Diluent Control	15	65.229390	1	6.666781E-16	65.229390	1	6.666781E-16	1.000000	S*, A*
3	Total signs	≥5	30	Finafloxacin	40	Diluent Control	15	65.229390	1	6.666781E-16	65.229390	1	6.666781E-16	1.000000	S*, A*
3	Average signs	≥5	30	Finafloxacin	40	Diluent Control	15	65.229390	1	6.666781E-16	65.229390	1	6.666781E-16	1.000000	S*, A*
3	Total signs	≥5	20	Doxycycline	40	Diluent Control	15	65.229390	1	6.666781E-16	65.229390	1	6.666781E-16	1.000000	S*, A*
3	Average signs	≥5	20	Doxycycline	40	Diluent Control	15	65.229390	1	6.666781E-16	65.229390	1	6.666781E-16	1.000000	S*, A*
3	Total signs	≥5	21	Doxycycline	40	Diluent Control	15	65.229390	1	6.666781E-16	65.229390	1	6.666781E-16	1.000000	S*, A*
3	Average signs	≥5	21	Doxycycline	40	Diluent Control	15	65.229390	1	6.666781E-16	65.229390	1	6.666781E-16	1.000000	S*, A*
3	Total signs	≥5	22	Doxycycline	40	Diluent Control	15	65.229390	1	6.666781E-16	65.229390	1	6.666781E-16	1.000000	S*, A*
3	Average signs	≥5	22	Doxycycline	40	Diluent Control	15	65.229390	1	6.666781E-16	65.229390	1	6.666781E-16	1.000000	S*, A*
3	Total signs	≥5	23	Doxycycline	40	Diluent Control	15	65.229390	1	6.666781E-16	65.229390	1	6.666781E-16	1.000000	S*, A*
3	Average signs	≥5	23	Doxycycline	40	Diluent Control	15	65.229390	1	6.666781E-16	65.229390	1	6.666781E-16	1.000000	S*, A*
3	Total signs	≥5	24	Doxycycline	40	Diluent Control	15	65.229390	1	6.666781E-16	65.229390	1	6.666781E-16	1.000000	S*, A*
3	Average signs	≥5	24	Doxycycline	40	Diluent Control	15	65.229390	1	6.666781E-16	65.229390	1	6.666781E-16	1.000000	S*, A*
3	Total signs	≥5	25	Doxycycline	40	Diluent Control	15	65.229390	1	6.666781E-16	65.229390	1	6.666781E-16	1.000000	S*, A*
3	Average signs	≥5	25	Doxycycline	40	Diluent Control	15	65.229390	1	6.666781E-16	65.229390	1	6.666781E-16	1.000000	S*, A*
3	Total signs	≥5	26	Doxycycline	40	Diluent Control	15	65.229390	1	6.666781E-16	65.229390	1	6.666781E-16	1.000000	S*, A*
3	Average signs	≥5	26	Doxycycline	40	Diluent Control	15	65.229390	1	6.666781E-16	65.229390	1	6.666781E-16	1.000000	S*, A*
3	Total signs	≥5	27	Doxycycline	40	Diluent Control	15	65.229390	1	6.666781E-16	65.229390	1	6.666781E-16	1.000000	S*, A*

Study	Threshold Metric	Signs threshold	Weight threshold	Comparison Group A		Comparison Group B		Alternative endpoint Comparison			Original study comparison			P-value ratio	P-value ratio change
				Treatment	n	Treatment	n	Test statistic	df	p-value	Test statistic	df	p-value		
3	Average signs	≥5	27	Doxycycline	40	Diluent Control	15	65.229390	1	6.666781E-16	65.229390	1	6.666781E-16	1.000000	S*, A*
3	Total signs	≥5	28	Doxycycline	40	Diluent Control	15	65.229390	1	6.666781E-16	65.229390	1	6.666781E-16	1.000000	S*, A*
3	Average signs	≥5	28	Doxycycline	40	Diluent Control	15	65.229390	1	6.666781E-16	65.229390	1	6.666781E-16	1.000000	S*, A*
3	Total signs	≥5	29	Doxycycline	40	Diluent Control	15	65.229390	1	6.666781E-16	65.229390	1	6.666781E-16	1.000000	S*, A*
3	Average signs	≥5	29	Doxycycline	40	Diluent Control	15	65.229390	1	6.666781E-16	65.229390	1	6.666781E-16	1.000000	S*, A*
3	Total signs	≥5	30	Doxycycline	40	Diluent Control	15	65.229390	1	6.666781E-16	65.229390	1	6.666781E-16	1.000000	S*, A*
3	Average signs	≥5	30	Doxycycline	40	Diluent Control	15	65.229390	1	6.666781E-16	65.229390	1	6.666781E-16	1.000000	S*, A*
3	Total signs	≥5	20	Doxycycline	40	Finafloxacin	40	9.494787	1	2.060565E-03	4.326465	1	3.752411E-02	0.054913	S*, A*
3	Average signs	≥5	20	Doxycycline	40	Finafloxacin	40	7.520325	1	6.100668E-03	4.326465	1	3.752411E-02	0.162580	S*, A*
3	Total signs	≥5	21	Doxycycline	40	Finafloxacin	40	7.922800	1	4.881567E-03	4.326465	1	3.752411E-02	0.130092	S*, A*
3	Average signs	≥5	21	Doxycycline	40	Finafloxacin	40	6.133141	1	1.326710E-02	4.326465	1	3.752411E-02	0.353562	S*, A*
3	Total signs	≥5	22	Doxycycline	40	Finafloxacin	40	7.921558	1	4.884919E-03	4.326465	1	3.752411E-02	0.130181	S*, A*
3	Average signs	≥5	22	Doxycycline	40	Finafloxacin	40	6.131944	1	1.327608E-02	4.326465	1	3.752411E-02	0.353801	S*, A*
3	Total signs	≥5	23	Doxycycline	40	Finafloxacin	40	6.120132	1	1.336508E-02	4.326465	1	3.752411E-02	0.356173	S*, A*
3	Average signs	≥5	23	Doxycycline	40	Finafloxacin	40	4.410026	1	3.572831E-02	4.326465	1	3.752411E-02	0.952143	S*, A*
3	Total signs	≥5	24	Doxycycline	40	Finafloxacin	40	7.083096	1	7.781446E-03	4.326465	1	3.752411E-02	0.207372	S*, A*
3	Average signs	≥5	24	Doxycycline	40	Finafloxacin	40	5.337381	1	2.087280E-02	4.326465	1	3.752411E-02	0.556251	S*, A*
3	Total signs	≥5	25	Doxycycline	40	Finafloxacin	40	5.930439	1	1.488153E-02	4.326465	1	3.752411E-02	0.396586	S*, A*
3	Average signs	≥5	25	Doxycycline	40	Finafloxacin	40	4.394122	1	3.606301E-02	4.326465	1	3.752411E-02	0.961063	S*, A*
3	Total signs	≥5	26	Doxycycline	40	Finafloxacin	40	4.962875	1	2.589712E-02	4.326465	1	3.752411E-02	0.690146	S*, A*
3	Average signs	≥5	26	Doxycycline	40	Finafloxacin	40	3.457570	1	6.296279E-02	4.326465	1	3.752411E-02	1.677929	S*, A
3	Total signs	≥5	27	Doxycycline	40	Finafloxacin	40	5.480121	1	1.923392E-02	4.326465	1	3.752411E-02	0.512575	S*, A*
3	Average signs	≥5	27	Doxycycline	40	Finafloxacin	40	3.798393	1	5.130181E-02	4.326465	1	3.752411E-02	1.367169	S*, A
3	Total signs	≥5	28	Doxycycline	40	Finafloxacin	40	7.230527	1	7.167418E-03	4.326465	1	3.752411E-02	0.191008	S*, A*
3	Average signs	≥5	28	Doxycycline	40	Finafloxacin	40	5.521333	1	1.878594E-02	4.326465	1	3.752411E-02	0.500637	S*, A*
3	Total signs	≥5	29	Doxycycline	40	Finafloxacin	40	7.230527	1	7.167418E-03	4.326465	1	3.752411E-02	0.191008	S*, A*
3	Average signs	≥5	29	Doxycycline	40	Finafloxacin	40	5.521333	1	1.878594E-02	4.326465	1	3.752411E-02	0.500637	S*, A*
3	Total signs	≥5	30	Doxycycline	40	Finafloxacin	40	7.230527	1	7.167418E-03	4.326465	1	3.752411E-02	0.191008	S*, A*
3	Average signs	≥5	30	Doxycycline	40	Finafloxacin	40	5.521333	1	1.878594E-02	4.326465	1	3.752411E-02	0.500637	S*, A*
3	Total signs	≥5	20	Finafloxacin & Doxycycline	40	Diluent Control	15	46.872846	1	7.574496E-12	46.872846	1	7.574496E-12	1.000000	S*, A*
3	Average signs	≥5	20	Finafloxacin & Doxycycline	40	Diluent Control	15	46.872846	1	7.574496E-12	46.872846	1	7.574496E-12	1.000000	S*, A*
3	Total signs	≥5	21	Finafloxacin & Doxycycline	40	Diluent Control	15	46.872846	1	7.574496E-12	46.872846	1	7.574496E-12	1.000000	S*, A*
3	Average signs	≥5	21	Finafloxacin & Doxycycline	40	Diluent Control	15	46.872846	1	7.574496E-12	46.872846	1	7.574496E-12	1.000000	S*, A*
3	Total signs	≥5	22	Finafloxacin & Doxycycline	40	Diluent Control	15	46.872846	1	7.574496E-12	46.872846	1	7.574496E-12	1.000000	S*, A*
3	Average signs	≥5	22	Finafloxacin & Doxycycline	40	Diluent Control	15	46.872846	1	7.574496E-12	46.872846	1	7.574496E-12	1.000000	S*, A*
3	Total signs	≥5	23	Finafloxacin & Doxycycline	40	Diluent Control	15	46.872846	1	7.574496E-12	46.872846	1	7.574496E-12	1.000000	S*, A*
3	Average signs	≥5	23	Finafloxacin & Doxycycline	40	Diluent Control	15	46.872846	1	7.574496E-12	46.872846	1	7.574496E-12	1.000000	S*, A*

Study	Threshold Metric	Signs threshold	Weight threshold	Comparison Group A		Comparison Group B		Alternative endpoint Comparison			Original study comparison			P-value ratio	P-value ratio change
				Treatment	n	Treatment	n	Test statistic	df	p-value	Test statistic	df	p-value		
3	Total signs	≥5	24	Finafloxacin & Doxycycline	40	Diluent Control	15	46.872846	1	7.574496E-12	46.872846	1	7.574496E-12	1.000000	S*, A*
3	Average signs	≥5	24	Finafloxacin & Doxycycline	40	Diluent Control	15	46.872846	1	7.574496E-12	46.872846	1	7.574496E-12	1.000000	S*, A*
3	Total signs	≥5	25	Finafloxacin & Doxycycline	40	Diluent Control	15	46.872846	1	7.574496E-12	46.872846	1	7.574496E-12	1.000000	S*, A*
3	Average signs	≥5	25	Finafloxacin & Doxycycline	40	Diluent Control	15	46.872846	1	7.574496E-12	46.872846	1	7.574496E-12	1.000000	S*, A*
3	Total signs	≥5	26	Finafloxacin & Doxycycline	40	Diluent Control	15	46.872846	1	7.574496E-12	46.872846	1	7.574496E-12	1.000000	S*, A*
3	Average signs	≥5	26	Finafloxacin & Doxycycline	40	Diluent Control	15	46.872846	1	7.574496E-12	46.872846	1	7.574496E-12	1.000000	S*, A*
3	Total signs	≥5	27	Finafloxacin & Doxycycline	40	Diluent Control	15	46.872846	1	7.574496E-12	46.872846	1	7.574496E-12	1.000000	S*, A*
3	Average signs	≥5	27	Finafloxacin & Doxycycline	40	Diluent Control	15	46.872846	1	7.574496E-12	46.872846	1	7.574496E-12	1.000000	S*, A*
3	Total signs	≥5	28	Finafloxacin & Doxycycline	40	Diluent Control	15	46.872846	1	7.574496E-12	46.872846	1	7.574496E-12	1.000000	S*, A*
3	Average signs	≥5	28	Finafloxacin & Doxycycline	40	Diluent Control	15	46.872846	1	7.574496E-12	46.872846	1	7.574496E-12	1.000000	S*, A*
3	Total signs	≥5	29	Finafloxacin & Doxycycline	40	Diluent Control	15	46.872846	1	7.574496E-12	46.872846	1	7.574496E-12	1.000000	S*, A*
3	Average signs	≥5	29	Finafloxacin & Doxycycline	40	Diluent Control	15	46.872846	1	7.574496E-12	46.872846	1	7.574496E-12	1.000000	S*, A*
3	Total signs	≥5	30	Finafloxacin & Doxycycline	40	Diluent Control	15	46.872846	1	7.574496E-12	46.872846	1	7.574496E-12	1.000000	S*, A*
3	Average signs	≥5	30	Finafloxacin & Doxycycline	40	Diluent Control	15	46.872846	1	7.574496E-12	46.872846	1	7.574496E-12	1.000000	S*, A*
3	Total signs	≥5	20	Finafloxacin & Doxycycline	40	Finafloxacin	40	0.511166	1	4.746345E-01	0.709539	1	3.995968E-01	1.187784	S, A
3	Average signs	≥5	20	Finafloxacin & Doxycycline	40	Finafloxacin	40	1.010082	1	3.148832E-01	0.709539	1	3.995968E-01	0.788002	S, A
3	Total signs	≥5	21	Finafloxacin & Doxycycline	40	Finafloxacin	40	0.519836	1	4.709118E-01	0.709539	1	3.995968E-01	1.178468	S, A
3	Average signs	≥5	21	Finafloxacin & Doxycycline	40	Finafloxacin	40	1.022745	1	3.118688E-01	0.709539	1	3.995968E-01	0.780459	S, A
3	Total signs	≥5	22	Finafloxacin & Doxycycline	40	Finafloxacin	40	0.507920	1	4.760405E-01	0.709539	1	3.995968E-01	1.191302	S, A
3	Average signs	≥5	22	Finafloxacin & Doxycycline	40	Finafloxacin	40	1.004097	1	3.163213E-01	0.709539	1	3.995968E-01	0.791601	S, A
3	Total signs	≥5	23	Finafloxacin & Doxycycline	40	Finafloxacin	40	0.193853	1	6.597294E-01	0.709539	1	3.995968E-01	1.650988	S, A
3	Average signs	≥5	23	Finafloxacin & Doxycycline	40	Finafloxacin	40	0.543344	1	4.610503E-01	0.709539	1	3.995968E-01	1.153789	S, A
3	Total signs	≥5	24	Finafloxacin & Doxycycline	40	Finafloxacin	40	0.517801	1	4.717814E-01	0.709539	1	3.995968E-01	1.180644	S, A
3	Average signs	≥5	24	Finafloxacin & Doxycycline	40	Finafloxacin	40	1.087494	1	2.970271E-01	0.709539	1	3.995968E-01	0.743317	S, A
3	Total signs	≥5	25	Finafloxacin & Doxycycline	40	Finafloxacin	40	0.526837	1	4.679396E-01	0.709539	1	3.995968E-01	1.171029	S, A
3	Average signs	≥5	25	Finafloxacin & Doxycycline	40	Finafloxacin	40	1.101193	1	2.940044E-01	0.709539	1	3.995968E-01	0.735753	S, A

Study	Threshold Metric	Signs threshold	Weight threshold	Comparison Group A		Comparison Group B		Alternative endpoint Comparison			Original study comparison			P-value ratio	P-value ratio change
				Treatment	n	Treatment	n	Test statistic	df	p-value	Test statistic	df	p-value		
3	Total signs	≥5	26	Finafloxacin & Doxycycline	40	Finafloxacin	40	0.156492	1	6.924069E-01	0.709539	1	3.995968E-01	1.732764	S, A
3	Average signs	≥5	26	Finafloxacin & Doxycycline	40	Finafloxacin	40	0.527210	1	4.677824E-01	0.709539	1	3.995968E-01	1.170636	S, A
3	Total signs	≥5	27	Finafloxacin & Doxycycline	40	Finafloxacin	40	0.494527	1	4.819150E-01	0.709539	1	3.995968E-01	1.206003	S, A
3	Average signs	≥5	27	Finafloxacin & Doxycycline	40	Finafloxacin	40	1.142105	1	2.852081E-01	0.709539	1	3.995968E-01	0.713740	S, A
3	Total signs	≥5	28	Finafloxacin & Doxycycline	40	Finafloxacin	40	0.578893	1	4.467466E-01	0.709539	1	3.995968E-01	1.117993	S, A
3	Average signs	≥5	28	Finafloxacin & Doxycycline	40	Finafloxacin	40	1.403252	1	2.361798E-01	0.709539	1	3.995968E-01	0.591045	S, A
3	Total signs	≥5	29	Finafloxacin & Doxycycline	40	Finafloxacin	40	0.578893	1	4.467466E-01	0.709539	1	3.995968E-01	1.117993	S, A
3	Average signs	≥5	29	Finafloxacin & Doxycycline	40	Finafloxacin	40	1.403252	1	2.361798E-01	0.709539	1	3.995968E-01	0.591045	S, A
3	Total signs	≥5	30	Finafloxacin & Doxycycline	40	Finafloxacin	40	0.161476	1	6.878010E-01	0.709539	1	3.995968E-01	1.721238	S, A
3	Average signs	≥5	30	Finafloxacin & Doxycycline	40	Finafloxacin	40	0.709539	1	3.995968E-01	0.709539	1	3.995968E-01	1.000000	S, A
3	Total signs	≥5	20	Finafloxacin & Doxycycline	40	Doxycycline	40	5.247107	1	2.198329E-02	1.597411	1	2.062705E-01	0.106575	S, A*
3	Average signs	≥5	20	Finafloxacin & Doxycycline	40	Doxycycline	40	2.870004	1	9.024466E-02	1.597411	1	2.062705E-01	0.437506	S, A
3	Total signs	≥5	21	Finafloxacin & Doxycycline	40	Doxycycline	40	4.189132	1	4.068392E-02	1.597411	1	2.062705E-01	0.197236	S, A*
3	Average signs	≥5	21	Finafloxacin & Doxycycline	40	Doxycycline	40	2.092471	1	1.480265E-01	1.597411	1	2.062705E-01	0.717633	S, A
3	Total signs	≥5	22	Finafloxacin & Doxycycline	40	Doxycycline	40	4.197281	1	4.048885E-02	1.597411	1	2.062705E-01	0.196290	S, A*
3	Average signs	≥5	22	Finafloxacin & Doxycycline	40	Doxycycline	40	2.071806	1	1.500437E-01	1.597411	1	2.062705E-01	0.727412	S, A
3	Total signs	≥5	23	Finafloxacin & Doxycycline	40	Doxycycline	40	3.813253	1	5.084860E-02	1.597411	1	2.062705E-01	0.246514	S, A
3	Average signs	≥5	23	Finafloxacin & Doxycycline	40	Doxycycline	40	1.663650	1	1.971112E-01	1.597411	1	2.062705E-01	0.955596	S, A
3	Total signs	≥5	24	Finafloxacin & Doxycycline	40	Doxycycline	40	3.731715	1	5.338856E-02	1.597411	1	2.062705E-01	0.258828	S, A
3	Average signs	≥5	24	Finafloxacin & Doxycycline	40	Doxycycline	40	1.608566	1	2.046935E-01	1.597411	1	2.062705E-01	0.992355	S, A
3	Total signs	≥5	25	Finafloxacin & Doxycycline	40	Doxycycline	40	2.901569	1	8.849335E-02	1.597411	1	2.062705E-01	0.429016	S, A
3	Average signs	≥5	25	Finafloxacin & Doxycycline	40	Doxycycline	40	1.112079	1	2.916305E-01	1.597411	1	2.062705E-01	1.413826	S, A
3	Total signs	≥5	26	Finafloxacin & Doxycycline	40	Doxycycline	40	3.375862	1	6.615797E-02	1.597411	1	2.062705E-01	0.320734	S, A
3	Average signs	≥5	26	Finafloxacin & Doxycycline	40	Doxycycline	40	1.333774	1	2.481350E-01	1.597411	1	2.062705E-01	1.202959	S, A
3	Total signs	≥5	27	Finafloxacin & Doxycycline	40	Doxycycline	40	2.705162	1	1.000239E-01	1.597411	1	2.062705E-01	0.484916	S, A
3	Average signs	≥5	27	Finafloxacin & Doxycycline	40	Doxycycline	40	0.823965	1	3.640236E-01	1.597411	1	2.062705E-01	1.764787	S, A

Study	Threshold Metric	Signs threshold	Weight threshold	Comparison Group A		Comparison Group B		Alternative endpoint Comparison			Original study comparison			P-value ratio	P-value ratio change
				Treatment	n	Treatment	n	Test statistic	df	p-value	Test statistic	df	p-value		
3	Total signs	≥5	28	Finafloxacin & Doxycycline	40	Doxycycline	40	3.965698	1	4.643628E-02	1.597411	1	2.062705E-01	0.225123	S, A*
3	Average signs	≥5	28	Finafloxacin & Doxycycline	40	Doxycycline	40	1.561802	1	2.114016E-01	1.597411	1	2.062705E-01	1.024875	S, A
3	Total signs	≥5	29	Finafloxacin & Doxycycline	40	Doxycycline	40	3.965698	1	4.643628E-02	1.597411	1	2.062705E-01	0.225123	S, A*
3	Average signs	≥5	29	Finafloxacin & Doxycycline	40	Doxycycline	40	1.561802	1	2.114016E-01	1.597411	1	2.062705E-01	1.024875	S, A
3	Total signs	≥5	30	Finafloxacin & Doxycycline	40	Doxycycline	40	5.440163	1	1.967883E-02	1.597411	1	2.062705E-01	0.095403	S, A*
3	Average signs	≥5	30	Finafloxacin & Doxycycline	40	Doxycycline	40	2.562879	1	1.093992E-01	1.597411	1	2.062705E-01	0.530368	S, A
4	Total signs	≥5	20	Finafloxacin 36 h	45	Finafloxacin 48 h	44	0.563247	1	4.529551E-01	1.730962	1	1.882881E-01	2.405649	S, A
4	Average signs	≥5	20	Finafloxacin 36 h	45	Finafloxacin 48 h	44	0.445966	1	5.042569E-01	1.730962	1	1.882881E-01	2.678114	S, A
4	Total signs	≥5	21	Finafloxacin 36 h	45	Finafloxacin 48 h	44	0.321268	1	5.708468E-01	1.730962	1	1.882881E-01	3.031774	S, A
4	Average signs	≥5	21	Finafloxacin 36 h	45	Finafloxacin 48 h	44	0.235782	1	6.272695E-01	1.730962	1	1.882881E-01	3.331435	S, A
4	Total signs	≥5	22	Finafloxacin 36 h	45	Finafloxacin 48 h	44	0.325051	1	5.685880E-01	1.730962	1	1.882881E-01	3.019777	S, A
4	Average signs	≥5	22	Finafloxacin 36 h	45	Finafloxacin 48 h	44	0.454321	1	5.002902E-01	1.730962	1	1.882881E-01	2.657047	S, A
4	Total signs	≥5	23	Finafloxacin 36 h	45	Finafloxacin 48 h	44	0.113019	1	7.367325E-01	1.730962	1	1.882881E-01	3.912794	S, A
4	Average signs	≥5	23	Finafloxacin 36 h	45	Finafloxacin 48 h	44	0.211535	1	6.455672E-01	1.730962	1	1.882881E-01	3.428615	S, A
4	Total signs	≥5	24	Finafloxacin 36 h	45	Finafloxacin 48 h	44	0.071976	1	7.884806E-01	1.730962	1	1.882881E-01	4.187629	S, A
4	Average signs	≥5	24	Finafloxacin 36 h	45	Finafloxacin 48 h	44	0.570395	1	4.501019E-01	1.730962	1	1.882881E-01	2.390496	S, A
4	Total signs	≥5	25	Finafloxacin 36 h	45	Finafloxacin 48 h	44	0.246354	1	6.196540E-01	1.730962	1	1.882881E-01	3.290989	S, A
4	Average signs	≥5	25	Finafloxacin 36 h	45	Finafloxacin 48 h	44	0.960632	1	3.270278E-01	1.730962	1	1.882881E-01	1.736848	S, A
4	Total signs	≥5	26	Finafloxacin 36 h	45	Finafloxacin 48 h	44	0.226556	1	6.340896E-01	1.730962	1	1.882881E-01	3.367657	S, A
4	Average signs	≥5	26	Finafloxacin 36 h	45	Finafloxacin 48 h	44	0.971426	1	3.243248E-01	1.730962	1	1.882881E-01	1.722493	S, A
4	Total signs	≥5	27	Finafloxacin 36 h	45	Finafloxacin 48 h	44	0.223035	1	6.367368E-01	1.730962	1	1.882881E-01	3.381716	S, A
4	Average signs	≥5	27	Finafloxacin 36 h	45	Finafloxacin 48 h	44	0.949191	1	3.299252E-01	1.730962	1	1.882881E-01	1.752236	S, A
4	Total signs	≥5	28	Finafloxacin 36 h	45	Finafloxacin 48 h	44	0.392689	1	5.308895E-01	1.730962	1	1.882881E-01	2.819560	S, A
4	Average signs	≥5	28	Finafloxacin 36 h	45	Finafloxacin 48 h	44	1.317932	1	2.509636E-01	1.730962	1	1.882881E-01	1.332870	S, A
4	Total signs	≥5	29	Finafloxacin 36 h	45	Finafloxacin 48 h	44	0.376433	1	5.395184E-01	1.730962	1	1.882881E-01	2.865388	S, A
4	Average signs	≥5	29	Finafloxacin 36 h	45	Finafloxacin 48 h	44	1.840847	1	1.748517E-01	1.730962	1	1.882881E-01	0.928639	S, A
4	Total signs	≥5	30	Finafloxacin 36 h	45	Finafloxacin 48 h	44	0.347491	1	5.555367E-01	1.730962	1	1.882881E-01	2.950462	S, A
4	Average signs	≥5	30	Finafloxacin 36 h	45	Finafloxacin 48 h	44	1.812049	1	1.782626E-01	1.730962	1	1.882881E-01	0.946755	S, A
4	Total signs	≥5	20	Finafloxacin 36 h	45	Diluent Control	88	94.766888	1	2.141784E-22	94.766888	1	2.141784E-22	1.000000	S*, A*
4	Average signs	≥5	20	Finafloxacin 36 h	45	Diluent Control	88	94.766888	1	2.141784E-22	94.766888	1	2.141784E-22	1.000000	S*, A*
4	Total signs	≥5	21	Finafloxacin 36 h	45	Diluent Control	88	94.766888	1	2.141784E-22	94.766888	1	2.141784E-22	1.000000	S*, A*
4	Average signs	≥5	21	Finafloxacin 36 h	45	Diluent Control	88	94.766888	1	2.141784E-22	94.766888	1	2.141784E-22	1.000000	S*, A*
4	Total signs	≥5	22	Finafloxacin 36 h	45	Diluent Control	88	94.766888	1	2.141784E-22	94.766888	1	2.141784E-22	1.000000	S*, A*
4	Average signs	≥5	22	Finafloxacin 36 h	45	Diluent Control	88	94.766888	1	2.141784E-22	94.766888	1	2.141784E-22	1.000000	S*, A*
4	Total signs	≥5	23	Finafloxacin 36 h	45	Diluent Control	88	94.766888	1	2.141784E-22	94.766888	1	2.141784E-22	1.000000	S*, A*

Study	Threshold Metric	Signs threshold	Weight threshold	Comparison Group A		Comparison Group B		Alternative endpoint Comparison			Original study comparison			P-value ratio	P-value ratio change
				Treatment	n	Treatment	n	Test statistic	df	p-value	Test statistic	df	p-value		
4	Average signs	≥5	23	Finafloxacin 36 h	45	Diluent Control	88	94.766888	1	2.141784E-22	94.766888	1	2.141784E-22	1.000000	S*, A*
4	Total signs	≥5	24	Finafloxacin 36 h	45	Diluent Control	88	94.766888	1	2.141784E-22	94.766888	1	2.141784E-22	1.000000	S*, A*
4	Average signs	≥5	24	Finafloxacin 36 h	45	Diluent Control	88	94.766888	1	2.141784E-22	94.766888	1	2.141784E-22	1.000000	S*, A*
4	Total signs	≥5	25	Finafloxacin 36 h	45	Diluent Control	88	94.766888	1	2.141784E-22	94.766888	1	2.141784E-22	1.000000	S*, A*
4	Average signs	≥5	25	Finafloxacin 36 h	45	Diluent Control	88	94.766888	1	2.141784E-22	94.766888	1	2.141784E-22	1.000000	S*, A*
4	Total signs	≥5	26	Finafloxacin 36 h	45	Diluent Control	88	94.766888	1	2.141784E-22	94.766888	1	2.141784E-22	1.000000	S*, A*
4	Average signs	≥5	26	Finafloxacin 36 h	45	Diluent Control	88	94.766888	1	2.141784E-22	94.766888	1	2.141784E-22	1.000000	S*, A*
4	Total signs	≥5	27	Finafloxacin 36 h	45	Diluent Control	88	94.766888	1	2.141784E-22	94.766888	1	2.141784E-22	1.000000	S*, A*
4	Average signs	≥5	27	Finafloxacin 36 h	45	Diluent Control	88	94.766888	1	2.141784E-22	94.766888	1	2.141784E-22	1.000000	S*, A*
4	Total signs	≥5	28	Finafloxacin 36 h	45	Diluent Control	88	94.766888	1	2.141784E-22	94.766888	1	2.141784E-22	1.000000	S*, A*
4	Average signs	≥5	28	Finafloxacin 36 h	45	Diluent Control	88	94.766888	1	2.141784E-22	94.766888	1	2.141784E-22	1.000000	S*, A*
4	Total signs	≥5	29	Finafloxacin 36 h	45	Diluent Control	88	94.766888	1	2.141784E-22	94.766888	1	2.141784E-22	1.000000	S*, A*
4	Average signs	≥5	29	Finafloxacin 36 h	45	Diluent Control	88	94.766888	1	2.141784E-22	94.766888	1	2.141784E-22	1.000000	S*, A*
4	Total signs	≥5	30	Finafloxacin 36 h	45	Diluent Control	88	94.766888	1	2.141784E-22	94.766888	1	2.141784E-22	1.000000	S*, A*
4	Average signs	≥5	30	Finafloxacin 36 h	45	Diluent Control	88	94.766888	1	2.141784E-22	94.766888	1	2.141784E-22	1.000000	S*, A*
4	Total signs	≥5	20	Finafloxacin 48 h	44	Diluent Control	88	72.966845	1	1.318462E-17	72.966845	1	1.318462E-17	1.000000	S*, A*
4	Average signs	≥5	20	Finafloxacin 48 h	44	Diluent Control	88	72.966845	1	1.318462E-17	72.966845	1	1.318462E-17	1.000000	S*, A*
4	Total signs	≥5	21	Finafloxacin 48 h	44	Diluent Control	88	72.966845	1	1.318462E-17	72.966845	1	1.318462E-17	1.000000	S*, A*
4	Average signs	≥5	21	Finafloxacin 48 h	44	Diluent Control	88	72.966845	1	1.318462E-17	72.966845	1	1.318462E-17	1.000000	S*, A*
4	Total signs	≥5	22	Finafloxacin 48 h	44	Diluent Control	88	72.966845	1	1.318462E-17	72.966845	1	1.318462E-17	1.000000	S*, A*
4	Average signs	≥5	22	Finafloxacin 48 h	44	Diluent Control	88	72.966845	1	1.318462E-17	72.966845	1	1.318462E-17	1.000000	S*, A*
4	Total signs	≥5	23	Finafloxacin 48 h	44	Diluent Control	88	72.966845	1	1.318462E-17	72.966845	1	1.318462E-17	1.000000	S*, A*
4	Average signs	≥5	23	Finafloxacin 48 h	44	Diluent Control	88	72.966845	1	1.318462E-17	72.966845	1	1.318462E-17	1.000000	S*, A*
4	Total signs	≥5	24	Finafloxacin 48 h	44	Diluent Control	88	72.966845	1	1.318462E-17	72.966845	1	1.318462E-17	1.000000	S*, A*
4	Average signs	≥5	24	Finafloxacin 48 h	44	Diluent Control	88	72.966845	1	1.318462E-17	72.966845	1	1.318462E-17	1.000000	S*, A*
4	Total signs	≥5	25	Finafloxacin 48 h	44	Diluent Control	88	72.966845	1	1.318462E-17	72.966845	1	1.318462E-17	1.000000	S*, A*
4	Average signs	≥5	25	Finafloxacin 48 h	44	Diluent Control	88	72.966845	1	1.318462E-17	72.966845	1	1.318462E-17	1.000000	S*, A*
4	Total signs	≥5	26	Finafloxacin 48 h	44	Diluent Control	88	72.966845	1	1.318462E-17	72.966845	1	1.318462E-17	1.000000	S*, A*
4	Average signs	≥5	26	Finafloxacin 48 h	44	Diluent Control	88	72.966845	1	1.318462E-17	72.966845	1	1.318462E-17	1.000000	S*, A*
4	Total signs	≥5	27	Finafloxacin 48 h	44	Diluent Control	88	72.966845	1	1.318462E-17	72.966845	1	1.318462E-17	1.000000	S*, A*
4	Average signs	≥5	27	Finafloxacin 48 h	44	Diluent Control	88	72.966845	1	1.318462E-17	72.966845	1	1.318462E-17	1.000000	S*, A*
4	Total signs	≥5	28	Finafloxacin 48 h	44	Diluent Control	88	72.966845	1	1.318462E-17	72.966845	1	1.318462E-17	1.000000	S*, A*
4	Average signs	≥5	28	Finafloxacin 48 h	44	Diluent Control	88	72.966845	1	1.318462E-17	72.966845	1	1.318462E-17	1.000000	S*, A*
4	Total signs	≥5	29	Finafloxacin 48 h	44	Diluent Control	88	72.966845	1	1.318462E-17	72.966845	1	1.318462E-17	1.000000	S*, A*
4	Average signs	≥5	29	Finafloxacin 48 h	44	Diluent Control	88	72.966845	1	1.318462E-17	72.966845	1	1.318462E-17	1.000000	S*, A*
4	Total signs	≥5	30	Finafloxacin 48 h	44	Diluent Control	88	72.966845	1	1.318462E-17	72.966845	1	1.318462E-17	1.000000	S*, A*
4	Average signs	≥5	30	Finafloxacin 48 h	44	Diluent Control	88	72.966845	1	1.318462E-17	72.966845	1	1.318462E-17	1.000000	S*, A*
1	Total signs	≥6	20	Finafloxacin	45	Diluent Control	15	83.192904	1	7.442441E-20	83.192904	1	7.442441E-20	1.000000	S*, A*

Study	Threshold Metric	Signs threshold	Weight threshold	Comparison Group A		Comparison Group B		Alternative endpoint Comparison			Original study comparison			P-value ratio	P-value ratio change
				Treatment	n	Treatment	n	Test statistic	df	p-value	Test statistic	df	p-value		
1	Average signs	≥6	20	Finafloxacin	45	Diluent Control	15	83.192904	1	7.442441E-20	83.192904	1	7.442441E-20	1.000000	S*, A*
1	Total signs	≥6	21	Finafloxacin	45	Diluent Control	15	83.192904	1	7.442441E-20	83.192904	1	7.442441E-20	1.000000	S*, A*
1	Average signs	≥6	21	Finafloxacin	45	Diluent Control	15	83.192904	1	7.442441E-20	83.192904	1	7.442441E-20	1.000000	S*, A*
1	Total signs	≥6	22	Finafloxacin	45	Diluent Control	15	83.192904	1	7.442441E-20	83.192904	1	7.442441E-20	1.000000	S*, A*
1	Average signs	≥6	22	Finafloxacin	45	Diluent Control	15	83.192904	1	7.442441E-20	83.192904	1	7.442441E-20	1.000000	S*, A*
1	Total signs	≥6	23	Finafloxacin	45	Diluent Control	15	83.192904	1	7.442441E-20	83.192904	1	7.442441E-20	1.000000	S*, A*
1	Average signs	≥6	23	Finafloxacin	45	Diluent Control	15	83.192904	1	7.442441E-20	83.192904	1	7.442441E-20	1.000000	S*, A*
1	Total signs	≥6	24	Finafloxacin	45	Diluent Control	15	83.192904	1	7.442441E-20	83.192904	1	7.442441E-20	1.000000	S*, A*
1	Average signs	≥6	24	Finafloxacin	45	Diluent Control	15	83.192904	1	7.442441E-20	83.192904	1	7.442441E-20	1.000000	S*, A*
1	Total signs	≥6	25	Finafloxacin	45	Diluent Control	15	83.192904	1	7.442441E-20	83.192904	1	7.442441E-20	1.000000	S*, A*
1	Average signs	≥6	25	Finafloxacin	45	Diluent Control	15	83.192904	1	7.442441E-20	83.192904	1	7.442441E-20	1.000000	S*, A*
1	Total signs	≥6	26	Finafloxacin	45	Diluent Control	15	83.192904	1	7.442441E-20	83.192904	1	7.442441E-20	1.000000	S*, A*
1	Average signs	≥6	26	Finafloxacin	45	Diluent Control	15	83.192904	1	7.442441E-20	83.192904	1	7.442441E-20	1.000000	S*, A*
1	Total signs	≥6	27	Finafloxacin	45	Diluent Control	15	83.192904	1	7.442441E-20	83.192904	1	7.442441E-20	1.000000	S*, A*
1	Average signs	≥6	27	Finafloxacin	45	Diluent Control	15	83.192904	1	7.442441E-20	83.192904	1	7.442441E-20	1.000000	S*, A*
1	Total signs	≥6	28	Finafloxacin	45	Diluent Control	15	83.192904	1	7.442441E-20	83.192904	1	7.442441E-20	1.000000	S*, A*
1	Average signs	≥6	28	Finafloxacin	45	Diluent Control	15	83.192904	1	7.442441E-20	83.192904	1	7.442441E-20	1.000000	S*, A*
1	Total signs	≥6	29	Finafloxacin	45	Diluent Control	15	83.192904	1	7.442441E-20	83.192904	1	7.442441E-20	1.000000	S*, A*
1	Average signs	≥6	29	Finafloxacin	45	Diluent Control	15	83.192904	1	7.442441E-20	83.192904	1	7.442441E-20	1.000000	S*, A*
1	Total signs	≥6	30	Finafloxacin	45	Diluent Control	15	83.192904	1	7.442441E-20	83.192904	1	7.442441E-20	1.000000	S*, A*
1	Average signs	≥6	30	Finafloxacin	45	Diluent Control	15	83.192904	1	7.442441E-20	83.192904	1	7.442441E-20	1.000000	S*, A*
1	Total signs	≥6	20	Co-trimoxazole	45	Diluent Control	15	83.192904	1	7.442441E-20	83.192904	1	7.442441E-20	1.000000	S*, A*
1	Average signs	≥6	20	Co-trimoxazole	45	Diluent Control	15	83.192904	1	7.442441E-20	83.192904	1	7.442441E-20	1.000000	S*, A*
1	Total signs	≥6	21	Co-trimoxazole	45	Diluent Control	15	83.192904	1	7.442441E-20	83.192904	1	7.442441E-20	1.000000	S*, A*
1	Average signs	≥6	21	Co-trimoxazole	45	Diluent Control	15	83.192904	1	7.442441E-20	83.192904	1	7.442441E-20	1.000000	S*, A*
1	Total signs	≥6	22	Co-trimoxazole	45	Diluent Control	15	83.192904	1	7.442441E-20	83.192904	1	7.442441E-20	1.000000	S*, A*
1	Average signs	≥6	22	Co-trimoxazole	45	Diluent Control	15	83.192904	1	7.442441E-20	83.192904	1	7.442441E-20	1.000000	S*, A*
1	Total signs	≥6	23	Co-trimoxazole	45	Diluent Control	15	83.192904	1	7.442441E-20	83.192904	1	7.442441E-20	1.000000	S*, A*
1	Average signs	≥6	23	Co-trimoxazole	45	Diluent Control	15	83.192904	1	7.442441E-20	83.192904	1	7.442441E-20	1.000000	S*, A*
1	Total signs	≥6	24	Co-trimoxazole	45	Diluent Control	15	83.192904	1	7.442441E-20	83.192904	1	7.442441E-20	1.000000	S*, A*
1	Average signs	≥6	24	Co-trimoxazole	45	Diluent Control	15	83.192904	1	7.442441E-20	83.192904	1	7.442441E-20	1.000000	S*, A*
1	Total signs	≥6	25	Co-trimoxazole	45	Diluent Control	15	83.192904	1	7.442441E-20	83.192904	1	7.442441E-20	1.000000	S*, A*
1	Average signs	≥6	25	Co-trimoxazole	45	Diluent Control	15	83.192904	1	7.442441E-20	83.192904	1	7.442441E-20	1.000000	S*, A*
1	Total signs	≥6	26	Co-trimoxazole	45	Diluent Control	15	83.192904	1	7.442441E-20	83.192904	1	7.442441E-20	1.000000	S*, A*
1	Average signs	≥6	26	Co-trimoxazole	45	Diluent Control	15	83.192904	1	7.442441E-20	83.192904	1	7.442441E-20	1.000000	S*, A*
1	Total signs	≥6	27	Co-trimoxazole	45	Diluent Control	15	83.192904	1	7.442441E-20	83.192904	1	7.442441E-20	1.000000	S*, A*
1	Average signs	≥6	27	Co-trimoxazole	45	Diluent Control	15	83.192904	1	7.442441E-20	83.192904	1	7.442441E-20	1.000000	S*, A*
1	Total signs	≥6	28	Co-trimoxazole	45	Diluent Control	15	83.192904	1	7.442441E-20	83.192904	1	7.442441E-20	1.000000	S*, A*

Study	Threshold Metric	Signs threshold	Weight threshold	Comparison Group A		Comparison Group B		Alternative endpoint Comparison			Original study comparison			P-value ratio	P-value ratio change
				Treatment	n	Treatment	n	Test statistic	df	p-value	Test statistic	df	p-value		
1	Average signs	≥6	28	Co-trimoxazole	45	Diluent Control	15	83.192904	1	7.442441E-20	83.192904	1	7.442441E-20	1.000000	S*, A*
1	Total signs	≥6	29	Co-trimoxazole	45	Diluent Control	15	83.192904	1	7.442441E-20	83.192904	1	7.442441E-20	1.000000	S*, A*
1	Average signs	≥6	29	Co-trimoxazole	45	Diluent Control	15	83.192904	1	7.442441E-20	83.192904	1	7.442441E-20	1.000000	S*, A*
1	Total signs	≥6	30	Co-trimoxazole	45	Diluent Control	15	83.192904	1	7.442441E-20	83.192904	1	7.442441E-20	1.000000	S*, A*
1	Average signs	≥6	30	Co-trimoxazole	45	Diluent Control	15	83.192904	1	7.442441E-20	83.192904	1	7.442441E-20	1.000000	S*, A*
1	Total signs	≥6	20	Co-trimoxazole	45	Finafloxacin	45	2.327824	1	1.270794E-01	11.725428	1	6.165186E-04	206.124156	S*, A
1	Average signs	≥6	20	Co-trimoxazole	45	Finafloxacin	45	1.639487	1	2.003959E-01	11.725428	1	6.165186E-04	325.044283	S*, A
1	Total signs	≥6	21	Co-trimoxazole	45	Finafloxacin	45	4.572057	1	3.249752E-02	11.725428	1	6.165186E-04	52.711339	S*, A*
1	Average signs	≥6	21	Co-trimoxazole	45	Finafloxacin	45	3.947069	1	4.695309E-02	11.725428	1	6.165186E-04	76.158434	S*, A*
1	Total signs	≥6	22	Co-trimoxazole	45	Finafloxacin	45	6.987639	1	8.207453E-03	11.725428	1	6.165186E-04	13.312580	S*, A*
1	Average signs	≥6	22	Co-trimoxazole	45	Finafloxacin	45	6.085769	1	1.362750E-02	11.725428	1	6.165186E-04	22.103962	S*, A*
1	Total signs	≥6	23	Co-trimoxazole	45	Finafloxacin	45	11.019074	1	9.017909E-04	11.725428	1	6.165186E-04	1.462715	S*, A*
1	Average signs	≥6	23	Co-trimoxazole	45	Finafloxacin	45	10.022255	1	1.546600E-03	11.725428	1	6.165186E-04	2.508603	S*, A*
1	Total signs	≥6	24	Co-trimoxazole	45	Finafloxacin	45	10.315288	1	1.319327E-03	11.725428	1	6.165186E-04	2.139963	S*, A*
1	Average signs	≥6	24	Co-trimoxazole	45	Finafloxacin	45	8.576220	1	3.405817E-03	11.725428	1	6.165186E-04	5.524273	S*, A*
1	Total signs	≥6	25	Co-trimoxazole	45	Finafloxacin	45	10.398444	1	1.261216E-03	11.725428	1	6.165186E-04	2.045705	S*, A*
1	Average signs	≥6	25	Co-trimoxazole	45	Finafloxacin	45	8.715796	1	3.154646E-03	11.725428	1	6.165186E-04	5.116870	S*, A*
1	Total signs	≥6	26	Co-trimoxazole	45	Finafloxacin	45	10.494412	1	1.197361E-03	11.725428	1	6.165186E-04	1.942133	S*, A*
1	Average signs	≥6	26	Co-trimoxazole	45	Finafloxacin	45	8.830296	1	2.962703E-03	11.725428	1	6.165186E-04	4.805537	S*, A*
1	Total signs	≥6	27	Co-trimoxazole	45	Finafloxacin	45	13.059523	1	3.017463E-04	11.725428	1	6.165186E-04	0.489436	S*, A*
1	Average signs	≥6	27	Co-trimoxazole	45	Finafloxacin	45	12.089904	1	5.069558E-04	11.725428	1	6.165186E-04	0.822288	S*, A*
1	Total signs	≥6	28	Co-trimoxazole	45	Finafloxacin	45	14.767029	1	1.216439E-04	11.725428	1	6.165186E-04	0.197308	S*, A*
1	Average signs	≥6	28	Co-trimoxazole	45	Finafloxacin	45	14.266281	1	1.586822E-04	11.725428	1	6.165186E-04	0.257384	S*, A*
1	Total signs	≥6	29	Co-trimoxazole	45	Finafloxacin	45	14.970072	1	1.092299E-04	11.725428	1	6.165186E-04	0.177172	S*, A*
1	Average signs	≥6	29	Co-trimoxazole	45	Finafloxacin	45	14.260189	1	1.591966E-04	11.725428	1	6.165186E-04	0.258219	S*, A*
1	Total signs	≥6	30	Co-trimoxazole	45	Finafloxacin	45	14.970072	1	1.092299E-04	11.725428	1	6.165186E-04	0.177172	S*, A*
1	Average signs	≥6	30	Co-trimoxazole	45	Finafloxacin	45	14.369219	1	1.502383E-04	11.725428	1	6.165186E-04	0.243688	S*, A*
2	Total signs	≥6	20	Finafloxacin	90	Diluent Control	20	136.184833	1	1.817952E-31	136.184833	1	1.817952E-31	1.000000	S*, A*
2	Average signs	≥6	20	Finafloxacin	90	Diluent Control	20	136.184833	1	1.817952E-31	136.184833	1	1.817952E-31	1.000000	S*, A*
2	Total signs	≥6	21	Finafloxacin	90	Diluent Control	20	136.184833	1	1.817952E-31	136.184833	1	1.817952E-31	1.000000	S*, A*
2	Average signs	≥6	21	Finafloxacin	90	Diluent Control	20	136.184833	1	1.817952E-31	136.184833	1	1.817952E-31	1.000000	S*, A*
2	Total signs	≥6	22	Finafloxacin	90	Diluent Control	20	136.184833	1	1.817952E-31	136.184833	1	1.817952E-31	1.000000	S*, A*
2	Average signs	≥6	22	Finafloxacin	90	Diluent Control	20	136.184833	1	1.817952E-31	136.184833	1	1.817952E-31	1.000000	S*, A*
2	Total signs	≥6	23	Finafloxacin	90	Diluent Control	20	136.184833	1	1.817952E-31	136.184833	1	1.817952E-31	1.000000	S*, A*
2	Average signs	≥6	23	Finafloxacin	90	Diluent Control	20	136.184833	1	1.817952E-31	136.184833	1	1.817952E-31	1.000000	S*, A*
2	Total signs	≥6	24	Finafloxacin	90	Diluent Control	20	136.184833	1	1.817952E-31	136.184833	1	1.817952E-31	1.000000	S*, A*
2	Average signs	≥6	24	Finafloxacin	90	Diluent Control	20	136.184833	1	1.817952E-31	136.184833	1	1.817952E-31	1.000000	S*, A*
2	Total signs	≥6	25	Finafloxacin	90	Diluent Control	20	136.184833	1	1.817952E-31	136.184833	1	1.817952E-31	1.000000	S*, A*

Study	Threshold Metric	Signs threshold	Weight threshold	Comparison Group A		Comparison Group B		Alternative endpoint Comparison			Original study comparison			P-value ratio	P-value ratio change
				Treatment	n	Treatment	n	Test statistic	df	p-value	Test statistic	df	p-value		
2	Average signs	≥6	25	Finafloxacin	90	Diluent Control	20	136.184833	1	1.817952E-31	136.184833	1	1.817952E-31	1.000000	S*, A*
2	Total signs	≥6	26	Finafloxacin	90	Diluent Control	20	136.184833	1	1.817952E-31	136.184833	1	1.817952E-31	1.000000	S*, A*
2	Average signs	≥6	26	Finafloxacin	90	Diluent Control	20	136.184833	1	1.817952E-31	136.184833	1	1.817952E-31	1.000000	S*, A*
2	Total signs	≥6	27	Finafloxacin	90	Diluent Control	20	136.184833	1	1.817952E-31	136.184833	1	1.817952E-31	1.000000	S*, A*
2	Average signs	≥6	27	Finafloxacin	90	Diluent Control	20	136.184833	1	1.817952E-31	136.184833	1	1.817952E-31	1.000000	S*, A*
2	Total signs	≥6	28	Finafloxacin	90	Diluent Control	20	136.184833	1	1.817952E-31	136.184833	1	1.817952E-31	1.000000	S*, A*
2	Average signs	≥6	28	Finafloxacin	90	Diluent Control	20	136.184833	1	1.817952E-31	136.184833	1	1.817952E-31	1.000000	S*, A*
2	Total signs	≥6	29	Finafloxacin	90	Diluent Control	20	136.184833	1	1.817952E-31	136.184833	1	1.817952E-31	1.000000	S*, A*
2	Average signs	≥6	29	Finafloxacin	90	Diluent Control	20	136.184833	1	1.817952E-31	136.184833	1	1.817952E-31	1.000000	S*, A*
2	Total signs	≥6	30	Finafloxacin	90	Diluent Control	20	136.184833	1	1.817952E-31	136.184833	1	1.817952E-31	1.000000	S*, A*
2	Average signs	≥6	30	Finafloxacin	90	Diluent Control	20	136.184833	1	1.817952E-31	136.184833	1	1.817952E-31	1.000000	S*, A*
3	Total signs	≥6	20	Finafloxacin	40	Diluent Control	15	65.229390	1	6.666781E-16	65.229390	1	6.666781E-16	1.000000	S*, A*
3	Average signs	≥6	20	Finafloxacin	40	Diluent Control	15	65.229390	1	6.666781E-16	65.229390	1	6.666781E-16	1.000000	S*, A*
3	Total signs	≥6	21	Finafloxacin	40	Diluent Control	15	65.229390	1	6.666781E-16	65.229390	1	6.666781E-16	1.000000	S*, A*
3	Average signs	≥6	21	Finafloxacin	40	Diluent Control	15	65.229390	1	6.666781E-16	65.229390	1	6.666781E-16	1.000000	S*, A*
3	Total signs	≥6	22	Finafloxacin	40	Diluent Control	15	65.229390	1	6.666781E-16	65.229390	1	6.666781E-16	1.000000	S*, A*
3	Average signs	≥6	22	Finafloxacin	40	Diluent Control	15	65.229390	1	6.666781E-16	65.229390	1	6.666781E-16	1.000000	S*, A*
3	Total signs	≥6	23	Finafloxacin	40	Diluent Control	15	65.229390	1	6.666781E-16	65.229390	1	6.666781E-16	1.000000	S*, A*
3	Average signs	≥6	23	Finafloxacin	40	Diluent Control	15	65.229390	1	6.666781E-16	65.229390	1	6.666781E-16	1.000000	S*, A*
3	Total signs	≥6	24	Finafloxacin	40	Diluent Control	15	65.229390	1	6.666781E-16	65.229390	1	6.666781E-16	1.000000	S*, A*
3	Average signs	≥6	24	Finafloxacin	40	Diluent Control	15	65.229390	1	6.666781E-16	65.229390	1	6.666781E-16	1.000000	S*, A*
3	Total signs	≥6	25	Finafloxacin	40	Diluent Control	15	65.229390	1	6.666781E-16	65.229390	1	6.666781E-16	1.000000	S*, A*
3	Average signs	≥6	25	Finafloxacin	40	Diluent Control	15	65.229390	1	6.666781E-16	65.229390	1	6.666781E-16	1.000000	S*, A*
3	Total signs	≥6	26	Finafloxacin	40	Diluent Control	15	65.229390	1	6.666781E-16	65.229390	1	6.666781E-16	1.000000	S*, A*
3	Average signs	≥6	26	Finafloxacin	40	Diluent Control	15	65.229390	1	6.666781E-16	65.229390	1	6.666781E-16	1.000000	S*, A*
3	Total signs	≥6	27	Finafloxacin	40	Diluent Control	15	65.229390	1	6.666781E-16	65.229390	1	6.666781E-16	1.000000	S*, A*
3	Average signs	≥6	27	Finafloxacin	40	Diluent Control	15	65.229390	1	6.666781E-16	65.229390	1	6.666781E-16	1.000000	S*, A*
3	Total signs	≥6	28	Finafloxacin	40	Diluent Control	15	65.229390	1	6.666781E-16	65.229390	1	6.666781E-16	1.000000	S*, A*
3	Average signs	≥6	28	Finafloxacin	40	Diluent Control	15	65.229390	1	6.666781E-16	65.229390	1	6.666781E-16	1.000000	S*, A*
3	Total signs	≥6	29	Finafloxacin	40	Diluent Control	15	65.229390	1	6.666781E-16	65.229390	1	6.666781E-16	1.000000	S*, A*
3	Average signs	≥6	29	Finafloxacin	40	Diluent Control	15	65.229390	1	6.666781E-16	65.229390	1	6.666781E-16	1.000000	S*, A*
3	Total signs	≥6	30	Finafloxacin	40	Diluent Control	15	65.229390	1	6.666781E-16	65.229390	1	6.666781E-16	1.000000	S*, A*
3	Average signs	≥6	30	Finafloxacin	40	Diluent Control	15	65.229390	1	6.666781E-16	65.229390	1	6.666781E-16	1.000000	S*, A*
3	Total signs	≥6	20	Doxycycline	40	Diluent Control	15	65.229390	1	6.666781E-16	65.229390	1	6.666781E-16	1.000000	S*, A*
3	Average signs	≥6	20	Doxycycline	40	Diluent Control	15	65.229390	1	6.666781E-16	65.229390	1	6.666781E-16	1.000000	S*, A*
3	Total signs	≥6	21	Doxycycline	40	Diluent Control	15	65.229390	1	6.666781E-16	65.229390	1	6.666781E-16	1.000000	S*, A*
3	Average signs	≥6	21	Doxycycline	40	Diluent Control	15	65.229390	1	6.666781E-16	65.229390	1	6.666781E-16	1.000000	S*, A*
3	Total signs	≥6	22	Doxycycline	40	Diluent Control	15	65.229390	1	6.666781E-16	65.229390	1	6.666781E-16	1.000000	S*, A*

Study	Threshold Metric	Signs threshold	Weight threshold	Comparison Group A		Comparison Group B		Alternative endpoint Comparison			Original study comparison			P-value ratio	P-value ratio change
				Treatment	n	Treatment	n	Test statistic	df	p-value	Test statistic	df	p-value		
3	Average signs	≥6	22	Doxycycline	40	Diluent Control	15	65.229390	1	6.666781E-16	65.229390	1	6.666781E-16	1.000000	S*, A*
3	Total signs	≥6	23	Doxycycline	40	Diluent Control	15	65.229390	1	6.666781E-16	65.229390	1	6.666781E-16	1.000000	S*, A*
3	Average signs	≥6	23	Doxycycline	40	Diluent Control	15	65.229390	1	6.666781E-16	65.229390	1	6.666781E-16	1.000000	S*, A*
3	Total signs	≥6	24	Doxycycline	40	Diluent Control	15	65.229390	1	6.666781E-16	65.229390	1	6.666781E-16	1.000000	S*, A*
3	Average signs	≥6	24	Doxycycline	40	Diluent Control	15	65.229390	1	6.666781E-16	65.229390	1	6.666781E-16	1.000000	S*, A*
3	Total signs	≥6	25	Doxycycline	40	Diluent Control	15	65.229390	1	6.666781E-16	65.229390	1	6.666781E-16	1.000000	S*, A*
3	Average signs	≥6	25	Doxycycline	40	Diluent Control	15	65.229390	1	6.666781E-16	65.229390	1	6.666781E-16	1.000000	S*, A*
3	Total signs	≥6	26	Doxycycline	40	Diluent Control	15	65.229390	1	6.666781E-16	65.229390	1	6.666781E-16	1.000000	S*, A*
3	Average signs	≥6	26	Doxycycline	40	Diluent Control	15	65.229390	1	6.666781E-16	65.229390	1	6.666781E-16	1.000000	S*, A*
3	Total signs	≥6	27	Doxycycline	40	Diluent Control	15	65.229390	1	6.666781E-16	65.229390	1	6.666781E-16	1.000000	S*, A*
3	Average signs	≥6	27	Doxycycline	40	Diluent Control	15	65.229390	1	6.666781E-16	65.229390	1	6.666781E-16	1.000000	S*, A*
3	Total signs	≥6	28	Doxycycline	40	Diluent Control	15	65.229390	1	6.666781E-16	65.229390	1	6.666781E-16	1.000000	S*, A*
3	Average signs	≥6	28	Doxycycline	40	Diluent Control	15	65.229390	1	6.666781E-16	65.229390	1	6.666781E-16	1.000000	S*, A*
3	Total signs	≥6	29	Doxycycline	40	Diluent Control	15	65.229390	1	6.666781E-16	65.229390	1	6.666781E-16	1.000000	S*, A*
3	Average signs	≥6	29	Doxycycline	40	Diluent Control	15	65.229390	1	6.666781E-16	65.229390	1	6.666781E-16	1.000000	S*, A*
3	Total signs	≥6	30	Doxycycline	40	Diluent Control	15	65.229390	1	6.666781E-16	65.229390	1	6.666781E-16	1.000000	S*, A*
3	Average signs	≥6	30	Doxycycline	40	Diluent Control	15	65.229390	1	6.666781E-16	65.229390	1	6.666781E-16	1.000000	S*, A*
3	Total signs	≥6	20	Doxycycline	40	Finafloxacin	40	6.377765	1	1.155589E-02	4.326465	1	3.752411E-02	0.307959	S*, A*
3	Average signs	≥6	20	Doxycycline	40	Finafloxacin	40	6.377765	1	1.155589E-02	4.326465	1	3.752411E-02	0.307959	S*, A*
3	Total signs	≥6	21	Doxycycline	40	Finafloxacin	40	5.112743	1	2.375075E-02	4.326465	1	3.752411E-02	0.632946	S*, A*
3	Average signs	≥6	21	Doxycycline	40	Finafloxacin	40	5.112743	1	2.375075E-02	4.326465	1	3.752411E-02	0.632946	S*, A*
3	Total signs	≥6	22	Doxycycline	40	Finafloxacin	40	5.111622	1	2.376609E-02	4.326465	1	3.752411E-02	0.633355	S*, A*
3	Average signs	≥6	22	Doxycycline	40	Finafloxacin	40	5.111622	1	2.376609E-02	4.326465	1	3.752411E-02	0.633355	S*, A*
3	Total signs	≥6	23	Doxycycline	40	Finafloxacin	40	3.475977	1	6.226596E-02	4.326465	1	3.752411E-02	1.659359	S*, A
3	Average signs	≥6	23	Doxycycline	40	Finafloxacin	40	3.475977	1	6.226596E-02	4.326465	1	3.752411E-02	1.659359	S*, A
3	Total signs	≥6	24	Doxycycline	40	Finafloxacin	40	4.276748	1	3.863721E-02	4.326465	1	3.752411E-02	1.029664	S*, A*
3	Average signs	≥6	24	Doxycycline	40	Finafloxacin	40	4.276748	1	3.863721E-02	4.326465	1	3.752411E-02	1.029664	S*, A*
3	Total signs	≥6	25	Doxycycline	40	Finafloxacin	40	3.435375	1	6.381411E-02	4.326465	1	3.752411E-02	1.700616	S*, A
3	Average signs	≥6	25	Doxycycline	40	Finafloxacin	40	3.435375	1	6.381411E-02	4.326465	1	3.752411E-02	1.700616	S*, A
3	Total signs	≥6	26	Doxycycline	40	Finafloxacin	40	2.565054	1	1.092488E-01	4.326465	1	3.752411E-02	2.911430	S*, A
3	Average signs	≥6	26	Doxycycline	40	Finafloxacin	40	2.565054	1	1.092488E-01	4.326465	1	3.752411E-02	2.911430	S*, A
3	Total signs	≥6	27	Doxycycline	40	Finafloxacin	40	2.788552	1	9.493996E-02	4.326465	1	3.752411E-02	2.530106	S*, A
3	Average signs	≥6	27	Doxycycline	40	Finafloxacin	40	2.788552	1	9.493996E-02	4.326465	1	3.752411E-02	2.530106	S*, A
3	Total signs	≥6	28	Doxycycline	40	Finafloxacin	40	4.326465	1	3.752411E-02	4.326465	1	3.752411E-02	1.000000	S*, A*
3	Average signs	≥6	28	Doxycycline	40	Finafloxacin	40	4.326465	1	3.752411E-02	4.326465	1	3.752411E-02	1.000000	S*, A*
3	Total signs	≥6	29	Doxycycline	40	Finafloxacin	40	4.326465	1	3.752411E-02	4.326465	1	3.752411E-02	1.000000	S*, A*
3	Average signs	≥6	29	Doxycycline	40	Finafloxacin	40	4.326465	1	3.752411E-02	4.326465	1	3.752411E-02	1.000000	S*, A*
3	Total signs	≥6	30	Doxycycline	40	Finafloxacin	40	4.326465	1	3.752411E-02	4.326465	1	3.752411E-02	1.000000	S*, A*
3	Average signs	≥6	30	Doxycycline	40	Finafloxacin	40	4.326465	1	3.752411E-02	4.326465	1	3.752411E-02	1.000000	S*, A*
3	Total signs	≥6	20	Finafloxacin & Doxycycline	40	Diluent Control	15	46.872846	1	7.574496E-12	46.872846	1	7.574496E-12	1.000000	S*, A*

Study	Threshold Metric	Signs threshold	Weight threshold	Comparison Group A		Comparison Group B		Alternative endpoint Comparison			Original study comparison			P-value ratio	P-value ratio change
				Treatment	n	Treatment	n	Test statistic	df	p-value	Test statistic	df	p-value		
3	Average signs	≥6	20	Finafloxacin & Doxycycline	40	Diluent Control	15	46.872846	1	7.574496E-12	46.872846	1	7.574496E-12	1.000000	S*, A*
3	Total signs	≥6	21	Finafloxacin & Doxycycline	40	Diluent Control	15	46.872846	1	7.574496E-12	46.872846	1	7.574496E-12	1.000000	S*, A*
3	Average signs	≥6	21	Finafloxacin & Doxycycline	40	Diluent Control	15	46.872846	1	7.574496E-12	46.872846	1	7.574496E-12	1.000000	S*, A*
3	Total signs	≥6	22	Finafloxacin & Doxycycline	40	Diluent Control	15	46.872846	1	7.574496E-12	46.872846	1	7.574496E-12	1.000000	S*, A*
3	Average signs	≥6	22	Finafloxacin & Doxycycline	40	Diluent Control	15	46.872846	1	7.574496E-12	46.872846	1	7.574496E-12	1.000000	S*, A*
3	Total signs	≥6	23	Finafloxacin & Doxycycline	40	Diluent Control	15	46.872846	1	7.574496E-12	46.872846	1	7.574496E-12	1.000000	S*, A*
3	Average signs	≥6	23	Finafloxacin & Doxycycline	40	Diluent Control	15	46.872846	1	7.574496E-12	46.872846	1	7.574496E-12	1.000000	S*, A*
3	Total signs	≥6	24	Finafloxacin & Doxycycline	40	Diluent Control	15	46.872846	1	7.574496E-12	46.872846	1	7.574496E-12	1.000000	S*, A*
3	Average signs	≥6	24	Finafloxacin & Doxycycline	40	Diluent Control	15	46.872846	1	7.574496E-12	46.872846	1	7.574496E-12	1.000000	S*, A*
3	Total signs	≥6	25	Finafloxacin & Doxycycline	40	Diluent Control	15	46.872846	1	7.574496E-12	46.872846	1	7.574496E-12	1.000000	S*, A*
3	Average signs	≥6	25	Finafloxacin & Doxycycline	40	Diluent Control	15	46.872846	1	7.574496E-12	46.872846	1	7.574496E-12	1.000000	S*, A*
3	Total signs	≥6	26	Finafloxacin & Doxycycline	40	Diluent Control	15	46.872846	1	7.574496E-12	46.872846	1	7.574496E-12	1.000000	S*, A*
3	Average signs	≥6	26	Finafloxacin & Doxycycline	40	Diluent Control	15	46.872846	1	7.574496E-12	46.872846	1	7.574496E-12	1.000000	S*, A*
3	Total signs	≥6	27	Finafloxacin & Doxycycline	40	Diluent Control	15	46.872846	1	7.574496E-12	46.872846	1	7.574496E-12	1.000000	S*, A*
3	Average signs	≥6	27	Finafloxacin & Doxycycline	40	Diluent Control	15	46.872846	1	7.574496E-12	46.872846	1	7.574496E-12	1.000000	S*, A*
3	Total signs	≥6	28	Finafloxacin & Doxycycline	40	Diluent Control	15	46.872846	1	7.574496E-12	46.872846	1	7.574496E-12	1.000000	S*, A*
3	Average signs	≥6	28	Finafloxacin & Doxycycline	40	Diluent Control	15	46.872846	1	7.574496E-12	46.872846	1	7.574496E-12	1.000000	S*, A*
3	Total signs	≥6	29	Finafloxacin & Doxycycline	40	Diluent Control	15	46.872846	1	7.574496E-12	46.872846	1	7.574496E-12	1.000000	S*, A*
3	Average signs	≥6	29	Finafloxacin & Doxycycline	40	Diluent Control	15	46.872846	1	7.574496E-12	46.872846	1	7.574496E-12	1.000000	S*, A*
3	Total signs	≥6	30	Finafloxacin & Doxycycline	40	Diluent Control	15	46.872846	1	7.574496E-12	46.872846	1	7.574496E-12	1.000000	S*, A*
3	Average signs	≥6	30	Finafloxacin & Doxycycline	40	Diluent Control	15	46.872846	1	7.574496E-12	46.872846	1	7.574496E-12	1.000000	S*, A*
3	Total signs	≥6	20	Finafloxacin & Doxycycline	40	Finafloxacin	40	1.010082	1	3.148832E-01	0.709539	1	3.995968E-01	0.788002	S, A
3	Average signs	≥6	20	Finafloxacin & Doxycycline	40	Finafloxacin	40	1.010082	1	3.148832E-01	0.709539	1	3.995968E-01	0.788002	S, A
3	Total signs	≥6	21	Finafloxacin & Doxycycline	40	Finafloxacin	40	1.022745	1	3.118688E-01	0.709539	1	3.995968E-01	0.780459	S, A
3	Average signs	≥6	21	Finafloxacin & Doxycycline	40	Finafloxacin	40	1.022745	1	3.118688E-01	0.709539	1	3.995968E-01	0.780459	S, A
3	Total signs	≥6	22	Finafloxacin & Doxycycline	40	Finafloxacin	40	1.004097	1	3.163213E-01	0.709539	1	3.995968E-01	0.791601	S, A

Study	Threshold Metric	Signs threshold	Weight threshold	Comparison Group A		Comparison Group B		Alternative endpoint Comparison			Original study comparison			P-value ratio	P-value ratio change
				Treatment	n	Treatment	n	Test statistic	df	p-value	Test statistic	df	p-value		
3	Average signs	≥6	22	Finafloxacin & Doxycycline	40	Finafloxacin	40	1.004097	1	3.163213E-01	0.709539	1	3.995968E-01	0.791601	S, A
3	Total signs	≥6	23	Finafloxacin & Doxycycline	40	Finafloxacin	40	0.543344	1	4.610503E-01	0.709539	1	3.995968E-01	1.153789	S, A
3	Average signs	≥6	23	Finafloxacin & Doxycycline	40	Finafloxacin	40	0.543344	1	4.610503E-01	0.709539	1	3.995968E-01	1.153789	S, A
3	Total signs	≥6	24	Finafloxacin & Doxycycline	40	Finafloxacin	40	1.087494	1	2.970271E-01	0.709539	1	3.995968E-01	0.743317	S, A
3	Average signs	≥6	24	Finafloxacin & Doxycycline	40	Finafloxacin	40	1.087494	1	2.970271E-01	0.709539	1	3.995968E-01	0.743317	S, A
3	Total signs	≥6	25	Finafloxacin & Doxycycline	40	Finafloxacin	40	1.101193	1	2.940044E-01	0.709539	1	3.995968E-01	0.735753	S, A
3	Average signs	≥6	25	Finafloxacin & Doxycycline	40	Finafloxacin	40	1.101193	1	2.940044E-01	0.709539	1	3.995968E-01	0.735753	S, A
3	Total signs	≥6	26	Finafloxacin & Doxycycline	40	Finafloxacin	40	0.527210	1	4.677824E-01	0.709539	1	3.995968E-01	1.170636	S, A
3	Average signs	≥6	26	Finafloxacin & Doxycycline	40	Finafloxacin	40	0.527210	1	4.677824E-01	0.709539	1	3.995968E-01	1.170636	S, A
3	Total signs	≥6	27	Finafloxacin & Doxycycline	40	Finafloxacin	40	1.142105	1	2.852081E-01	0.709539	1	3.995968E-01	0.713740	S, A
3	Average signs	≥6	27	Finafloxacin & Doxycycline	40	Finafloxacin	40	1.142105	1	2.852081E-01	0.709539	1	3.995968E-01	0.713740	S, A
3	Total signs	≥6	28	Finafloxacin & Doxycycline	40	Finafloxacin	40	1.403252	1	2.361798E-01	0.709539	1	3.995968E-01	0.591045	S, A
3	Average signs	≥6	28	Finafloxacin & Doxycycline	40	Finafloxacin	40	1.403252	1	2.361798E-01	0.709539	1	3.995968E-01	0.591045	S, A
3	Total signs	≥6	29	Finafloxacin & Doxycycline	40	Finafloxacin	40	1.403252	1	2.361798E-01	0.709539	1	3.995968E-01	0.591045	S, A
3	Average signs	≥6	29	Finafloxacin & Doxycycline	40	Finafloxacin	40	1.403252	1	2.361798E-01	0.709539	1	3.995968E-01	0.591045	S, A
3	Total signs	≥6	30	Finafloxacin & Doxycycline	40	Finafloxacin	40	0.709539	1	3.995968E-01	0.709539	1	3.995968E-01	1.000000	S, A
3	Average signs	≥6	30	Finafloxacin & Doxycycline	40	Finafloxacin	40	0.709539	1	3.995968E-01	0.709539	1	3.995968E-01	1.000000	S, A
3	Total signs	≥6	20	Finafloxacin & Doxycycline	40	Doxycycline	40	2.194010	1	1.385482E-01	1.597411	1	2.062705E-01	0.671682	S, A
3	Average signs	≥6	20	Finafloxacin & Doxycycline	40	Doxycycline	40	2.194010	1	1.385482E-01	1.597411	1	2.062705E-01	0.671682	S, A
3	Total signs	≥6	21	Finafloxacin & Doxycycline	40	Doxycycline	40	1.524480	1	2.169427E-01	1.597411	1	2.062705E-01	1.051739	S, A
3	Average signs	≥6	21	Finafloxacin & Doxycycline	40	Doxycycline	40	1.524480	1	2.169427E-01	1.597411	1	2.062705E-01	1.051739	S, A
3	Total signs	≥6	22	Finafloxacin & Doxycycline	40	Doxycycline	40	1.507345	1	2.195447E-01	1.597411	1	2.062705E-01	1.064353	S, A
3	Average signs	≥6	22	Finafloxacin & Doxycycline	40	Doxycycline	40	1.507345	1	2.195447E-01	1.597411	1	2.062705E-01	1.064353	S, A
3	Total signs	≥6	23	Finafloxacin & Doxycycline	40	Doxycycline	40	1.123972	1	2.890648E-01	1.597411	1	2.062705E-01	1.401387	S, A
3	Average signs	≥6	23	Finafloxacin & Doxycycline	40	Doxycycline	40	1.123972	1	2.890648E-01	1.597411	1	2.062705E-01	1.401387	S, A
3	Total signs	≥6	24	Finafloxacin & Doxycycline	40	Doxycycline	40	1.077672	1	2.992189E-01	1.597411	1	2.062705E-01	1.450614	S, A

Study	Threshold Metric	Signs threshold	Weight threshold	Comparison Group A		Comparison Group B		Alternative endpoint Comparison			Original study comparison			P-value ratio	P-value ratio change
				Treatment	n	Treatment	n	Test statistic	df	p-value	Test statistic	df	p-value		
3	Average signs	≥6	24	Finafloxacin & Doxycycline	40	Doxycycline	40	1.077672	1	2.992189E-01	1.597411	1	2.062705E-01	1.450614	S, A
3	Total signs	≥6	25	Finafloxacin & Doxycycline	40	Doxycycline	40	0.660460	1	4.163976E-01	1.597411	1	2.062705E-01	2.018697	S, A
3	Average signs	≥6	25	Finafloxacin & Doxycycline	40	Doxycycline	40	0.660460	1	4.163976E-01	1.597411	1	2.062705E-01	2.018697	S, A
3	Total signs	≥6	26	Finafloxacin & Doxycycline	40	Doxycycline	40	0.768297	1	3.807445E-01	1.597411	1	2.062705E-01	1.845851	S, A
3	Average signs	≥6	26	Finafloxacin & Doxycycline	40	Doxycycline	40	0.768297	1	3.807445E-01	1.597411	1	2.062705E-01	1.845851	S, A
3	Total signs	≥6	27	Finafloxacin & Doxycycline	40	Doxycycline	40	0.354552	1	5.515474E-01	1.597411	1	2.062705E-01	2.673904	S, A
3	Average signs	≥6	27	Finafloxacin & Doxycycline	40	Doxycycline	40	0.354552	1	5.515474E-01	1.597411	1	2.062705E-01	2.673904	S, A
3	Total signs	≥6	28	Finafloxacin & Doxycycline	40	Doxycycline	40	0.832075	1	3.616731E-01	1.597411	1	2.062705E-01	1.753392	S, A
3	Average signs	≥6	28	Finafloxacin & Doxycycline	40	Doxycycline	40	0.832075	1	3.616731E-01	1.597411	1	2.062705E-01	1.753392	S, A
3	Total signs	≥6	29	Finafloxacin & Doxycycline	40	Doxycycline	40	0.832075	1	3.616731E-01	1.597411	1	2.062705E-01	1.753392	S, A
3	Average signs	≥6	29	Finafloxacin & Doxycycline	40	Doxycycline	40	0.832075	1	3.616731E-01	1.597411	1	2.062705E-01	1.753392	S, A
3	Total signs	≥6	30	Finafloxacin & Doxycycline	40	Doxycycline	40	1.597411	1	2.062705E-01	1.597411	1	2.062705E-01	1.000000	S, A
3	Average signs	≥6	30	Finafloxacin & Doxycycline	40	Doxycycline	40	1.597411	1	2.062705E-01	1.597411	1	2.062705E-01	1.000000	S, A
4	Total signs	≥6	20	Finafloxacin 36 h	45	Finafloxacin 48 h	44	0.445966	1	5.042569E-01	1.730962	1	1.882881E-01	2.678114	S, A
4	Average signs	≥6	20	Finafloxacin 36 h	45	Finafloxacin 48 h	44	0.445966	1	5.042569E-01	1.730962	1	1.882881E-01	2.678114	S, A
4	Total signs	≥6	21	Finafloxacin 36 h	45	Finafloxacin 48 h	44	0.235782	1	6.272695E-01	1.730962	1	1.882881E-01	3.331435	S, A
4	Average signs	≥6	21	Finafloxacin 36 h	45	Finafloxacin 48 h	44	0.235782	1	6.272695E-01	1.730962	1	1.882881E-01	3.331435	S, A
4	Total signs	≥6	22	Finafloxacin 36 h	45	Finafloxacin 48 h	44	0.454321	1	5.002902E-01	1.730962	1	1.882881E-01	2.657047	S, A
4	Average signs	≥6	22	Finafloxacin 36 h	45	Finafloxacin 48 h	44	0.454321	1	5.002902E-01	1.730962	1	1.882881E-01	2.657047	S, A
4	Total signs	≥6	23	Finafloxacin 36 h	45	Finafloxacin 48 h	44	0.210916	1	6.460507E-01	1.730962	1	1.882881E-01	3.431182	S, A
4	Average signs	≥6	23	Finafloxacin 36 h	45	Finafloxacin 48 h	44	0.211535	1	6.455672E-01	1.730962	1	1.882881E-01	3.428615	S, A
4	Total signs	≥6	24	Finafloxacin 36 h	45	Finafloxacin 48 h	44	0.309805	1	5.777999E-01	1.730962	1	1.882881E-01	3.068702	S, A
4	Average signs	≥6	24	Finafloxacin 36 h	45	Finafloxacin 48 h	44	0.570395	1	4.501019E-01	1.730962	1	1.882881E-01	2.390496	S, A
4	Total signs	≥6	25	Finafloxacin 36 h	45	Finafloxacin 48 h	44	0.607727	1	4.356448E-01	1.730962	1	1.882881E-01	2.313714	S, A
4	Average signs	≥6	25	Finafloxacin 36 h	45	Finafloxacin 48 h	44	0.960632	1	3.270278E-01	1.730962	1	1.882881E-01	1.736848	S, A
4	Total signs	≥6	26	Finafloxacin 36 h	45	Finafloxacin 48 h	44	0.616350	1	4.324069E-01	1.730962	1	1.882881E-01	2.296518	S, A
4	Average signs	≥6	26	Finafloxacin 36 h	45	Finafloxacin 48 h	44	0.971426	1	3.243248E-01	1.730962	1	1.882881E-01	1.722493	S, A
4	Total signs	≥6	27	Finafloxacin 36 h	45	Finafloxacin 48 h	44	0.598801	1	4.390357E-01	1.730962	1	1.882881E-01	2.331723	S, A
4	Average signs	≥6	27	Finafloxacin 36 h	45	Finafloxacin 48 h	44	0.949191	1	3.299252E-01	1.730962	1	1.882881E-01	1.752236	S, A
4	Total signs	≥6	28	Finafloxacin 36 h	45	Finafloxacin 48 h	44	0.876175	1	3.492515E-01	1.730962	1	1.882881E-01	1.854878	S, A
4	Average signs	≥6	28	Finafloxacin 36 h	45	Finafloxacin 48 h	44	1.281959	1	2.575351E-01	1.730962	1	1.882881E-01	1.367772	S, A
4	Total signs	≥6	29	Finafloxacin 36 h	45	Finafloxacin 48 h	44	1.283321	1	2.572826E-01	1.730962	1	1.882881E-01	1.366430	S, A

Study	Threshold Metric	Signs threshold	Weight threshold	Comparison Group A		Comparison Group B		Alternative endpoint Comparison			Original study comparison			P-value ratio	P-value ratio change
				Treatment	n	Treatment	n	Test statistic	df	p-value	Test statistic	df	p-value		
4	Average signs	≥6	29	Finafloxacin 36 h	45	Finafloxacin 48 h	44	1.785758	1	1.814439E-01	1.730962	1	1.882881E-01	0.963650	S, A
4	Total signs	≥6	30	Finafloxacin 36 h	45	Finafloxacin 48 h	44	1.253376	1	2.629086E-01	1.730962	1	1.882881E-01	1.396310	S, A
4	Average signs	≥6	30	Finafloxacin 36 h	45	Finafloxacin 48 h	44	1.749705	1	1.859138E-01	1.730962	1	1.882881E-01	0.987390	S, A
4	Total signs	≥6	20	Finafloxacin 36 h	45	Diluent Control	88	94.766888	1	2.141784E-22	94.766888	1	2.141784E-22	1.000000	S*, A*
4	Average signs	≥6	20	Finafloxacin 36 h	45	Diluent Control	88	94.766888	1	2.141784E-22	94.766888	1	2.141784E-22	1.000000	S*, A*
4	Total signs	≥6	21	Finafloxacin 36 h	45	Diluent Control	88	94.766888	1	2.141784E-22	94.766888	1	2.141784E-22	1.000000	S*, A*
4	Average signs	≥6	21	Finafloxacin 36 h	45	Diluent Control	88	94.766888	1	2.141784E-22	94.766888	1	2.141784E-22	1.000000	S*, A*
4	Total signs	≥6	22	Finafloxacin 36 h	45	Diluent Control	88	94.766888	1	2.141784E-22	94.766888	1	2.141784E-22	1.000000	S*, A*
4	Average signs	≥6	22	Finafloxacin 36 h	45	Diluent Control	88	94.766888	1	2.141784E-22	94.766888	1	2.141784E-22	1.000000	S*, A*
4	Total signs	≥6	23	Finafloxacin 36 h	45	Diluent Control	88	94.766888	1	2.141784E-22	94.766888	1	2.141784E-22	1.000000	S*, A*
4	Average signs	≥6	23	Finafloxacin 36 h	45	Diluent Control	88	94.766888	1	2.141784E-22	94.766888	1	2.141784E-22	1.000000	S*, A*
4	Total signs	≥6	24	Finafloxacin 36 h	45	Diluent Control	88	94.766888	1	2.141784E-22	94.766888	1	2.141784E-22	1.000000	S*, A*
4	Average signs	≥6	24	Finafloxacin 36 h	45	Diluent Control	88	94.766888	1	2.141784E-22	94.766888	1	2.141784E-22	1.000000	S*, A*
4	Total signs	≥6	25	Finafloxacin 36 h	45	Diluent Control	88	94.766888	1	2.141784E-22	94.766888	1	2.141784E-22	1.000000	S*, A*
4	Average signs	≥6	25	Finafloxacin 36 h	45	Diluent Control	88	94.766888	1	2.141784E-22	94.766888	1	2.141784E-22	1.000000	S*, A*
4	Total signs	≥6	26	Finafloxacin 36 h	45	Diluent Control	88	94.766888	1	2.141784E-22	94.766888	1	2.141784E-22	1.000000	S*, A*
4	Average signs	≥6	26	Finafloxacin 36 h	45	Diluent Control	88	94.766888	1	2.141784E-22	94.766888	1	2.141784E-22	1.000000	S*, A*
4	Total signs	≥6	27	Finafloxacin 36 h	45	Diluent Control	88	94.766888	1	2.141784E-22	94.766888	1	2.141784E-22	1.000000	S*, A*
4	Average signs	≥6	27	Finafloxacin 36 h	45	Diluent Control	88	94.766888	1	2.141784E-22	94.766888	1	2.141784E-22	1.000000	S*, A*
4	Total signs	≥6	28	Finafloxacin 36 h	45	Diluent Control	88	94.766888	1	2.141784E-22	94.766888	1	2.141784E-22	1.000000	S*, A*
4	Average signs	≥6	28	Finafloxacin 36 h	45	Diluent Control	88	94.766888	1	2.141784E-22	94.766888	1	2.141784E-22	1.000000	S*, A*
4	Total signs	≥6	29	Finafloxacin 36 h	45	Diluent Control	88	94.766888	1	2.141784E-22	94.766888	1	2.141784E-22	1.000000	S*, A*
4	Average signs	≥6	29	Finafloxacin 36 h	45	Diluent Control	88	94.766888	1	2.141784E-22	94.766888	1	2.141784E-22	1.000000	S*, A*
4	Total signs	≥6	30	Finafloxacin 36 h	45	Diluent Control	88	94.766888	1	2.141784E-22	94.766888	1	2.141784E-22	1.000000	S*, A*
4	Average signs	≥6	30	Finafloxacin 36 h	45	Diluent Control	88	94.766888	1	2.141784E-22	94.766888	1	2.141784E-22	1.000000	S*, A*
4	Total signs	≥6	20	Finafloxacin 48 h	44	Diluent Control	88	72.966845	1	1.318462E-17	72.966845	1	1.318462E-17	1.000000	S*, A*
4	Average signs	≥6	20	Finafloxacin 48 h	44	Diluent Control	88	72.966845	1	1.318462E-17	72.966845	1	1.318462E-17	1.000000	S*, A*
4	Total signs	≥6	21	Finafloxacin 48 h	44	Diluent Control	88	72.966845	1	1.318462E-17	72.966845	1	1.318462E-17	1.000000	S*, A*
4	Average signs	≥6	21	Finafloxacin 48 h	44	Diluent Control	88	72.966845	1	1.318462E-17	72.966845	1	1.318462E-17	1.000000	S*, A*
4	Total signs	≥6	22	Finafloxacin 48 h	44	Diluent Control	88	72.966845	1	1.318462E-17	72.966845	1	1.318462E-17	1.000000	S*, A*
4	Average signs	≥6	22	Finafloxacin 48 h	44	Diluent Control	88	72.966845	1	1.318462E-17	72.966845	1	1.318462E-17	1.000000	S*, A*
4	Total signs	≥6	23	Finafloxacin 48 h	44	Diluent Control	88	72.966845	1	1.318462E-17	72.966845	1	1.318462E-17	1.000000	S*, A*
4	Average signs	≥6	23	Finafloxacin 48 h	44	Diluent Control	88	72.966845	1	1.318462E-17	72.966845	1	1.318462E-17	1.000000	S*, A*
4	Total signs	≥6	24	Finafloxacin 48 h	44	Diluent Control	88	72.966845	1	1.318462E-17	72.966845	1	1.318462E-17	1.000000	S*, A*
4	Average signs	≥6	24	Finafloxacin 48 h	44	Diluent Control	88	72.966845	1	1.318462E-17	72.966845	1	1.318462E-17	1.000000	S*, A*
4	Total signs	≥6	25	Finafloxacin 48 h	44	Diluent Control	88	72.966845	1	1.318462E-17	72.966845	1	1.318462E-17	1.000000	S*, A*
4	Average signs	≥6	25	Finafloxacin 48 h	44	Diluent Control	88	72.966845	1	1.318462E-17	72.966845	1	1.318462E-17	1.000000	S*, A*
4	Total signs	≥6	26	Finafloxacin 48 h	44	Diluent Control	88	72.966845	1	1.318462E-17	72.966845	1	1.318462E-17	1.000000	S*, A*

Study	Threshold Metric	Signs threshold	Weight threshold	Comparison Group A		Comparison Group B		Alternative endpoint Comparison			Original study comparison			P-value ratio	P-value ratio change
				Treatment	n	Treatment	n	Test statistic	df	p-value	Test statistic	df	p-value		
4	Average signs	≥6	26	Finafloxacin 48 h	44	Diluent Control	88	72.966845	1	1.318462E-17	72.966845	1	1.318462E-17	1.000000	S*, A*
4	Total signs	≥6	27	Finafloxacin 48 h	44	Diluent Control	88	72.966845	1	1.318462E-17	72.966845	1	1.318462E-17	1.000000	S*, A*
4	Average signs	≥6	27	Finafloxacin 48 h	44	Diluent Control	88	72.966845	1	1.318462E-17	72.966845	1	1.318462E-17	1.000000	S*, A*
4	Total signs	≥6	28	Finafloxacin 48 h	44	Diluent Control	88	72.966845	1	1.318462E-17	72.966845	1	1.318462E-17	1.000000	S*, A*
4	Average signs	≥6	28	Finafloxacin 48 h	44	Diluent Control	88	72.966845	1	1.318462E-17	72.966845	1	1.318462E-17	1.000000	S*, A*
4	Total signs	≥6	29	Finafloxacin 48 h	44	Diluent Control	88	72.966845	1	1.318462E-17	72.966845	1	1.318462E-17	1.000000	S*, A*
4	Average signs	≥6	29	Finafloxacin 48 h	44	Diluent Control	88	72.966845	1	1.318462E-17	72.966845	1	1.318462E-17	1.000000	S*, A*
4	Total signs	≥6	30	Finafloxacin 48 h	44	Diluent Control	88	72.966845	1	1.318462E-17	72.966845	1	1.318462E-17	1.000000	S*, A*
4	Average signs	≥6	30	Finafloxacin 48 h	44	Diluent Control	88	72.966845	1	1.318462E-17	72.966845	1	1.318462E-17	1.000000	S*, A*

n = number of mice, df = degrees of freedom, S* = Significant difference in original study comparison, A* = Significant difference in alternative comparison, S = No significant difference in original study comparison, A = No significant difference in alternative comparison.

Supplementary Table 4.

Summary of results across the humane endpoints evaluated and derived according to weight loss threshold values, grouped by study, total signs and sign threshold values. Results are limited to signs thresholds of ≥4 and ≥5, and weight thresholds values between 23% to 28% weight loss. Rows highlighted in dark grey indicate the most suitable refined endpoint criteria (‘average total signs’ threshold of ≥5 with 25% weight loss). Rows highlighted in light grey indicate the corresponding outiering weight threshold values of this refined endpoint criteria (‘average total signs’ threshold of ≥5 with either 23% or 28% weight loss).

Study	Threshold Metric	Signs Threshold	Weight threshold	Number of mice	# (%) False Positives	Specificity	Median days saved	Median sign days saved	Number of comparisons	Number (%) comparison changes
1	Average signs	≥4	23	105	15 (14.3%)	0.776	10.3	58.7	3	0 (0%)
	Average signs	≥4	24		15 (14.3%)	0.776	10.3	58.7		0 (0%)
	Average signs	≥4	25		15 (14.3%)	0.776	10.3	58.7		0 (0%)
	Average signs	≥4	26		15 (14.3%)	0.776	10.3	58.7		0 (0%)
	Average signs	≥4	27		14 (13.3%)	0.791	10.2	58.7		0 (0%)
	Average signs	≥4	28		13 (12.4%)	0.806	10.2	58.7		0 (0%)
	Average signs	≥5	23		14 (13.3%)	0.791	9.7	56.4		0 (0%)
	Average signs	≥5	24		14 (13.3%)	0.791	9.7	56.4		0 (0%)
	Average signs	≥5	25		14 (13.3%)	0.791	9.7	56.4		0 (0%)
	Average signs	≥5	26		14 (13.3%)	0.791	9.7	56.4		0 (0%)
	Average signs	≥5	27		13 (12.4%)	0.806	9.5	56.4		0 (0%)
	Average signs	≥5	28		12 (11.4%)	0.821	9.5	56.4		0 (0%)
	Total signs	≥4	23		16 (15.2%)	0.761	13.3	62.2		0 (0%)
	Total signs	≥4	24		16 (15.2%)	0.761	13.3	62.2		0 (0%)
	Total signs	≥4	25		16 (15.2%)	0.761	13.3	62.2		0 (0%)
	Total signs	≥4	26		16 (15.2%)	0.761	13.3	62.2		0 (0%)
	Total signs	≥4	27		15 (14.3%)	0.776	11.0	62.2		0 (0%)
	Total signs	≥4	28		14 (13.3%)	0.791	11.0	62.2		0 (0%)
	Total signs	≥5	23		16 (15.2%)	0.761	13.3	62.2		0 (0%)

Study	Threshold Metric	Signs Threshold	Weight threshold	Number of mice	# (%) False Positives	Specificity	Median days saved	Median sign days saved	Number of comparisons	Number (%) comparison changes
	Total signs	≥5	24		16 (15.2%)	0.761	13.3	62.2		0 (0%)
	Total signs	≥5	25		16 (15.2%)	0.761	13.3	62.2		0 (0%)
	Total signs	≥5	26		16 (15.2%)	0.761	13.3	62.2		0 (0%)
	Total signs	≥5	27		15 (14.3%)	0.776	11.0	62.2		0 (0%)
	Total signs	≥5	28		14 (13.3%)	0.791	11.0	62.2		0 (0%)
2	Average signs	≥4	23	110	7 (6.4%)	0.924	9.7	32.5	1	0 (0%)
	Average signs	≥4	24		7 (6.4%)	0.924	9.7	32.5		0 (0%)
	Average signs	≥4	25		7 (6.4%)	0.924	9.7	32.5		0 (0%)
	Average signs	≥4	26		7 (6.4%)	0.924	9.7	32.5		0 (0%)
	Average signs	≥4	27		7 (6.4%)	0.924	9.7	32.5		0 (0%)
	Average signs	≥4	28		7 (6.4%)	0.924	9.7	32.5		0 (0%)
	Average signs	≥5	23		3 (2.7%)	0.967	3.5	18.0		0 (0%)
	Average signs	≥5	24		3 (2.7%)	0.967	3.0	15.5		0 (0%)
	Average signs	≥5	25		3 (2.7%)	0.967	3.0	15.5		0 (0%)
	Average signs	≥5	26		3 (2.7%)	0.967	3.0	15.5		0 (0%)
	Average signs	≥5	27		3 (2.7%)	0.967	3.0	15.5		0 (0%)
	Average signs	≥5	28		3 (2.7%)	0.967	3.0	15.5		0 (0%)
	Total signs	≥4	23		18 (16.4%)	0.804	15.0	53.7		0 (0%)
	Total signs	≥4	24		18 (16.4%)	0.804	15.0	53.7		0 (0%)
	Total signs	≥4	25		18 (16.4%)	0.804	15.0	53.7		0 (0%)
	Total signs	≥4	26		18 (16.4%)	0.804	15.0	53.7		0 (0%)
	Total signs	≥4	27		18 (16.4%)	0.804	15.0	53.7		0 (0%)
	Total signs	≥4	28		18 (16.4%)	0.804	15.0	53.7		0 (0%)
	Total signs	≥5	23		5 (4.5%)	0.946	5.0	24.5		0 (0%)
	Total signs	≥5	24		5 (4.5%)	0.946	5.0	24.5		0 (0%)
	Total signs	≥5	25		5 (4.5%)	0.946	5.0	24.5		0 (0%)
	Total signs	≥5	26		5 (4.5%)	0.946	5.0	24.5		0 (0%)
	Total signs	≥5	27		5 (4.5%)	0.946	5.0	24.5		0 (0%)
	Total signs	≥5	28		5 (4.5%)	0.946	5.0	24.5		0 (0%)
3	Average signs	≥4	23	135	13 (9.6%)	0.888	5.8	8.1	6	0 (0%)
	Average signs	≥4	24		12 (8.9%)	0.897	6.7	8.1		0 (0%)
	Average signs	≥4	25		11 (8.1%)	0.905	5.8	8.1		0 (0%)
	Average signs	≥4	26		9 (6.7%)	0.922	4.1	8.1		0 (0%)
	Average signs	≥4	27		7 (5.2%)	0.94	2.5	8.1		0 (0%)
	Average signs	≥4	28		5 (3.7%)	0.957	2.0	8.1		0 (0%)
	Average signs	≥5	23		10 (7.4%)	0.914	7.0	6.9		0 (0%)
	Average signs	≥5	24		9 (6.7%)	0.922	6.7	6.9		0 (0%)
	Average signs	≥5	25		8 (5.9%)	0.931	5.0	14.2		0 (0%)
	Average signs	≥5	26		6 (4.4%)	0.948	4.1	11.6		1 (16.7%)
	Average signs	≥5	27		4 (3%)	0.966	2.5	4.3		1 (16.7%)
	Average signs	≥5	28		2 (1.5%)	0.983	1.7	25.0		0 (0%)
	Total signs	≥4	23		19 (14.1%)	0.836	9.0	16.9		1 (16.7%)
	Total signs	≥4	24		19 (14.1%)	0.836	8.3	16.9		1 (16.7%)
	Total signs	≥4	25		18 (13.3%)	0.845	7.0	16.9		1 (16.7%)
	Total signs	≥4	26		16 (11.9%)	0.862	7.0	16.9		2 (33.3%)
	Total signs	≥4	27		16 (11.9%)	0.862	7.0	16.9		2 (33.3%)

Study	Threshold Metric	Signs Threshold	Weight threshold	Number of mice	# (%) False Positives	Specificity	Median days saved	Median sign days saved	Number of comparisons	Number (%) comparison changes
	Total signs	≥4	28		14 (10.4%)	0.879	7.0	16.9		1 (16.7%)
	Total signs	≥5	23		14 (10.4%)	0.879	7.0	6.9		0 (0%)
	Total signs	≥5	24		13 (9.6%)	0.888	7.0	6.9		0 (0%)
	Total signs	≥5	25		12 (8.9%)	0.897	6.2	14.2		0 (0%)
	Total signs	≥5	26		10 (7.4%)	0.914	4.1	11.6		0 (0%)
	Total signs	≥5	27		8 (5.9%)	0.931	2.5	32.6		0 (0%)
	Total signs	≥5	28		6 (4.4%)	0.948	1.7	3.5		1 (16.7%)
4	Average signs	≥4	23	206	14 (6.8%)	0.886	4.2	14.0	3	0 (0%)
	Average signs	≥4	24		13 (6.3%)	0.894	3.6	13.7		0 (0%)
	Average signs	≥4	25		13 (6.3%)	0.894	3.2	9.6		0 (0%)
	Average signs	≥4	26		13 (6.3%)	0.894	2.3	9.6		0 (0%)
	Average signs	≥4	27		13 (6.3%)	0.894	2.3	9.6		0 (0%)
	Average signs	≥4	28		13 (6.3%)	0.894	2.0	9.3		0 (0%)
	Average signs	≥5	23		11 (5.3%)	0.911	4.2	10.9		0 (0%)
	Average signs	≥5	24		6 (2.9%)	0.951	2.8	9.0		0 (0%)
	Average signs	≥5	25		4 (1.9%)	0.967	2.5	7.8		0 (0%)
	Average signs	≥5	26		4 (1.9%)	0.967	2.2	5.7		0 (0%)
	Average signs	≥5	27		4 (1.9%)	0.967	1.2	4.0		0 (0%)
	Average signs	≥5	28		3 (1.5%)	0.976	0.5	2.0		0 (0%)
	Total signs	≥4	23		18 (8.7%)	0.854	5.2	15.3		0 (0%)
	Total signs	≥4	24		17 (8.3%)	0.862	4.3	14.5		0 (0%)
	Total signs	≥4	25		17 (8.3%)	0.862	4.3	14.5		0 (0%)
	Total signs	≥4	26		17 (8.3%)	0.862	4.3	14.5		0 (0%)
	Total signs	≥4	27		17 (8.3%)	0.862	4.3	14.5		0 (0%)
	Total signs	≥4	28		17 (8.3%)	0.862	4.3	14.5		0 (0%)
	Total signs	≥5	23		14 (6.8%)	0.886	3.8	9.3		0 (0%)
	Total signs	≥5	24		12 (5.8%)	0.902	2.8	9.0		0 (0%)
	Total signs	≥5	25		11 (5.3%)	0.911	2.5	7.4		0 (0%)
	Total signs	≥5	26		11 (5.3%)	0.911	2.2	6.4		0 (0%)
	Total signs	≥5	27		11 (5.3%)	0.911	1.6	5.4		0 (0%)
	Total signs	≥5	28		10 (4.9%)	0.919	1.0	4.3		0 (0%)

Supplementary Table 5.

Clinical Score Criteria for studies 1 and 2. Animals were euthanised immediately if they displayed any of the signs highlighted pink. In addition, for study 2, animals were also euthanised immediately if the total score reached 6 (whichever occurred first).

Score	Coat	Posture	Eyes	Locomotion	Mobility/Activity	Respiration
1	Visible starring around neck		Animal has issues with one or both eyes but is able to open at least one eye	Animal holds one rear leg high and rarely uses it for movement. Animal still able to move around cage fairly easily.	Movement is slow. There may be visible limb issues.	Breathing is slightly fast or slightly slow
2	Fur is ruffled	Animal displays hunched posture	Animal has issues with both eyes and does not open eyes despite handling the animal.	Animal has issues with both rear legs. Movement is significantly restricted.	Animal is immobile and does not move despite handling the animal.	Breathing is extremely laboured and/or rate of respiration is really fast or really slow

Supplementary Table 6.

Clinical Score Criteria for studies 3 and 4. Animals were euthanised immediately if they displayed any of the signs highlighted pink, or if the total score reached 6 (whichever occurred first). Mapping to the 2-score criteria shown in square brackets.

Score	Coat	Posture	Eyes	Activity	Breathing	Neurological	Weight
1			Eye(s) partly closed **[1]**	Animal is active and interested in the environment but noticeably slower than normal **[1]**		Includes:<br>• Involuntary shaking of whole body<br>• Circling or spinning with seeming inability to stop<br>• Twitching or tilting of the head<br>• Seizures	Animal has lost more than 30% of pre-exposure weight recorded on two consecutive days.
2	Starring of fur – whole body **[1]**	Hunched posture when sitting or moving **[1]**	One eye is fully closed despite handling the animal **[1]**	Animal is reluctant to move without handling and shows limited interest in the environment. Movement can be slow and unsteady or gait highly abnormal **[2]**	Altered rate of breathing **[1]**		
3	Skin is clearly observable beneath fur **[2]**	Extremely hunched posture **[2]**	Both eyes fully closed despite handling the animal. **[2]**	Animal is barely able or unable to move despite handling. **[2]**	Breathing is laboured and has an abdominal component. **[2]**		

Supplementary Table 7.
Treatment group comparisons for each of the four studies. In each case, a comparison is made between the mice receiving the named treatment listed in group A versus the mice receiving the named treatment listed in group B.

Study	Treatment Group A	Treatment Group B
Study 1	Finafloxacin, n= 45	Diluent Control, n= 15
	Co-trimoxazole, n= 45	Diluent Control, n= 15
	Co-trimoxazole, n= 45	Finafloxacin, n= 45
Study 2	Finafloxacin, n= 90	Diluent Control, n= 20
Study 3	Finafloxacin, n= 40	Diluent Control, n= 15
	Doxycycline, n= 40	Diluent Control, n= 15
	Doxycycline, n= 40	Finafloxacin, n= 40
	Finafloxacin & doxycycline, n= 40	Diluent Control, n= 15
	Finafloxacin & doxycycline, n= 40	Finafloxacin, n= 40
	Finafloxacin & doxycycline, n= 40	Doxycycline, n= 40
Study 4	Finafloxacin, n = 45 (36h)	Finafloxacin, n = 44 (48h)
	Finafloxacin, n = 45 (36h)	Diluent Control, n= 88
	Finafloxacin, n = 44 (48h)	Diluent Control, n = 88

Copyright: © Crown copyright (2025), Dstl. This material is licensed under the terms of the Open Government Licence except where otherwise stated. To view this licence, visit http://www.nationalarchives.gov.uk/doc/open-government-licence/version/3 or write to the Information Policy Team, The National Archives, Kew, London TW9 4DU, or email: psi@nationalarchives.gov.uk.
